# The cranial anatomy and relationships of *Cardiocorax mukulu* (Plesiosauria: Elasmosauridae) from Bentiaba, Angola

**DOI:** 10.1371/journal.pone.0255773

**Published:** 2021-08-17

**Authors:** Miguel P. Marx, Octávio Mateus, Michael J. Polcyn, Anne S. Schulp, A. Olímpio Gonçalves, Louis L. Jacobs

**Affiliations:** 1 Huffington Department of Earth Sciences, ISEM at Southern Methodist University, Dallas, Texas, United States of America; 2 GeoBioTec + Faculdade de Ciências e Tecnologia, Universidade Nova de Lisboa, Caparica, Portugal; 3 Museu da Lourinhã, Lourinhã, Portugal; 4 Naturalis Biodiversity Center, Leiden, The Netherlands; 5 Department of Earth Sciences, Faculty of Geosciences, Utrecht University, Utrecht, The Netherlands; 6 Departamento de Geologia, Faculdade de Ciências, Universidade Agostinho Neto, Luanda, Angola; University College London, UNITED KINGDOM

## Abstract

We report a new specimen of the plesiosaur *Cardiocorax mukulu* that includes the most complete plesiosaur skull from sub-Saharan Africa. The well-preserved three-dimensional nature of the skull offers rare insight into the cranial anatomy of elasmosaurid plesiosaurians. The new specimen of *Cardiocorax mukulu* was recovered from Bentiaba, Namibe Province in Angola, approximately three meters above the holotype. The new specimen also includes an atlas-axis complex, seventeen postaxial cervical vertebrae, partial ribs, a femur, and limb elements. It is identified as *Cardiocorax mukulu* based on an apomorphy shared with the holotype where the cervical neural spine is approximately as long anteroposteriorly as the centrum and exhibits a sinusoidal anterior margin. The new specimen is nearly identical to the holotype and previously referred material in all other aspects. *Cardiocorax mukulu* is returned in an early-branching or intermediate position in Elasmosauridae in four out of the six of our phylogenetic analyses. *Cardiocorax mukulu* lacks the elongated cervical vertebrae that is characteristic of the extremely long-necked elasmosaurines, and the broad skull with and a high number of maxillary teeth (28–40) which is characteristic of Aristonectinae. Currently, the most parsimonious explanation concerning elasmosaurid evolutionary relationships, is that *Cardiocorax mukulu* represents an older lineage of elasmosaurids in the Maastrichtian.

## Introduction

Plesiosaurs are found on every continent and are represented by a fossil record that extends for over 130 million years through the Mesozoic [1–3]. Plesiosauria rapidly diversified in the Early Jurassic [4], and experienced a turnover event following the end of the Jurassic, with only three lineages crossing into the Cretaceous [1]. The lineage Xenopsaria Benson and Druckenmiller, 2014 [1] extended into the Cretaceous, and includes the two clades of plesiosauroids, Elasmosauridae Cope, 1869 [5] and Leptocleidia Ketchum and Benson, 2010 [6]. Elasmosauridae is notable for its extreme neck elongation, the function of which is not entirely clear [7–9].

The earliest unambiguous representatives of this clade are from the Hauterivian of Europe, and by the Aptian-Albian Elasmosauridae had achieved a global distribution [3, 10–21]. At least two clades of Elasmosauridae survived into the Campanian. In the Southern Hemisphere, Weddellonectia O’Gorman and Coria, 2017 [22], which includes Aristonectinae O’Keefe and Street, 2009 [23] (*sensu* Otero, Soto-Acuña, and Rubilar-Rogers, 2012) [24], and Elasmosaurinae Cope, 1869 [5] (= Styxosaurinae Otero, 2016) [7] in the Northern Hemisphere, with possible early branching elasmosaurid lineages surviving into the Maastrichtian [3]. While elasmosaurid remains are found globally, their fossil record in Africa is sparse, with the exception of the Angolan record [25–27].

Currently, six valid plesiosaurian taxa have been named and identified from the continent of Africa [26, 28–33]. From Angola, plesiosaur remains have been recovered from several coastal localities, notably Iembe in Bengo Province and Bentiaba in Namibe Province [25–27, 34–37]. Two distinct taxa have been identified from Bentiaba, *Cardiocorax mukulu* Araújo, Polcyn, Schulp, Mateus, Jacobs, Gonçalves, Morais, 2015 [26] and Aristonectinae gen. et sp. indet. [27]. The holotype of *Cardiocorax mukulu* is comprised of only postcranial elements and was diagnosed by autapomorphies of the cervical vertebrae and the pectoral girdle [26]. Araújo et al. (2015) [26] recovered *Cardiocorax mukulu* as the sister taxon of *Styxosaurus snowii* Williston, 1890 [38]. The new specimen of *Cardiocorax mukulu* (MGUAN PA278) described here includes the most complete skull of a plesiosaur known from sub-Saharan Africa, along with a mandible, vertebrae, and appendicular elements. The quality of the preservation is important in the context of providing novel data toward understanding elasmosaurid evolutionary history and elasmosaurid cranial anatomy where in most cases the skulls of recovered elasmosaurid specimens are missing or severely distorted, which obscures detailed anatomy and important characters within the palate and braincase [7, 13, 17, 39–42]. This study focuses on the anatomical description of MGUAN PA278, comparisons with other elasmosaurid taxa, and phylogenetic analyses of Plesiosauria to determine the evolutionary affinity of *Cardiocorax mukulu* within Elasmosauridae.

### Geologic setting

**Fig 1:**
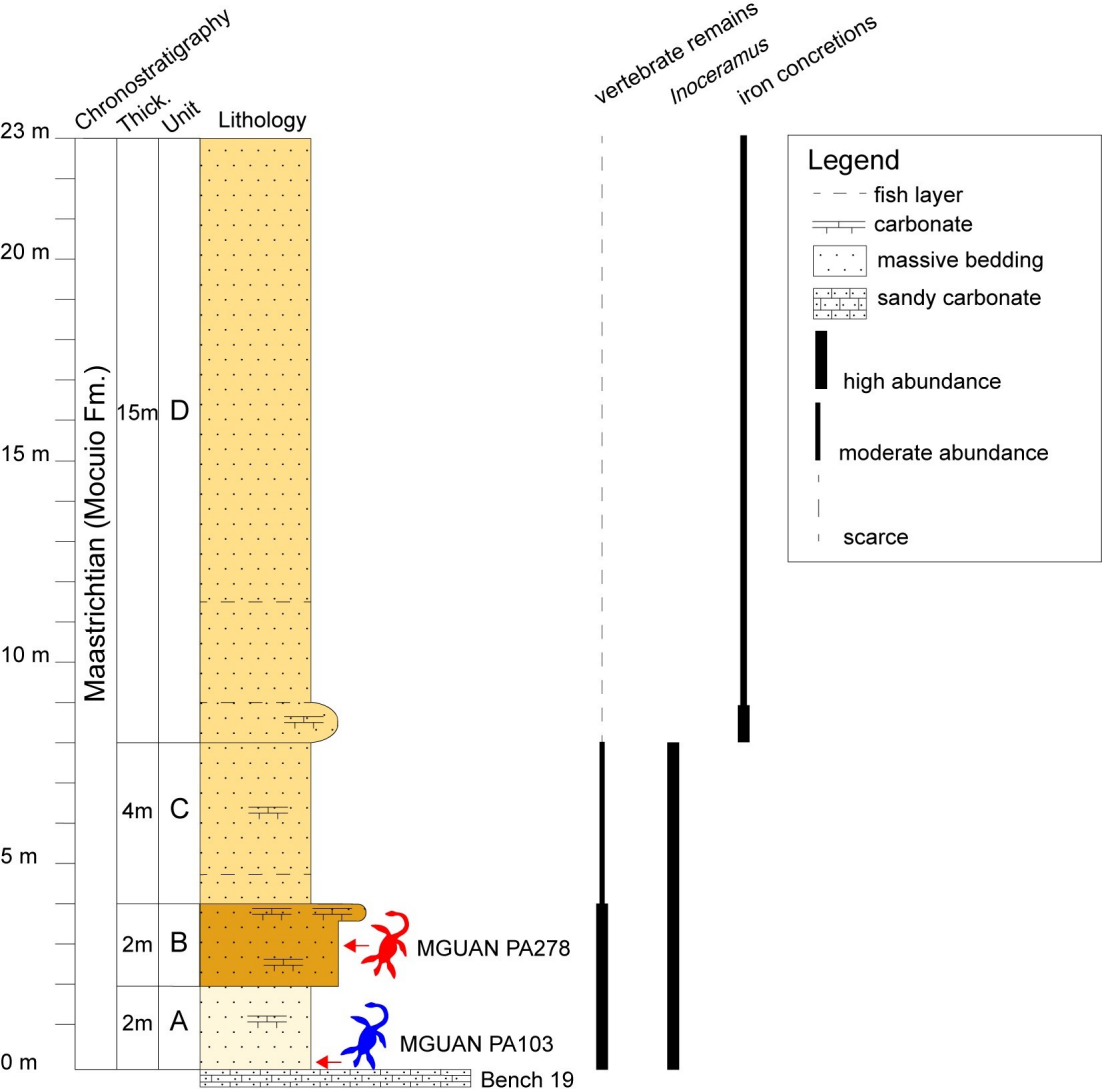
Stratigraphic section above Bench 19 near Bentiaba, Angola. The new plesiosaur specimen (MGUAN PA278) was recovered approximately three meters above the holotype of *Cardiocorax mukulu* (MGUAN PA103).

The age of the strata exposed at Bentiaba are based on magnetostratigraphy anchored by an 84.6 ± 1.5 Ma ^40^Ar/^39^Ar date on the Ombe basalt, which is overlain by a series of bench-forming sandstones [43, 44]. Vertebrate fossils are concentrated above Bench 19, in an immature feldspathic sandstone that falls within chron C32n.1n (71.64–71.40 Ma) [43, 44]. The dense concentration of fossils above Bench 19 along with the high taxonomic diversity of marine amniotes present at this site reflect the Cretaceous productivity of the shallow marine environment at Bentiaba, a likely result of the early Benguela upwelling system [44, 45]. The upper part of the Mocuio Formation above Bench 19, exposed at Bentiaba, is here informally divided into four unnamed units, A through D (Fig 1). The new specimen was recovered in unit B, a fine dark yellow sandstone approximately three meters above Bench 19 in the Mocuio Formation (Fig 1). Associated fauna in the immediate vicinity includes abundant large inoceramid bivalves, and unknown benthic organisms evidenced by vertical bioturbation, fishes (elasmobranchs and teleosts), sea turtles, and mosasauroids [25]. Plant remains were also recovered from the 2017 excavation in Bentiaba.

## Material and methods

### Institutional abbreviations

**AMNH**, American Museum of Natural History, New York City, New York, U.S.A.; **D**, Musée de Rhinopolis, Gannat, France; **DMNS**, Denver Museum of Nature and Science, Denver, Colorado, U.S.A.;**FCT/UNL**, Faculdade de Ciências e Tecnologia da Universidade Nova de Lisboa, Caparica, Portugal; **KUVP**, University of Kansas, Vertebrate Paleontology, Museum of Natural History, Lawrence, Kansas, U.S.A.; **LACM**, Natural History Museum of Los Angeles County, Los Angeles, California, U.S.A.; **MGUAN**, Museu de Geologia da Universidade Agostinho Neto, Luanda, Angola; **SMNK**, Staatliches Museum für Naturkunde, Karlsruhe, Germany; **SMU SMP**, Southern Methodist University Shuler Museum of Paleontology, Dallas, Texas, U.S.A.; **UCMP**, University of California Museum of Paleontology, Berkeley, California, U.S.A.; **UNSM**, University of Nebraska State Museum, Lincoln, Nebraska, U.S.A.

### Anatomical abbreviations

**aa**., anterior ampulla; **aac**., atlas-axis complex; **an**., angular; **ar**., articular rim; **art**., articular; **asc**., anterior semicircular canal; **atic**., atlas intercentrum; **atna**., atlas neural arch; **atr**., atlas rib; **axc**., axis centrum; **axna**., axis neural arch; **axr**., axis rib; **bc**., braincase; **bo**., basioccipital; **bpp**., basipterygoid process; **bs**., basisphenoid; **c**., centrum; **cc**., crus communis; **cor**., coronoid; **ct**., crista trabecularis; **cv**., cervical vertebra; **d**., dentary; **ect**., ectopterygoid; **en**., external naris; **epi**., epipterygoid; **exo**., exoccipital; **exo-op**., exoccipital-opisthotic; **f**., frontal; **fcc**., foramen for cerebral carotid; **fm**., foramina; **fo**., fenestra ovalis; **hr**., hypophyseal ridge; **hsc**., horizontal semicircular canal; **j.**, jugal; **lag**., lagena **llr**., lateral longitudinal ridge; **ms**., medial sinus; **mx**., maxilla; **na**., neural arch; **nc**., neural canal; **ns**., neural spine; **p**., parietal; **pa**., posterior ampulla; **pal**., palatine; **par**., paroccipital process; **pbs**., parabasisphenoid; **pm**., premaxilla; **po**., postorbital; **poz**., postzygapophysis; **pr**., prootic; **pra**., prearticular; **prf**., prefrontal; **prz**., prezygapophysis; **ps**., parasphenoid; **psc**., posterior semicircular canal; **pt**., pterygoid; **q**., quadrate; **qpt**. quadrate process of pterygoid; **r**., rib; **sa**., surangular; **so**., supraoccipital; **spl**., splenial; **sq**., squamosal; **st**., sella turcica; **v**., vomer; **vk**., ventral keel; **vn**., ventral notch; **vs**., vestibule.

### Material

**Fig 2:**
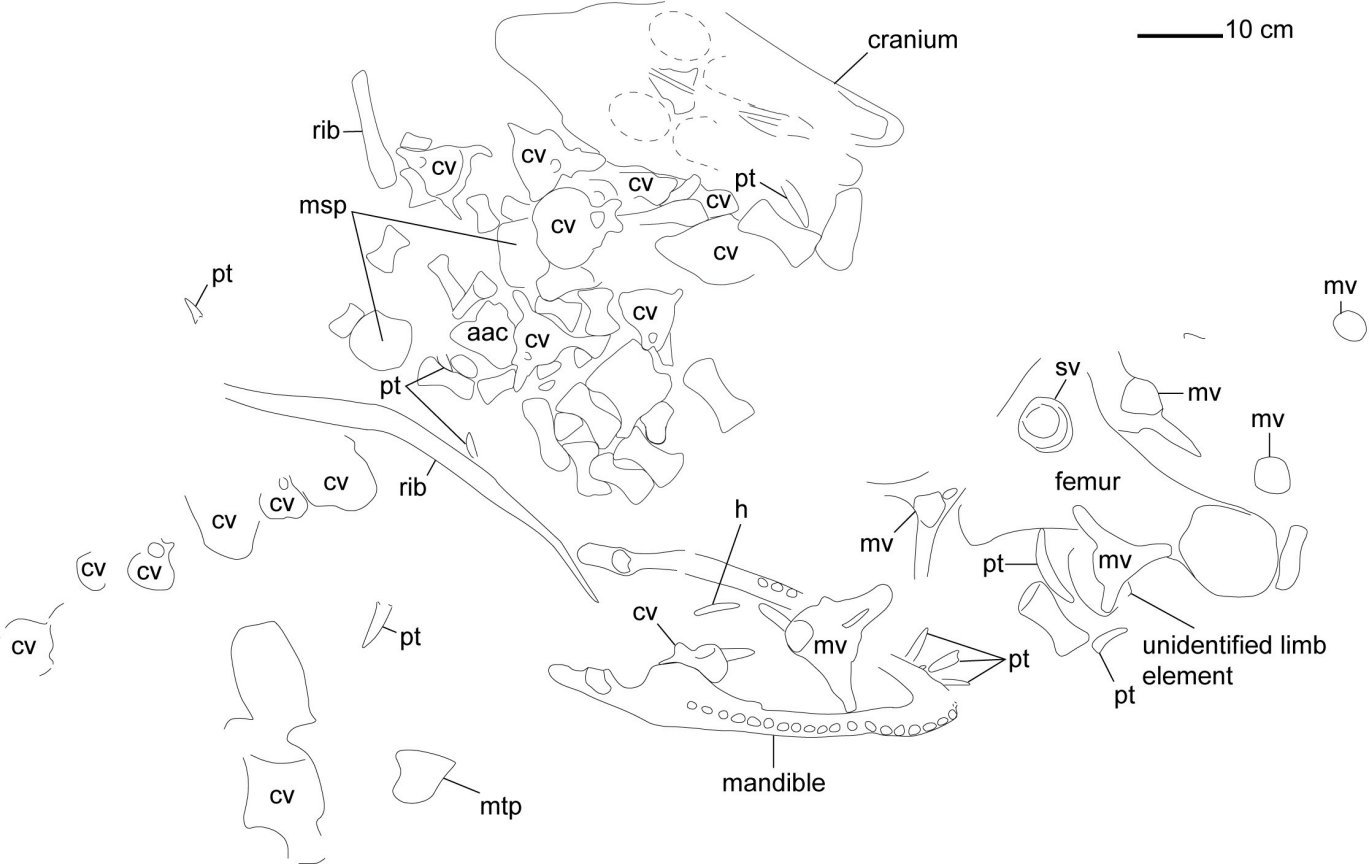
Excavation map of *Cardiocorax mukulu* (MGUAN PA278). Abbreviations: **aac**., atlas-axis complex; **cv**., cervical vertebra; **h**., hyoid bone; **msp**., mesopodial; **mtp**., metapodial; **mv**., mosasaur vertebra; **pt**., plesiosaur tooth; **sv**., shark vertebra. The spatial relationships and scaling of the elements are approximate.

The specimen (MGUAN PA278) includes an essentially complete skull, 12 displaced but associated teeth outside the skull, two hyoid bones, an atlas-axis complex, 17 postaxial cervical vertebrae, two partial ribs, a femur, two mesopodial elements, one metapodial element, and 32 phalanges. One unidentified limb element remains beneath the femur in the field jacket. None of the elements found in the field were articulated aside for some of the phalanges (Fig 2).

### Methods

Field research was permitted by the University of Agostinho Neto (Luanda, Angola) and the temporary exportation of fossil material was permitted by the Angolan Ministry of Geology and Mines (Ministério da Geologia e Minas). The plaster jackets containing MGUAN PA278 were opened and partially mechanically prepared at the Universidade Nova de Lisboa (FCT/UNL) in the Prof. Miguel Telles Antunes Laboratório de MacroPaleontologia using airscribe and hand tools. Using mechanical preparation, matrix was removed from the dorsal surface of the snout, skull roof, the lateral surface of the right temporal bar, part of the left maxilla and jugal, the squamosals, quadrates, and the occiput. Due to the delicate nature of the specimen, further preparation was halted and the skull jacket was CT-scanned at the University of Texas High-Resolution X-ray CT Facility (UTCT), using the following parameters: NSI scanner, GE Small Spot source, 300 kV, 0.75mA, 4 brass filter, Perkin Elmer detector, 0.25 pF gain, 2 fps, 1 x 1 binning, no flip, source to object 670.0 mm, source to detector 1086.0 mm, helical non-continuous CT scan, vertical extent 590.9 mm, pitch 98.467 mm, 6 revolutions, 3 sets, helical sigma 0.0, 4 frames averaged, 0 skip frames, 18005 projections, 5 gain calibrations, 15 mm calibration phantom, data range [-0.5, 5.5] (grayscale adjusted from NSI defaults), beam-hardening correction = 0.45; voxel size = 141.9 μm; total slices = 3449.

The 16-bit TIFF images were used for segmentation of the cranial bones and the endosseous labyrinth using AMIRA 6.3 [46], at Southern Methodist University (SMU). The surface model of the segmented skull was processed in MeshLab [47] using the Laplacian filter at 10 smoothing steps reduced from 3,367,985 polygons to 1,000,000 polygons using quadric decimation and an additional Laplacian filter at 3 smoothing steps. The braincase was processed using Laplacian filter at 10 smoothing steps and reduced from 1,578,782 polygons to 500,000 polygons using quadratic decimation. The endosseous labyrinth was processed with a Laplacian filter at 10 smoothing steps with no polygon reduction. Orthogonal views of the cranium and semicircular canals were rendered in LightWave 3D 2018 [48].

Collection visits by M.M. allowed comparison of MGUAN PA278 with the holotypes of *Libonectes morgani* Welles, 1949 [49], *Callawayasaurus colombiensis* Welles, 1962 [10], *Styxosaurus snowii*, as well as the holotype and referred specimens of *Cardiocorax mukulu*, and UNSM 50132, referred to *Thalassomedon haningtoni* Welles, 1943 [13, 39, 50]. Interpretive figures of *Styxosaurus snowii* and *Thalassomedon haningtoni* are provided in the S1 File. Additional comparisons were based on the literature.

We performed six phylogenetic analyses using TNT v.1.5 [51, 52] to test the evolutionary relationship of *Cardiocorax mukulu* within Plesiosauria. We used the matrix of Fischer et al. (2020) [12] (130 taxa; 283 characters), which builds upon the matrix of O’Gorman (2020) [3], which in turn is an iteration of the parent matrix by Benson and Druckenmiller (2014) [1]. The matrix of Fischer et al. (2020) [12] was modified by including character scores for *Libonectes morgani* (SMU SMP 69120 and SMNK-PAL 3978), *Styxosaurus snowii* (KUVP 1301) and *Thalassomedon haningtoni* (DMNS 1588 and UNSM 50132) from the Sachs et al. (2021) [13] matrix because these specimens were not personally revised by O’Gorman (2020) [3], other than DMNS 1588, whereas Sachs and Kear. (2017) [53], Sachs et al. (2018) [42], and Sachs et al. (2021) [13] visited these specimens and revised the scores. Character scores for *Libonectes morgani*, *Styxosaurus snowii*, and *Thalassomedon haningtoni* were further revised based on our first-hand interpretations of SMU SMP 69120, KUVP 1301, and UNSM 50132 and a review of the literature describing these specimens [39, 41, 42, 49, 53]. We also provide character score modifications to *Callawayasaurus colombiensis* based on our first-hand interpretation of the skull of UCMP 38349. These modifications were done in the hope of resolving the unstable interrelationships of Elasmosauridae. See the S1 File for a list of our character modifications for *Libonectes morgani*, *Styxosaurus snowii*, *Thalassomedon haningtoni*, and *Callawayasaurus colombiensis* and arguments for each character change. For our phylogenetic analyses, we ran a heuristic search of Plesiosauria, a ‘New Technology’ search without implied weighting, and two ‘New Technology’ searches incorporating implied weighting. Two additional ‘New Technology’ searches incorporating implied weighting were run with the recent matrix of Sachs et al. (2021) [13]. Our character scores for *Cardiocorax mukulu* were modified from the Fischer et al. (2020) [12] matrix to run in the Sachs et al. (2021) [13] matrix by changing character 139 from state 2 to state 3 based on the modification of character 139 by Madzia and Cau (2020) [54] and inputting only characters 1–270. The details of each analysis are covered in the phylogeny section of this work. Bootstrap and Bremer indices were calculated for the heuristic search of Plesiosauria and the ‘New Technology’ search without implied weighting. Tree statistics and Bremer indices were calculated using the *stats*.*run* and *bremer*.*run* scripts supplied by TNT [52]. The command ‘keep 10’ was used prior to running the *stats*.*run* script, as the large number of generated trees kept the statistics script from operating. The implied weighting analyses were subject to symmetric resampling, using the same criteria of Sachs et al. (2021) [13], with the ‘Traditional search’ option and change probability at its default of 33 and 1,000 replicates, with the output set to (GC). Character scoring for *Cardiocorax mukulu* is based on the holotype (MGUAN PA103) and two specimens referred to *Cardiocorax mukulu* (MGUAN PA270 and MGUAN PA278) [26]. Nexus and TNT files were generated using Mesquite v.3.61 (Build 927) [55].

127/283 characters were scored for MGUAN PA278, and 177/283 characters were scored for the *Cardiocorax mukulu* composite (MGUAN PA103, MGUAN PA270, and MGUAN PA278), as opposed to 33/283 characters scored for *Cardiocorax mukulu* by O’Gorman (2020) [3] and used by Fischer et al. (2020) [12]. In total, 144 new character scores were added in this study to *Cardiocorax mukulu*. The holotype of *Cardiocorax mukulu* was rescored in-person by M.M. Compared to the character scores for *Cardiocorax mukulu* from O’Gorman (2020) [3], 37 character scores were changed, and 25 character scores were maintained from those of O’Gorman (2020) [3].

Measurements of the cranium, mandible, and vertebrae of MGUAN PA278 were taken from the CT data using Dragonfly 4.1 and Dragonfly 2020.1 Build 809 (Object Research Systems ORS Inc, Montreal, Canada, 2018; software available at http://www.theobjects.com/dragonfly) [56], Meshlab [47], and from the physical specimen using a digital caliper. Images of the CT data were taken using Dragonfly 2020.1 Build 809 software [56] to make the figures. The cervical vertebrae are ordered by comparing length, width, and height dimensions, as well as morphological differences; however, the precise position of the cervical vertebrae cannot be assessed due to disarticulation and potential loss. Thus, the anterior-most cervical vertebra is designated ‘anterior cervical vertebra a’, and the next cervical vertebra in the relative sequence is ‘anterior cervical vertebra b’ and so on. Vertebral measurements were taken using the methodology of Welles (1952) [57] where height and width are taken from the anterior articular facet of the centrum. As noted by Welles (1952) [57], height is measured as maximum height of the anterior face of the centrum, not taking into account the neural canal. Length is maximum length of the centrum, and we measured length using the lateral surface of the centra as well as the length of the centra along the ventral surface at mid-width [57].

### Taphonomy

**Fig 3:**
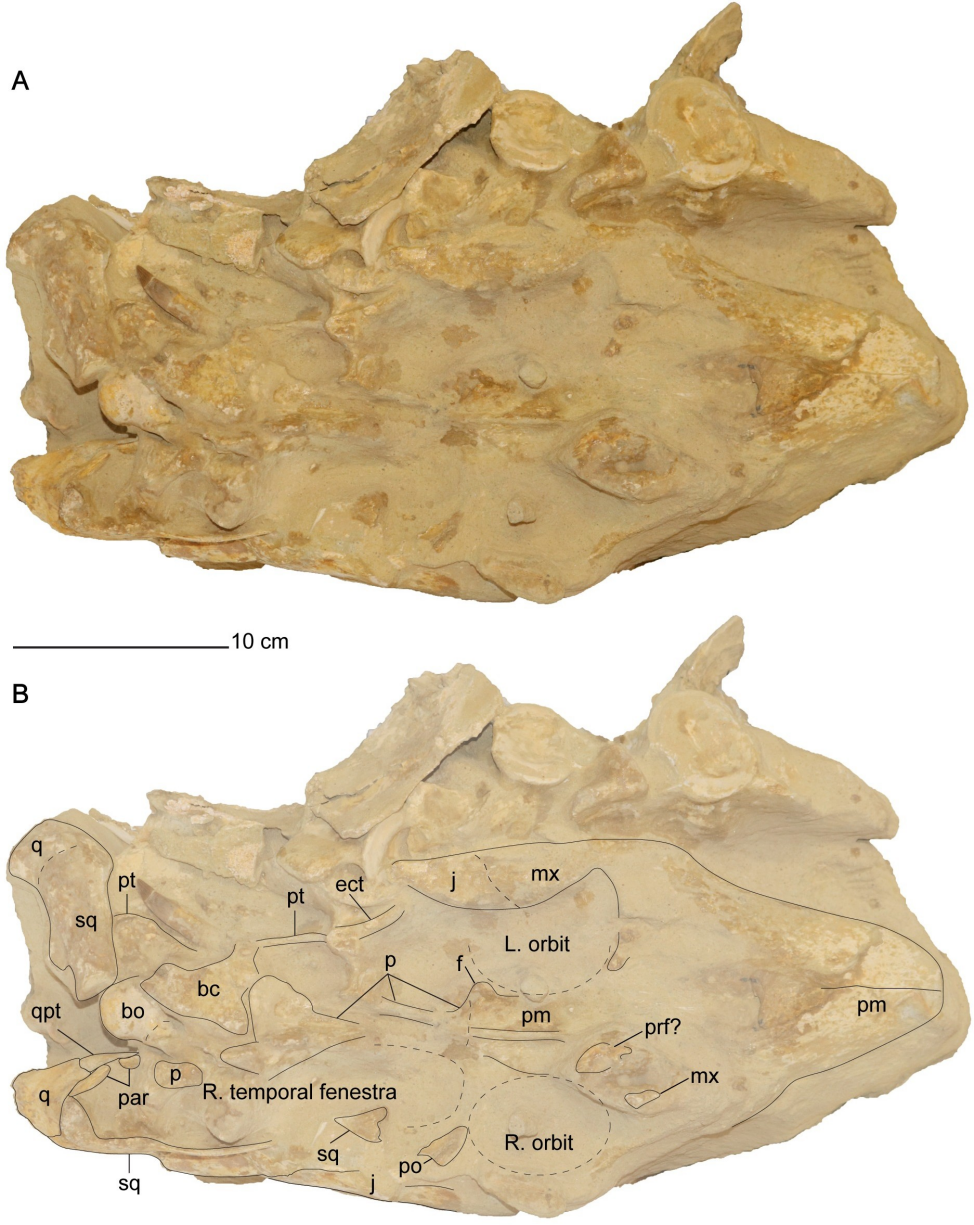
Cranium of *Cardiocorax mukulu* (MGUAN PA278) in dorsal view. A. dorsal view of cranium without labels. B. dorsal view with anatomical labels.

All elements of the new specimen were found in close proximity and extracted from the field in jackets. The skull was preserved in a layer of sandstone with only the right ramus of the mandible exposed to weathering processes. On the surface of the skull block, crushing and displacement of the skull is evident. A large crack in the cranium runs posteromedially from the anterior extent of the left premaxilla (Fig 3). All erupted teeth in the cranium are absent, with only two remaining in the mandible. Pre-burial alteration affected the skull roof posterior to the snout, as the surface of the bone is worn without having been exposed in the field. The left maxilla and the left jugal which contribute to the ventral border of the left orbit remain articulated but are displaced with the dorsal margin of the left maxilla and jugal facing medially and the ventral margin facing laterally. The dorsal ramus of the left maxilla is broken at the base, while the dorsal ramus of the right maxilla is not broken and remains oriented vertically as in life position. Two calcareous burrows are evident, one through each orbit indicating post-burial bioturbation by benthic organisms. Only a single possible bite mark is evident on one of the anterior cervical vertebrae. Although no substantial scavenging marks are apparent on the bones, *Squalicorax pristodontus* may have contributed to disarticulation and transport of the specimen, as shed teeth of this shark were recovered from the excavation site.

**Fig 4:**
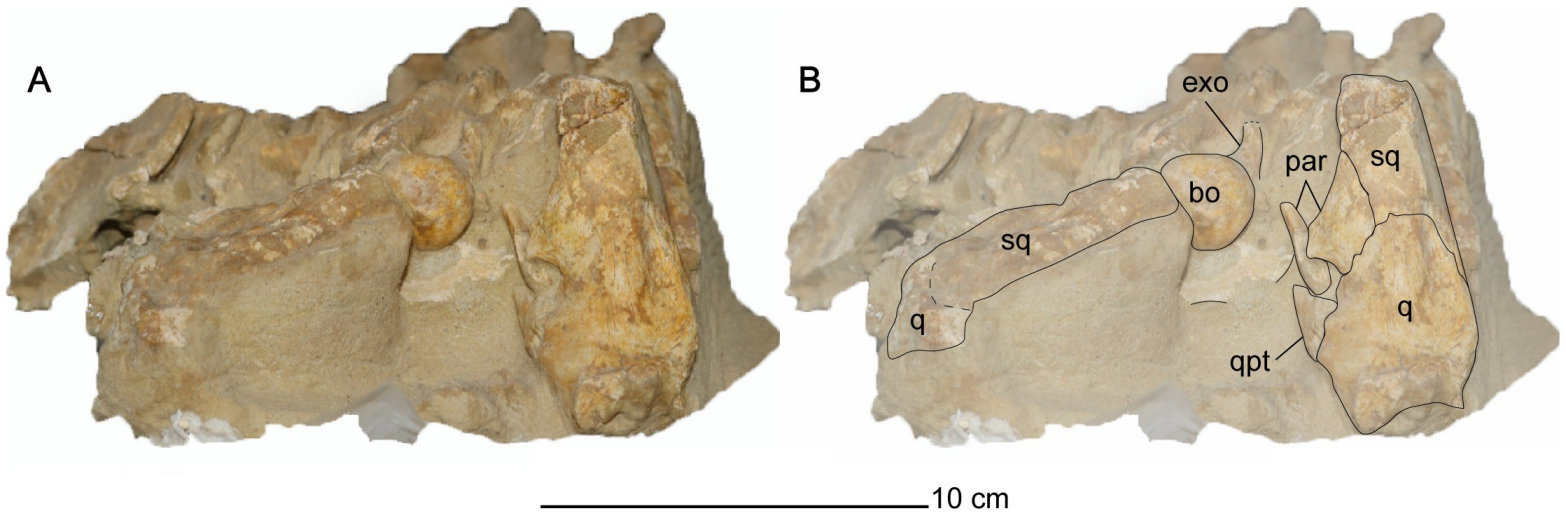
Exposed occiput of *Cardiocorax mukulu* (MGUAN PA278) cranium. A. Occiput without labels. B. Occiput elements interpreted.

The parietal and braincase elements are sheared to the right side of the cranium. The left quadrate and squamosal remain articulated to each other but are rotated so that the dorsal margin of the left squamosal faces medially and the condyles of the left quadrate face posterolaterally. The occiput and the right posterolateral portion of the skull comprising the right squamosal, quadrate, paroccipital process, and quadrate process of the pterygoid lack significant distortion and are one of the best-preserved areas of the skull with respect to three-dimensional articulation (Fig 4). One cervical vertebra exhibits shear-like plastic deformation. A thin calcareous residue covers all the observable cervical vertebrae.

### Taphonomic interpretation of CT data

The dorsomedial ridge of the left premaxilla is damaged but preserved on the right premaxilla. The complete outline of the external nares cannot be assessed due to compression of the skull and pre-burial erosion along the posterior extensions of the premaxillae, although their location is still identifiable by a smooth concavity on the maxilla anterior to the preorbital bar. Posterior to the premaxillae, the parietal is broken into five separate fragments along the dorsal midline of the skull.

With respect to the braincase, the parasphenoid and basisphenoid are tightly fused, although a narrow space separates the basisphenoid from the basioccipital. The prootics are slightly displaced from the basicranium. Shearing of the braincase is evident, as the left prootic is tilted medially, and the right prootic and exoccipital-opisthotic are tilted laterally to the right. The semicircular canals on the right side of the braincase are clearly visible between the prootic, exoccipital-opisthotic, and the supraoccipital. However, on the left-half of the braincase, the semicircular canals are not entirely preserved due to breakage or bioerosion. The slightly displaced supraoccipital is broken into three fragments. A thick calcareous encrustation is visible within the alveoli of the upper tooth row, and on the ventral surface of the basioccipital.

Pre-burial erosion on the skull, displaced teeth, burrowing, and the displacement of the cervical vertebrae in addition to elements from the hindlimb indicates prolonged exposure of the new specimen on the seafloor (Fig 2). The body lay on the sea bottom and was scattered but largely intact prior to burial. Subsequent sediment loading led to crushing, shearing, and breakage.

## Results

### Systematic paleontology

DIAPSIDA Osborn, 1903 [58]
SAUROPTERYGIA Owen, 1860 [59]
PLESIOSAURIA de Blainville, 1835 [60]
XENOPSARIA Benson and Druckenmiller, 2014 [1]
ELASMOSAURIDAE Cope, 1869 [5]
*Cardiocorax mukulu* Araújo, Polcyn, Schulp, Mateus, Jacobs, Gonçalves, and Morais, 2015 [26]

Holotype: MGUAN PA103: one anterior cervical vertebra, five posterior cervical vertebrae, one posterior cervical neural arch, one dorsal vertebra, a humerus, radius, radiale, ulna, a supernumerary element, both coracoids, left scapula, fragments of the right scapula, left clavicle, interclavicle, both pubes and ischia, an ilium, fragments of dorsal ribs, and phalanges.

Referred specimens: MGUAN PA278: A nearly complete cranium and mandible, 12 associated teeth, hyoid bones, an atlas-axis complex, 17 postaxial cervical vertebrae, two partial dorsal ribs, a femur, two mesopodial elements, one metapodial, a possible epipodial or mesopodial, and 32 phalanges.

MGUAN PA270: A nearly complete pelvic girdle (missing a pubis and an ilium) and a nearly complete hindlimb (Fig 11 of Mateus et al. (2012) [25] described in Araújo et al. (2015) [26]).

Horizon: Mocuio Formation. Holotype of *Cardiocorax mukulu* (MGUAN PA103) was recovered just above Bench 19 [43, 44] and the new specimen (MGUAN PA278) was recovered approximately three meters above Bench 19 and approximately 250 meters northeast of the holotype.

Age: Early Maastrichtian (71.64–71.40 Ma) [43, 44]
Locality: Bentiaba, Moçâmedes Municipality, Namibe Province, Angola

Emended diagnosis:

*Cardiocorax mukulu* can be differentiated from all other elasmosaurids based on the following combination of characters: A dorsal ramus of the maxilla forms the majority of the anterior margin of the orbit; five premaxillary alveoli, seventeen maxillary alveoli, and at least twenty dentary alveoli; parietal extends to postorbital bar; no medial contact between the pterygoids beneath the basioccipital; middle and posterior cervical neural spines are approximately as broad anteroposteriorly as the centra and exhibit a sinusoidal anterior margin; clavicles contact each other along entire medial margin; coracoids form laterally extensive cornua that extend lateral to the pectoral glenoid and form a symphysis posterior to the intercoracoid fenestra; presence of a pelvic bar; anteroposterior length to minimum mediolateral width of the pubis is elongated (greater than 1.3). One autapomorphy is evident in *Cardicorax mukulu* relative to other elasmosaurids: clavicular ventral area nearly as broad as scapular ventral area [26].

### Osteological description

#### Cranial skeleton

**Fig 5:**
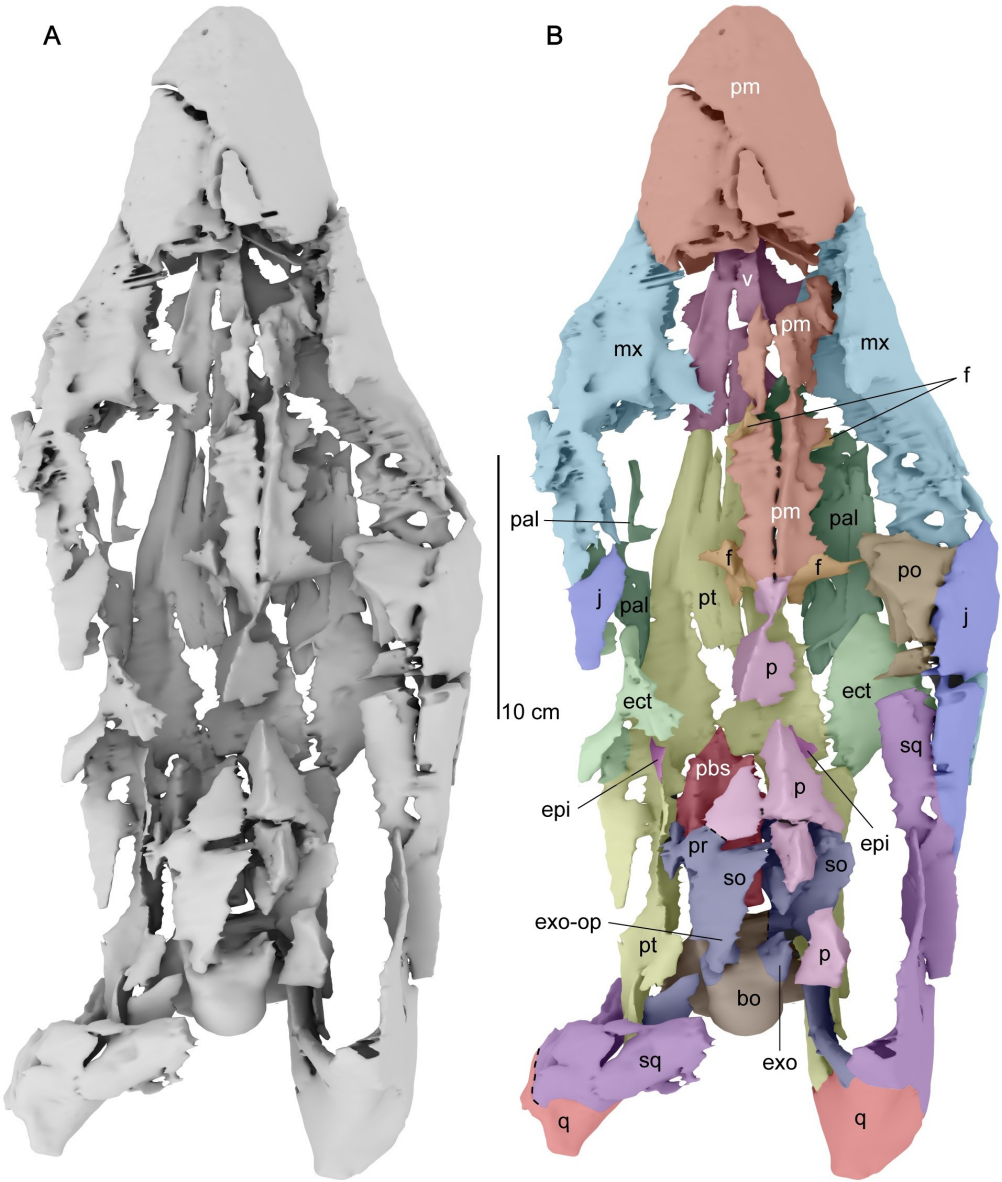
Three-dimensional digital model of the cranium of *Cardiocorax mukulu* (MGUAN PA278) in dorsal view. A. uninterpreted surface model. B. separate cranial elements labeled. Dashed lines indicate approximate sutural contacts.

*Premaxilla*. The snout forms a rounded and blunt tip with a clear median suture between both premaxillae. The suture between the premaxillae is straight and visible in dorsal view of the skull (Fig 3). The snout is almost 40% of the length of the cranium (Table 1). A dense array of foramina is apparent across the dorsal and lateral surface of the premaxilla. A dorsomedial ridge on the dorsal surface of the premaxilla becomes apparent posterior to the third premaxillary alveolus until the fifth premaxillary alveolus. Posterior to the fifth premaxillary alveolus, the premaxillae are too worn to determine if the dorsomedial ridge continues or if a premaxillary boss is present (Fig 5) as in *Styxosaurus snowii* [42], *Styxosaurus browni* Welles, 1943 [7, 39], and *Thalassomedon haningtoni* [13]. The suture between the premaxillae divides the dorsomedial process. Narrow posterior extensions of the premaxillae along the dorsal midline of the skull form the dorsomedial margin of the external nares. The suture between the premaxilla and the maxilla arises behind the fifth alveolus of the premaxilla and curves posteromedially to reach the anterior margin of the external naris.

**Table 1:** Cranial measurements of *Cardiocorax mukulu* (MGUAN PA278).

Length of cranium from tip of premaxillae to occipital condyle	39.0 cm
Length of cranium from tip of premaxillae to right quadrate (better preserved quadrate)	44.6 cm
Antorbital length of cranium (anterior margin of orbit to tip of premaxillae)	14.1 cm
Beak index (Welles, 1952) [57]	36.2%

Posterior extensions of the premaxillae overlap the frontals between the orbits. Within the intraorbital region the dorsomedial ridge of the premaxilla is low and mediolaterally narrow. The ventral margin of the premaxilla and the medial margin of the frontal forms the olfactory sulcus that extends between the orbits. The posterior termination of the premaxilla is identified by the disappearance of the dorsomedial process at the sutural contact with the parietal (Fig 5). Contact between the premaxilla and the parietal is located medial to the postorbital bar and forms an interdigitating suture. The premaxillary-parietal suture of MGUAN PA278 at the level of the postorbital bar is shared with *Zarafasaura oceanis* Vincent, Bardet, Pereda Suberbiola, Bouya, Amaghzaz, and Meslouh, 2011 [31], and *Nakonanectes bradti* Serratos, Druckenmiller, and Benson, 2017 [41] but distinct from *Libonectes morgani* [61] (S5 Fig), *Hydrotherosaurus alexandrae* Welles, 1943 [3, 39], *Styxosaurus snowii* [42] (S4 Fig), *Terminonatator ponteixensis* Sato, 2003 [40], and *Thalassomedon haningtoni* (S2 Fig) which exhibit a premaxillary-parietal suture located at or near the midpoint of the orbits along the skull roof.

**Fig 6:**
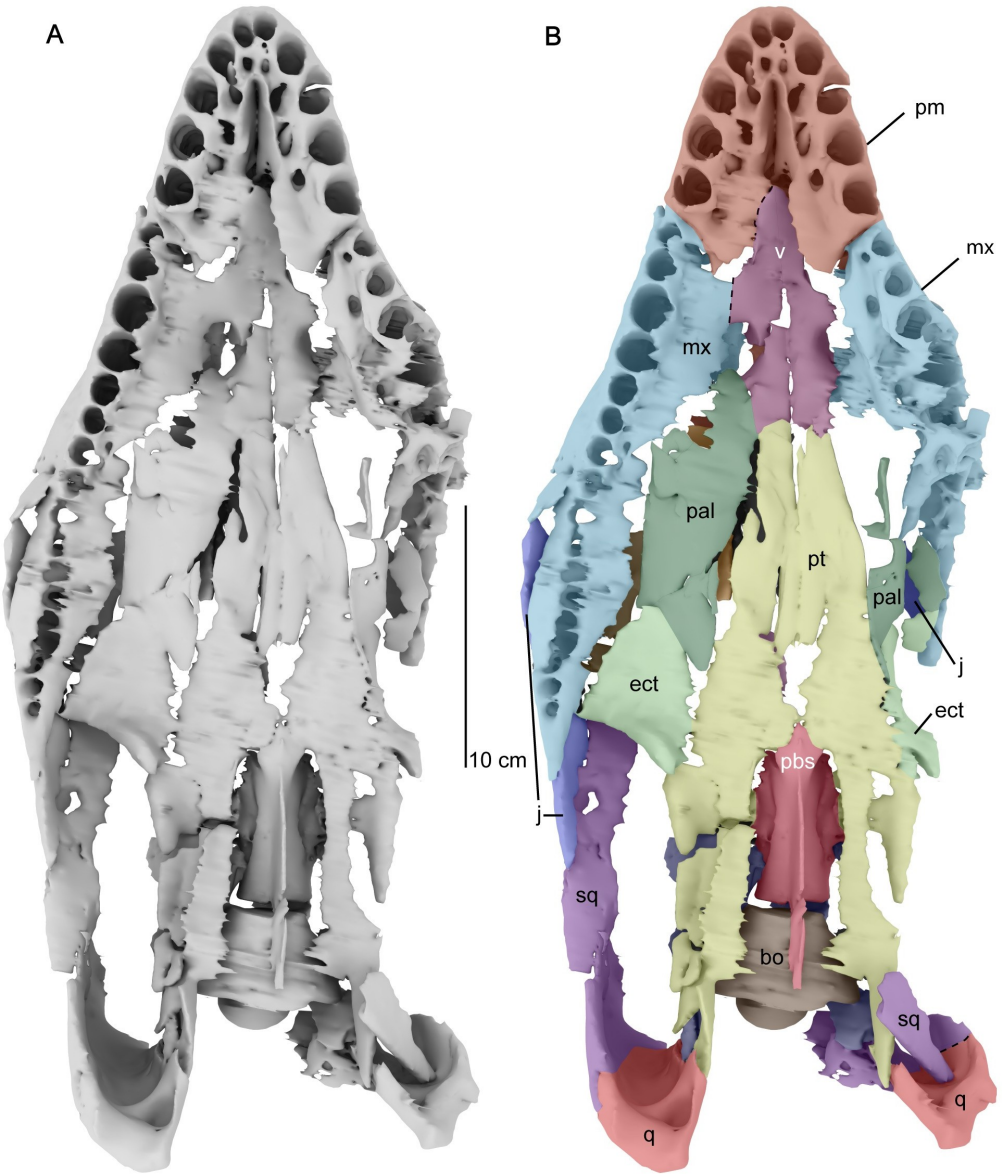
Three-dimensional digital model of the cranium of *Cardiocorax mukulu* (MGUAN PA278) in ventral view. A. uninterpreted surface model. B. separate cranial elements labeled. Dashed lines indicate approximate sutural contacts.

Each premaxilla of MGUAN PA278 has a lateral tooth count of five, as indicated by the number of premaxillary alveoli (Fig 6). The lateral tooth count in MGUAN PA278 is the same as *Styxosaurus snowii* [42], *Libonectes morgani* [61], *Tuarangisaurus keyesi* Wiffen and Moisley, 1986 [62, 63], *Lagenanectes richterae* Sachs, Hornung, and Kear, 2017 [11], *Leivanectes bernandoi* Páramo-Fonseca, O’Gorman, Gasparini, Padilla, and Parra Ruge, 2019 [20], and *Callawayasaurus colombiensis* [10], but differs from *Thalassomedon haningtoni* [13] (4 premaxillary teeth), *Elasmosaurus platyurus* Cope, 1868 [64, 65] (6 premaxillary alveoli), *Eromangasaurus australis* Sachs, 2005 [15, 17, 21] (3–4 premaxillary alveoli), and the aristonectines *Kaiwhekea katiki* Cruickshank and Fordyce, 2002 [53, 66] (7 premaxillary alveoli), *Morturneria seymourensis* Chatterjee and Smalls, 1989 [67, 68] (8–9 premaxillary teeth) and *Aristonectes parvidens* Cabrera, 1941 [53, 69, 70] (~13 premaxillary alveoli). Premaxillary alveoli become sequentially larger posteriorly. Replacement teeth are situated medial to the alveoli. Four replacement teeth remain within the premaxillae, but no erupted teeth remain. Teeth at the anterior end of the premaxillae are more procumbent than those more posteriorly located. The premaxilla contributes slightly to the palate lateral to the anterior projection of the vomer (Fig 6). A narrow opening between the third and fourth tooth position of the premaxillae is identified as the vomeronasal fenestra, although O’Gorman et al. (2017) [63] questions this homology.

**Fig 7:**
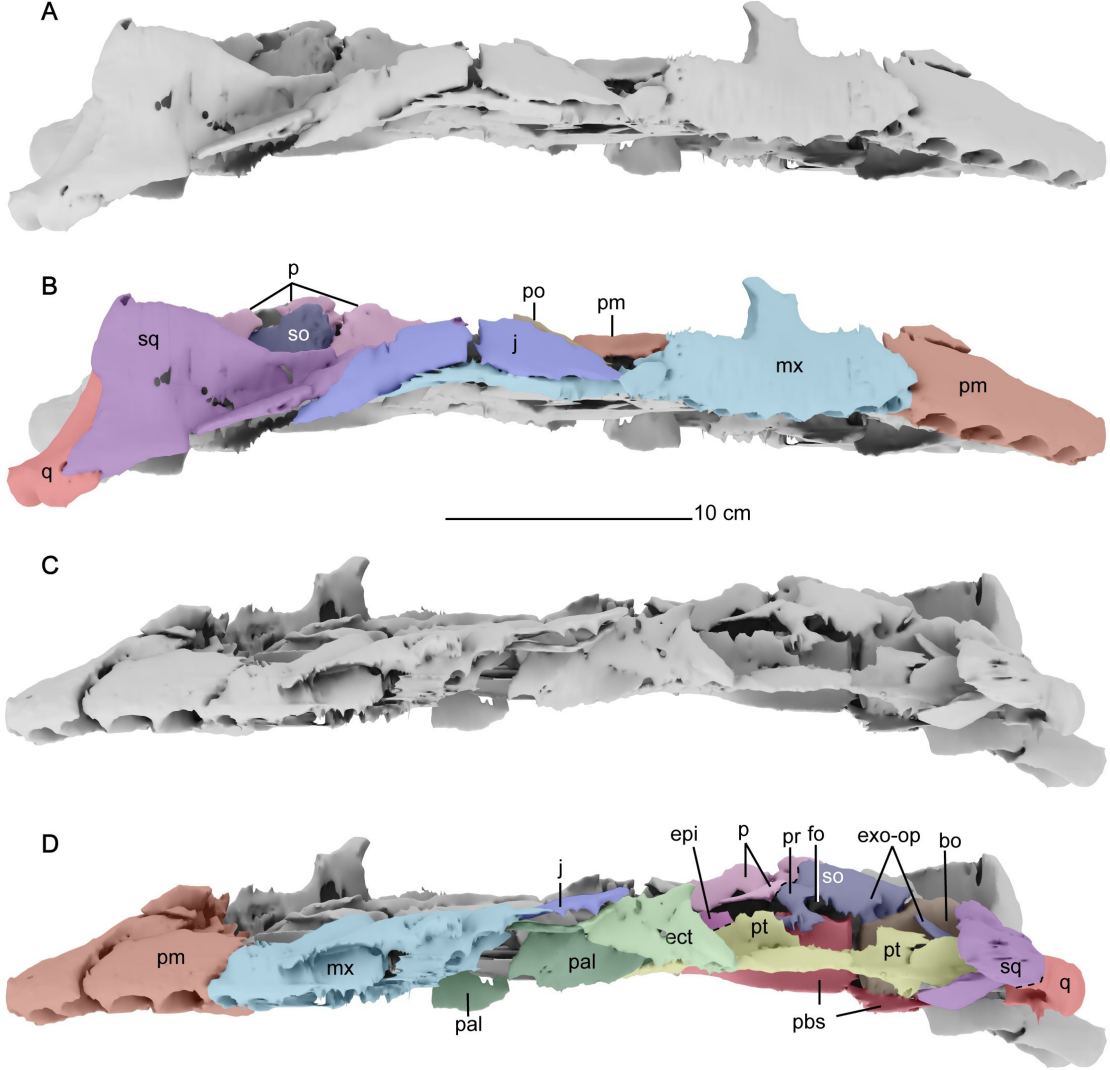
Lateral views of the cranium of *Cardiocorax mukulu* (MGUAN PA278). A-B. right lateral view. C-D. left lateral view. Dashed lines indicate approximate sutural contacts.

*Maxilla*. The maxilla contributes to a substantial portion of the anterior margin of the orbit and the orbitonasal bar via a dorsal ramus (Fig 7). This dorsal ramus of the maxilla is also present in *Libonectes morgani* [53, 61], *Nakonanectes bradti* [41], *Tuarangisaurus keyesi* [63], *Kaiwhekea katiki* [66], and *Eromangasaurus australis* [15, 17, 21] but modestly in *Thalassomedon haningtoni* [13] (S2 Fig), and completely absent in *Styxosaurus snowii* [42] (S4 Fig). Anterior to the dorsal ramus, the maxilla forms a smoothly concave surface for the ventral margin of the external naris. Posterior to the orbitonasal bar the maxilla contributes to the ventral margin of the orbit by forming a convex ventral border. Ventral to the orbit, the maxilla sutures with the jugal and divides the convex ventral border of the orbit. The convex margin formed by the maxilla and jugal provides the orbit with a reniform shape, a common characteristic of elasmosaurid plesiosaurians [1, 6, 13, 42, 53]. The maxilla tapers posteriorly along the ventral margin of the temporal bar. Contact between the maxilla and the squamosal is inhibited by the jugal. The maxilla extends medially to contribute to the lateral margin of the internal naris (Fig 6).

In palatal view, the maxillary alveoli are more circular in outline and less elliptical than the premaxillary teeth. In the right-half of the skull, maxillary alveoli one through six are sub-vertical in orientation, and maxillary alveoli seven through seventeen are vertical. The third maxillary alveolus (4.9 cm deep) in the right orbitonasal bar is the largest and has the greatest root accommodation for a tooth. The length and width of the maxillary alveoli in palatal view and their tooth depth decreases posteriorly. Seventeen alveoli are discernable in the maxilla. A count of 17 maxillary alveoli in MGUAN PA278 is distinguishable from the high maxillary alveoli counts of aristonectine elasmosaurids (more than 50 maxillary teeth in *Aristonectes parvidens* and 36 in *Kaiwhekea katiki*) [70], *Styxosaurus snowii* (at least 15 maxillary teeth) (S1 Fig), *Libonectes morgani* (13–14 maxillary teeth) [61], *Tuarangisaurus keyesi* (15 maxillary alveoli) [63], and *Nakonanectes bradti* (14 maxillary teeth) [41]. The upper tooth row on the right side of the skull terminates at approximately the midpoint of the supratemporal fenestra.

**Fig 8:**
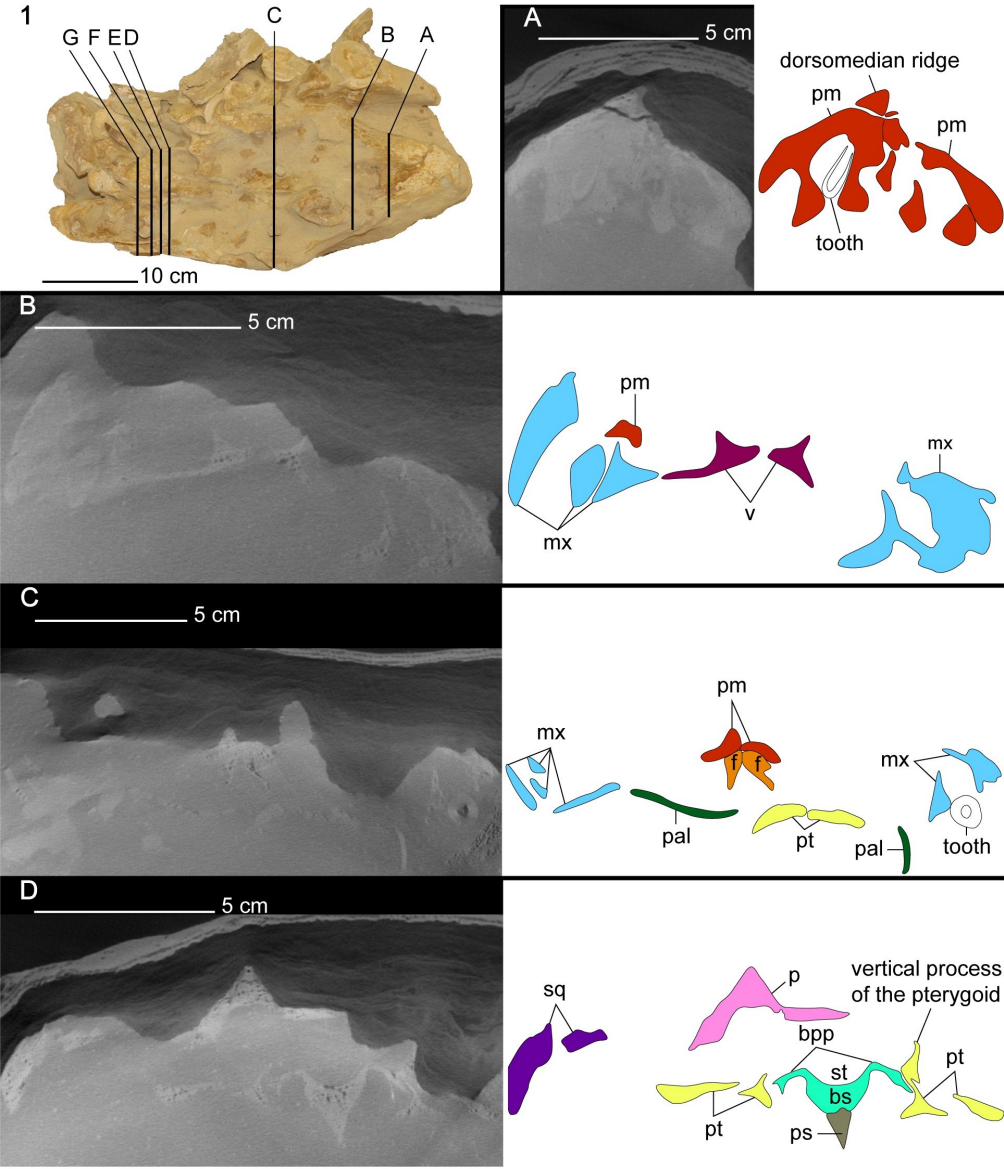
CT cross-section images of the cranium of *Cardiocorax mukulu* (MGUAN PA278). A. Transversal section through the third pair of premaxillary alveoli. B. anterior margin of the palate. C. Section of intraorbital region of the cranium. D. Section through the anterior margin of the sella turcica.

**Fig 9:**
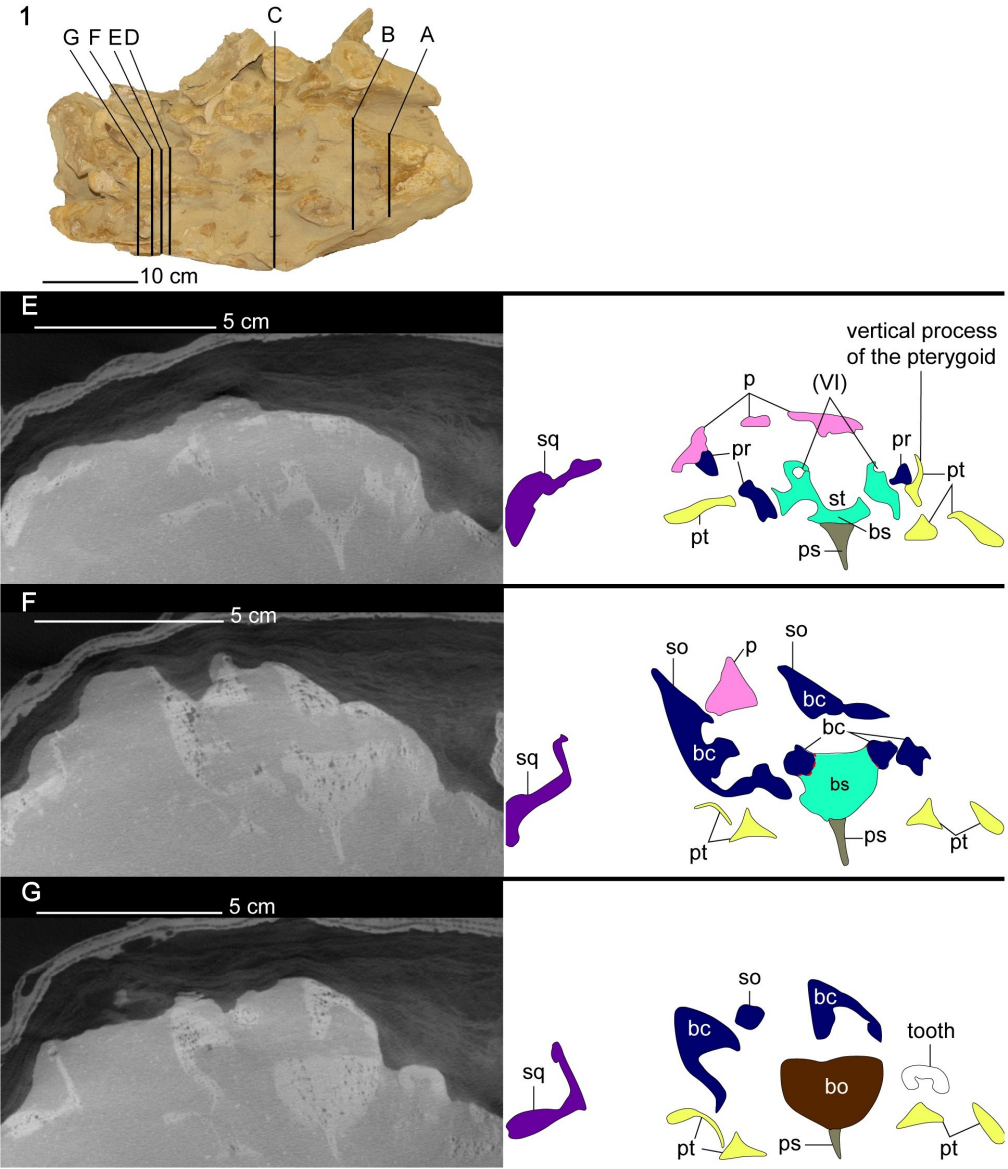
CT cross-section images of the cranium of *Cardiocorax mukulu* (MGUAN PA278). E. Section through the sella turcica exposing the abducens canal. F. Section through the mid-section of the braincase. G. Section through the posterior section of the braincase.

*Frontal*. Ventral to the posterior process of the premaxilla, the frontal forms the dorsomedial border of the orbit. The frontal is elongated, contributing to the postorbital bar, and the dorsal margin of the orbitonasal bar where it separates the external naris from the orbit. The frontal is triangular in cross-section (Fig 8C). The ventral extension of the frontal increases in depth posteriorly along the dorsomedial margin of the orbits. Posterior to the frontal, the skull roof is formed by the parietal (Fig 9). A frontal foramen, identified in polycotylid plesiosaurians [28, 61] and the elasmosaurid *Tuarangisaurus keyesi* [63], is not present in MGUAN PA278.

*Prefrontal*. A loose triangular fragment of bone located posterior to the right external naris and medial to the right dorsal ramus of the maxilla in MGUAN PA278 is tentatively referred to as the prefrontal (Fig 3). This prefrontal would have contributed to the anteromedial margin of the orbit.

**Fig 10:**
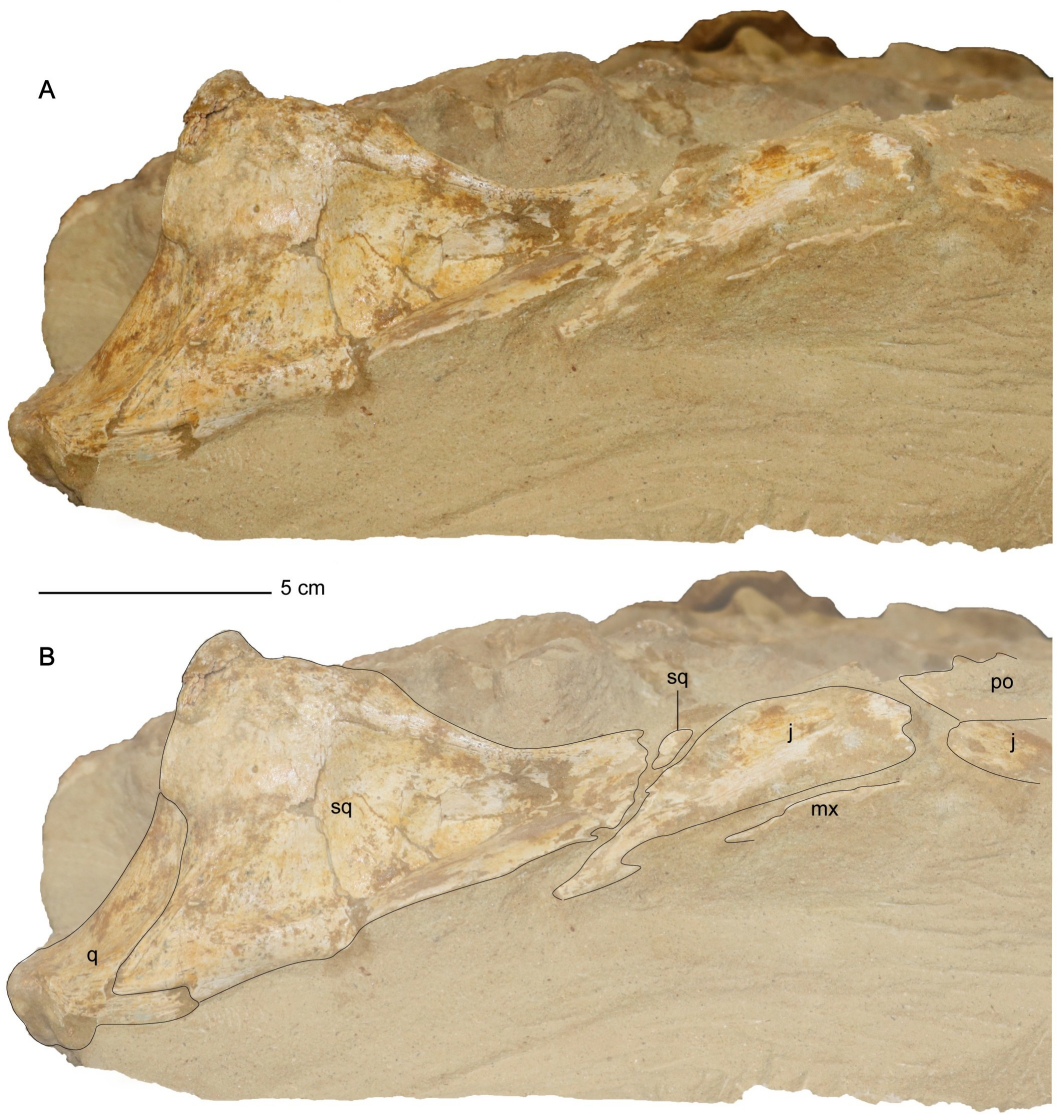
*Cardiocorax mukulu* (MGUAN PA278) with exposed right temporal bar. A. Right temporal bar. B. Borders of elements in right temporal bar labeled.

*Jugal*. The jugal is an elongated element that contributes to the posteroventral margin of the orbit, bordered ventrally by the maxilla and dorsally by the postorbital. The postorbital restricts the jugal from the dorsal margin of the temporal bar. The suture between the jugal and the squamosal forms a sharp interdigitating pattern and is inclined anterodorsally-posteroventrally (Fig 10). The jugal separates the maxilla from the squamosal as it does in almost all elasmosaurid plesiosaurians, except in *Libonectes morgani* [53], and *Nakonanectes bradti* [41].

*Postorbital*. The postorbital is a triangular element that forms the posterior border of the orbit and the anterior margin of the temporal fenestra. It has a posterior process forming part of the dorsal margin of the temporal bar posterior to the orbit. The postorbital contacts the jugal dorsally and extends posteriorly along the dorsal margin of the temporal bar where it contacts the anterior ramus of the squamosal. The postorbital terminates before the mid-point of the temporal fenestra (Fig 5). The postorbital would have contributed to a substantial portion of the posterior margin of the orbit (Fig 5). This condition is similar to *Libonectes morgani* [53], *Callawayasaurus colombiensis* [10], *Hydrotherosaurus alexandrae* [39], *Zarafasaura oceanis* [31], *Nakonanectes bradti* [41], and *Styxosaurus snowii* [42], but different from *Thalassomedon haningtoni* [13] (S2 Fig) and *Tuarangisaurus keyesi* [63] which exhibit a modest contribution to the orbital margin.

*Parietal*. Anteriorly, the parietal is dorsoventrally flat and transversally broad (Fig 5). The parietal narrows posteriorly to become mediolaterally compressed and then triradiate in cross section. The base of an eroded sagittal crest is present posterior to the anterior contact with the premaxilla. At the anterior end, the preserved crest is low and broad but becomes narrower and slightly taller above the braincase. Due to the broken distal margin of the parietal, it is unknown how high the parietal would have extended in MGUAN PA278. The posterior-half of the parietal is situated above the braincase and contacts the supraoccipital and prootic ventrally. A pineal foramen is not present.

*Squamosal*. The squamosal is a large element that encompasses the posterior and posterolateral margins of the temporal fenestra (Fig 5). The anterior ramus of the squamosal forms an interdigitating inclined suture with the jugal. A clear suture is also seen along the squamosal-quadrate articulation (Fig 10). Together, the squamosal and postorbital form the dorsal margin of the temporal bar, which is straight and without any ridges or processes, unlike *Styxosaurus browni* which presents a ridge on the dorsal margin of the temporal bar [7]. The posterior margin of the squamosal in MGUAN PA278 is oriented anterodorsally above the quadrate (Figs 7 and 10). The dorsal rami of both squamosals are not preserved. The paroccipital process of the exoccipital contacts the squamosal medially (Fig 4). In right lateral view of the skull (Fig 10) the posterior margin of the squamosal lacks a posterior process that is present in *Nakonanectes bradti* [41], *Styxosaurus snowii* [42], and *Styxosaurus browni* [7].

*Quadrate*. The quadrate articulates with the squamosal and the quadrate process of the pterygoid, with partial contact from the paroccipital process along its dorsomedial margin (Fig 4). Two condyles are evident on the distal end of the quadrates for articulation with the mandibular glenoid. A shallow sulcus separates the condyles of the quadrate.

#### The palate

*Vomer*. The vomer contacts the medial border of the premaxilla and becomes constricted between the internal nares (Fig 6). In cross-section, the vomer is a dorsoventrally flat element anteriorly, but triradiate between the internal nares, with a sharp ridge along the dorsal surface that parallels the medial contact between the paired vomers (Fig 8). The vomer contributes to the anterior and medial border of the internal naris, while the lateral margin of the internal naris is bordered by a palatal extension from the maxilla (Fig 6). In ventral view, the vomer does not have a ventral ridge, as in *Libonectes morgani* (D1-8213) [71]. The vomer extends posteriorly beyond the internal naris and is not separated medially by the pterygoids (Fig 6), as in *Lagenanectes richterae* [11], and the aristonectines *Morturneria seymourensis* [67, 68] and *Aristonectes parvidens* [70].

*Pterygoid*. Posterior to the vomer, the pterygoid is a large element bordered laterally by the palatine (Fig 6). The pterygoids are morphologically complex elements that form an elongated wedge along the midline of the palate, contact the basicranium, and extend to the quadrates. There is no anterior interpterygoid fenestra, as in all elasmosaurids, but common in polycotylids and leptocleidids [61, 72]. The pterygoids become separated medially by a posterior interpterygoid fenestra, fully exposing the parabasisphenoid (Fig 6). The dorsal margin of the pterygoid exhibits a vertical process posterior to the epipterygoids (Figs 7D, 8D and 9E). The vertical process of the pterygoid is curved, with the medial side slightly concave and the lateral side slightly convex (Figs 8D and 9E). Lateral to the sella turcica, the pterygoid is contacted by the basipterygoid process of the basisphenoid (Figs 8D and 9E). Posterior to the sella turcica, the basipterygoid process terminates and the vertical process of the pterygoid decreases in height to form a sharp and low dorsal ridge. Thus, in cross-section the pterygoid appears as a triradiate bar that parallels the basicranium, separating the interpterygoid fenestra from the temporal fenestra (Fig 9F and 9G). Lateral to the interpterygoid fenestra, the pterygoid is broad in being nearly the same width mediolaterally as the interpterygoid fenestra and forms a trough along the ventral surface (Figs 6 and 9E–9G). A trough or excavation along the ventral surface is a common characteristic among elasmosaurids [10, 13, 41, 71, 73, 74].

The pterygoids extend medially posterior to the interpterygoid fenestra but do not contact each other ventral to the basioccipital (Fig 6). The keel of the parasphenoid is elongate and prevents medial contact between the pterygoids posterior to the interpterygoid fenestra. A lack of medial contact between the pterygoids posterior to the interpterygoid fenestra contrasts with *Libonectes morgani* [61], *Callawayasaurus colombiensis* [10], *Nakonanectes bradti* [41], and *Lagenanectes richterae* [11], but occurs in aristonectine elasmosaurids: *Aristonectes quiriquinensis* Otero, Soto-Acuña, O’Keefe, O’Gorman, Stinnesbeck, Suárez, Rubilar-Rogers, Salazar, and Quinzio-Sinn, 2014 [74], *Alexandronectes zealandiensis* Otero, O’Gorman, Hiller, O’Keefe, and Fordyce, 2016 [75], and *Morturneria seymourensis* [67, 68, 75].The quadrate process of the pterygoid is ellipsoidal in cross-section, with the long axis oriented dorsoventrally, and a compressed anteroposterior axis. The distal end contacts the quadrate along its ventromedial margin (Fig 4). This condition is distinct from that of aristonectine elasmosaurids, which exhibit a plate-like morphology, with extensive anteroposterior contact between the pterygoid and the squamosal in addition to the quadrate [76]. Contact between the pterygoid and the medial margin of the quadrate is modest in MGUAN PA278 compared to the dorsoventrally extensive contribution of the pterygoid to the medial margin of the quadrate in *Libonectes morgani* (SMU SMP 69120) [61], where the pterygoid contributes to the entire medial margin of the quadrate and squamosal in occipital view (S6 Fig).

*Epipterygoid*. In cross-section, the epipterygoid is a mediolaterally compressed element that sutures with the anterior end of the vertical process of the pterygoid. The apex of the epipterygoid is oriented anterodorsally. Few elasmosaurid specimens preserve an epipterygoid, making comparisons highly limited. In *Thalassomedon haningtoni*, the epipterygoid exhibits a more smoothly convex distal margin (S2 Fig). *Libonectes morgani* and *Tuarangisaurus keyesi* both exhibit epipterygoids that are similar to MGUAN PA278 in being mediolaterally thin elements with a triangular outline in lateral view and a distal margin oriented anterodorsally [49, 63, 71, 77].

*Ectopterygoid*. The ectopterygoid is located posterior to the palatine and lateral to the pterygoid, forming the anterior margin of the temporal fenestra (Fig 6). A ventral flange is evident on the posterior margin of the ectopterygoid in palatal view without contribution from the pterygoid (Fig 6). The flange is slightly wider than long (length: 21.7 mm; width: 27.1 mm). A dorsal extension of the ectopterygoid contacts the medial surface of the maxilla and the jugal.

*Palatine*. The palatine is sub-rectangular in shape and provides a small contribution to the posterior border of the internal naris, with contact along the medial margin of the maxilla (Fig 6).

*Internal naris*. The internal naris is formed by the vomer medially, the maxilla laterally, with a minor contribution from the palatine at its posterior margin (Fig 6). The shape of the internal nares in MGUAN PA278 is ellipsoidal in outline as in *Lagenanectes richterae* [11], but not teardrop shaped as in *Libonectes morgani* [61] and *Futabasaurus suzukii* Sato, Hasegawa, and Manabe, 2006 [78]. The shape of the internal nares in MGUAN PA278 is also distinguishable from *Tuarangisaurus keyesi*, which exhibit a concave medial margin and a convex lateral margin [63]. The internal nares are overlapped by the external nares in dorsoventral view.

#### Braincase and basicranium

*Parasphenoid*. The anterior end of the parasphenoid presents an elongated cultriform process, separating the pterygoids medially just anterior to the interpterygoid fenestra (Fig 6). The cultriform process is dorsoventrally flat in cross-section. Posteriorly, the parasphenoid exhibits a prominent ventral keel, visible within the interpterygoid fenestra and terminates posteriorly beneath the basioccipital. The parasphenoid extends to nearly the posterior limit of the ventral surface of the basioccipital. The ventral keel of the parasphenoid is located at mid-width on the ventral surface of the basioccipital and separates the medial expansion of the pterygoids. The elongated cultriform process and separation of the interpterygoid fenestra by the ventral keel of the parasphenoid are common features of elasmosaurid plesiosaurians [13, 41, 61, 63, 71, 77]. The parasphenoid does not develop cristae ventrolaterales beneath the basitubera as it does in Early Jurassic plesiosaurians (e.g. *Thalassiodracon hawkinsi* and *Eurycleidus arcuatus*) [79].

*Basisphenoid*. At the anterior limit of the basisphenoid are the basisphenoidales [80]. Posteriorly, these processes merge with the cristae trabeculares, to form low walls lateral to the sella turcica. The vidian sulcus for the internal carotid merges with a foramen for the branch of the cerebral carotid posterior to the basipterygoid processes on the lateral surface of the basisphenoid. At the posterior limit of the sella turcica are two openings for the passage of the left and right cerebral carotid arteries into the pituitary gland. The opening for the left cerebral carotid is located more anteriorly on the basisphenoid than its right counterpart and presents an asymmetry in the morphology of the basicranium. The basisphenoid of MGAUN PA278 exhibits separated exits for the cerebral carotid arteries, which is also shared with *Libonectes morgani* [81], *Kawanectes lafquenianum* Gasparini and Goñi, 1985 [82, 83], and *Tuarangisaurus keyesi* [63], but not *Alexandronectes zealandiensis* [75] which exhibits a single opening in the back of the sella turcica for the entrance of the cerebral carotids. The sella turcica of MGUAN PA278 exhibits a length to width ratio greater than 1:1 which is also shared by *Libonectes morgani*, while aristonectines and non-elasmosaurid plesiosaurians exhibit a sella turcica length to width ratio less than or approximately equal to 1:1 (Zverkov et al., 2017 supp. data) [81]. The sella turcica is longer than wide (length: 33.6 mm; width: 18.6 mm; sella turcica measurements from Zverkov et al., 2017 supp. material) [81] and forms a shallow depression anterior to the opening of the cerebral carotid foramen (Figs 8D and 9E).

Anterolateral to the dorsum sellae are the clinoid processes which are oriented at a low angle anterodorsally from the floor of the basicranium. Previously, the clinoid processes have been referred to as the “upper cylindrical processes” by Carpenter (1997) (pg. 205 in *Ancient Marine Reptiles*) [61], although these structures are homologous to the clinoid processes, as mentioned by O’Keefe (2006) [79] and Zverkov et al. (2017) [81]. A foramen for the abducens nerve (VI) runs through the base of the clinoid processes and is oriented anteroposteriorly. There is no notch at the back of the clivus, as is seen in the pistosauroid, *Cymatosaurus*, and the Early Jurassic plesiosaurians, *Thalassiodracon*, and *Eurycleidus* [79]. In dorsal view, the anterior margin of the sella turcica forms a u-shape, with a notch at its mid-width.

**Fig 11:**
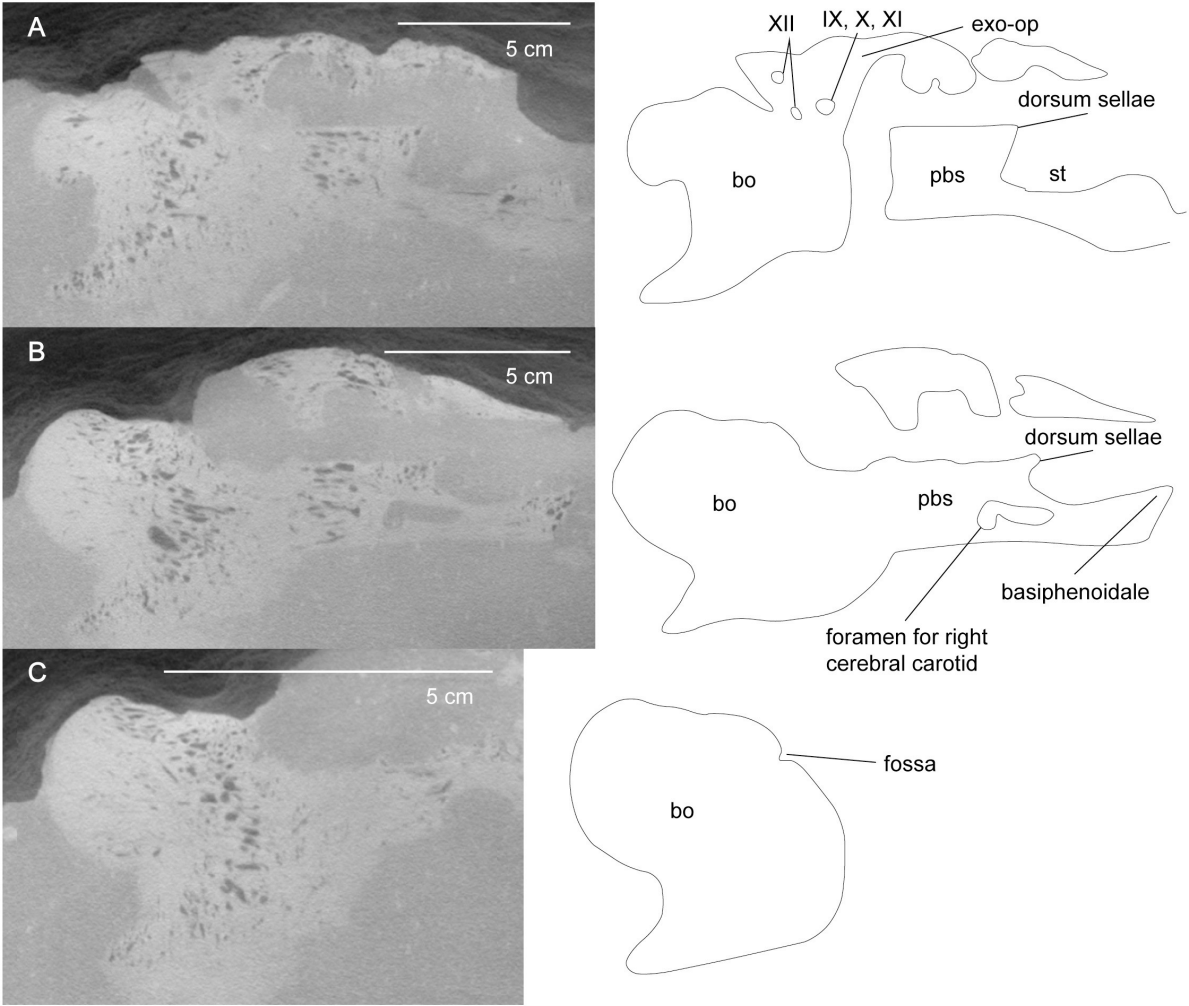
Parasagittal section of braincase from *Cardiocorax mukulu* (MGUAN PA278). A. Parasagittal section of braincase exposing cranial foramina (IX, X, XI, XII), and dorsum sellae. B. The opening of the right cerebral carotid. C. Fossa for plexus basilaris exposed on basioccipital.

*Basioccipital*. The basioccipital contacts the basisphenoid anteriorly, the exoccipitals dorsally, and is ventrally under-lapped by the parasphenoid. On the dorsal surface of the basioccipital is a fossa approximately 30 mm anterior to the posterior margin of the occipital condyle that is shallow and takes up a small surface area on the floor of the braincase (Fig 11). Zverkov et al. (2017) [81] identify the fossa as an accommodation for the plexus basilaris. This fossa is referred to as the “depression in basioccipital” in Sachs et al. (2016) [84].

The basitubera are located ventral to the occipital condyle and form a prominent ventral process. This condition of the basitubera is shared with all other elasmosaurid plesiosaurians except *Thalassomedon haningtoni* and *Nakonanectes bradti* [13, 41]. The dorsal half of the occipital condyle is slightly wider than its ventral half with no evidence of a notochordal pit. The neck of the occipital condyle is highly constricted along its ventral margin, but only moderately so on the dorsal surface, with no contribution from the exoccipital-opisthotics.

There is no evidence of a “medial foramen” near the ventral surface of the basioccipital, as described in the aristonectine, *Alexandronectes zealandiensis* [75]. There is no fontanelle evident between the suture of the basisphenoid and basioccipital. Stapes were not preserved in MGUAN PA278. Two anteroposterior canals are visible within the center of the basioccipital and may be remnants of the notochord.

*Prootic*. The prootic contacts the supraoccipital dorsally and the exoccipital-opisthotic along its posterodorsal margin. The exact location of the sutures between these three elements of the braincase could not confidently be identified from the CT data. There is also contact between the anterodorsal margin of the prootic with the parietal, although how extensive this contact is cannot be determined because sutures between the braincase and the parietal are difficult to discern and there is breakage within this region of the skull. Most of the posterior margin of the prootic forms a smooth concave outline in lateral view for the opening of the fenestra ovalis (Fig 7D). Both prootics are disarticulated from the basisphenoid at the base (Fig 9). This breakage obscures the foramen for the facial nerve (VII).

*Exoccipital-opisthotic*. The exoccipital-opisthotic articulates with the basioccipital and contacts the supraoccipital dorsally and the prootic anteriorly. Three distinct nerve foramina are apparent at the base (Fig 11). The jugular foramen is located between the suture of the exoccipital and opisthotic and would have allowed for passage of the glossopharyngeal nerve (IX), the vagus (X), and accessory nerve (XI) [85, 86]. Posterior to the jugular canal, two additional nerve canals in the exoccipital allowed for branches of the hypoglossal nerve (XII) to pass through. The hypoglossal canals completely pierce the exoccipital on both its medial and lateral surface. The presence of three distinct cranial nerve openings on both the medial and lateral surface of the exoccipital-opisthotic distinguishes MGUAN PA278 from all other elasmosaurid plesiosaurians, except *Morturneria seymourensis* [61, 63, 67, 68, 71, 75, 77, 81, 84]. *Morturneria seymourensis* has three distinct cranial nerve openings on both the lateral and medial margin of the right exoccipital-opisthotic, although the holotype exhibits an asymmetry with the medial margin of the left exoccipital-opisthotic presenting only two foramina [67, 68].

The paroccipital process extends posteriorly and ventrolaterally to contact the squamosal and quadrate. Proximally, the paroccipital process is oval in cross-section. Distally, the paroccipital process becomes significantly deeper dorsoventrally and compressed anteroposteriorly. The contact with the squamosal and quadrate is dorsoventrally expansive, similar to *Libonectes morgani* [61].

*Supraoccipital*. The supraoccipital has a smooth concave ventral margin, and a convex dorsal margin with lateral extensions to cover the prootic and exoccipital-opisthotic (Fig 9F). At the posterior extent of the supraoccipital in MGUAN PA278 is a conical posteromedian process (Fig 5), similar to *Libonectes morgani* (S6 Fig), although absent in *Terminonatotor ponteixensis* [40], and *Callawayasaurus colombiensis* (S24 Fig). MGUAN PA278 is not complete enough to assess the presence of a posteromedian ridge that extends dorsoventrally along the posterior margin of the supraoccipital above the foramen magnum.

**Fig 12:**
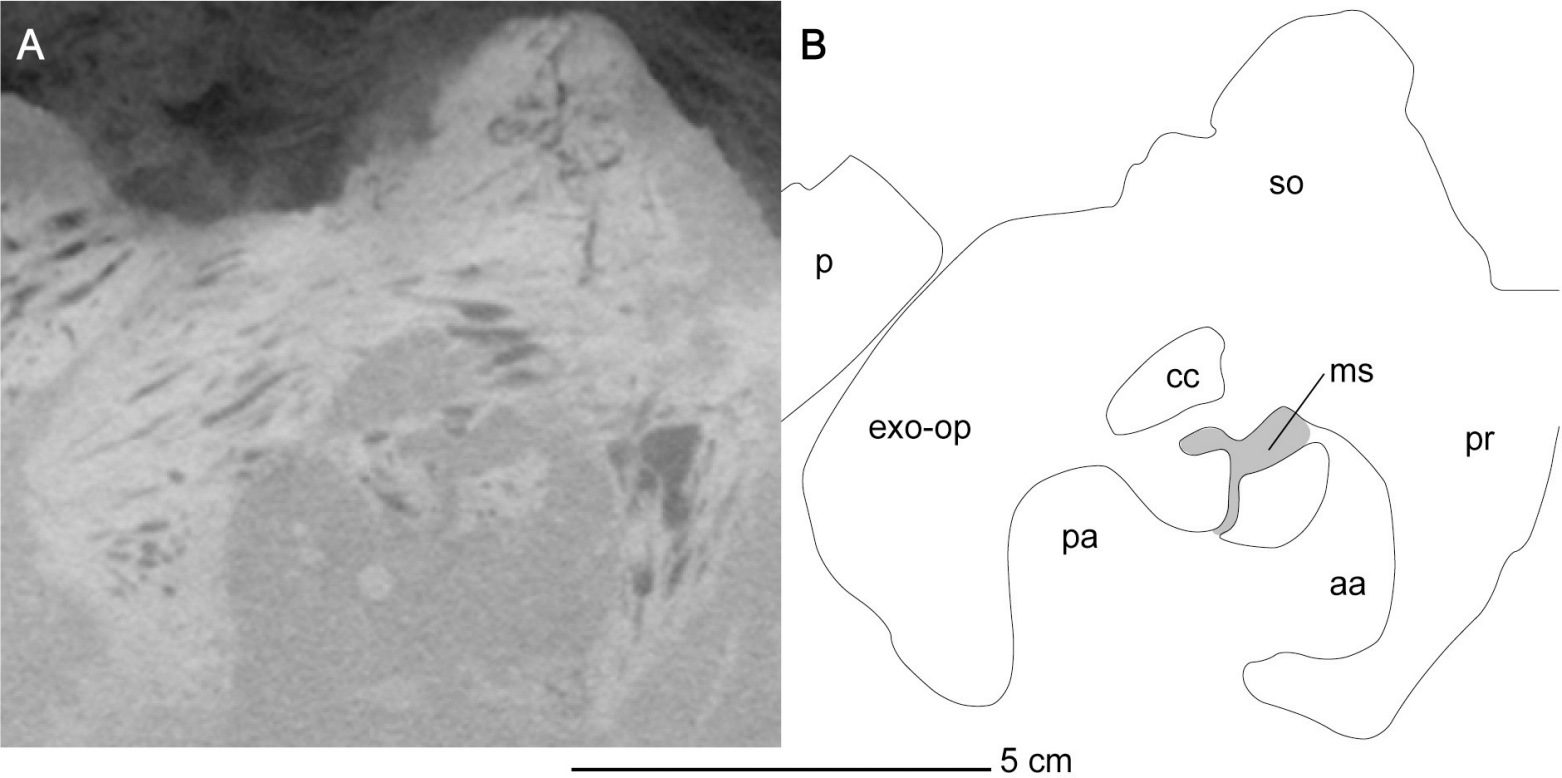
CT slice of the endosseous labyrinth of *Cardiocorax mukulu* (MGUAN PA278). Exposure of medial sinus expressed between the anterior and posterior ampullae.

**Fig 13:**
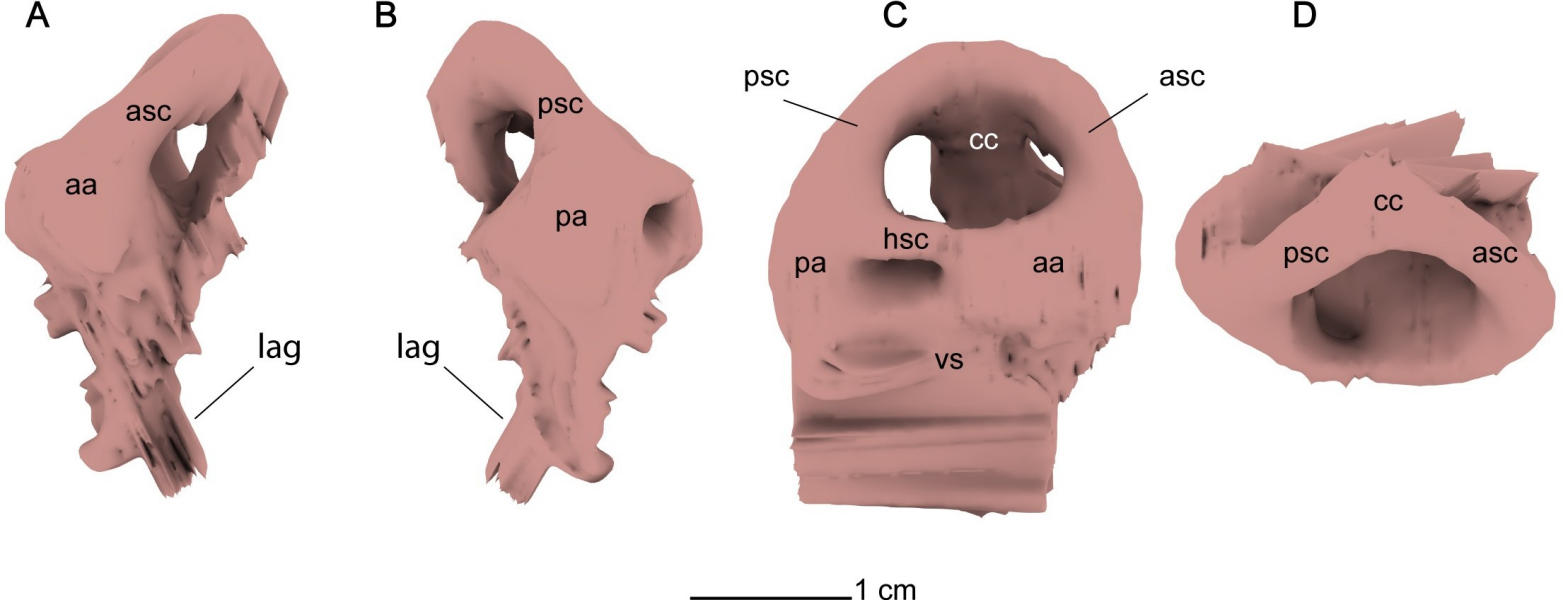
Endosseous labyrinth digital cast of *Cardiocorax mukulu* (MGUAN PA278). A. Anterior view. B. Posterior view. C. Ventral view. D. Dorsal view.

*Endosseous labyrinth*. The right endosseous labyrinth is better preserved than its left counterpart, and completely preserves the morphology of the semicircular canals and the ampullae. The vestibule and lagena in the right endosseous labyrinth are not entirely preserved. The semicircular canals, crus communis and the ampullae are thick and bulbous, typical of elasmosaurid plesiosaurians (Figs 12 and 13) [87]. An interesting feature in both the left and right endosseous labyrinth is the presence of a sinus that contacts the crus communis, the posterior ampulla, and the anterior semi-circular canal (Fig 12). A similar sinus within the endosseous labyrinth has not been figured in any elasmosaurid endosseous labyrinth cast, and is not the result of taphonomic distortion, as the bones containing the sinus are not broken or corroded, and this sinus is present in both the left and right halves of the braincase. The middle sinus (Fig 12), which is present in both left and right endosseous labyrinth of MGUAN PA278, is interpreted as a paratympanic sinus or middle cerebral vein, based on its homologous location to that of the middle sinus from *Nothosaurus marchicus* [86].

The endosseous labyrinth in MGUAN PA278 is compact, with a low aspect ratio (dorsoventral height:anteroposterior length) and is overall most similar to the endosseous labyrinth of *Libonectes morgani* and not *Callawayasaurus colombiensis* which exhibits a high aspect ratio and comparatively smaller ampullae [73, 87]. The morphology of the endosseous labyrinth in MGUAN PA278 is distinct from that of *Brancasaurus brancai* Wegner, 1914 [84, 88]. The semicircular canals of *Brancasaurus brancai* appear more gracile and bow outward further than the more compact morphology of MGUAN PA278, where the anterior and posterior semicircular canal curve smoothly to meet the crus communis and the openings between the semicircular canals are constricted [84]. The crus communis of MGUAN PA278 is short dorsoventrally, barely extending further distally than the ampullae (Fig 13). The horizontal semicircular canal is significantly atrophied relative to the anterior and posterior semicircular canals. This condition is similar to *Libonectes morgani* [73, 87].

**Fig 14:**
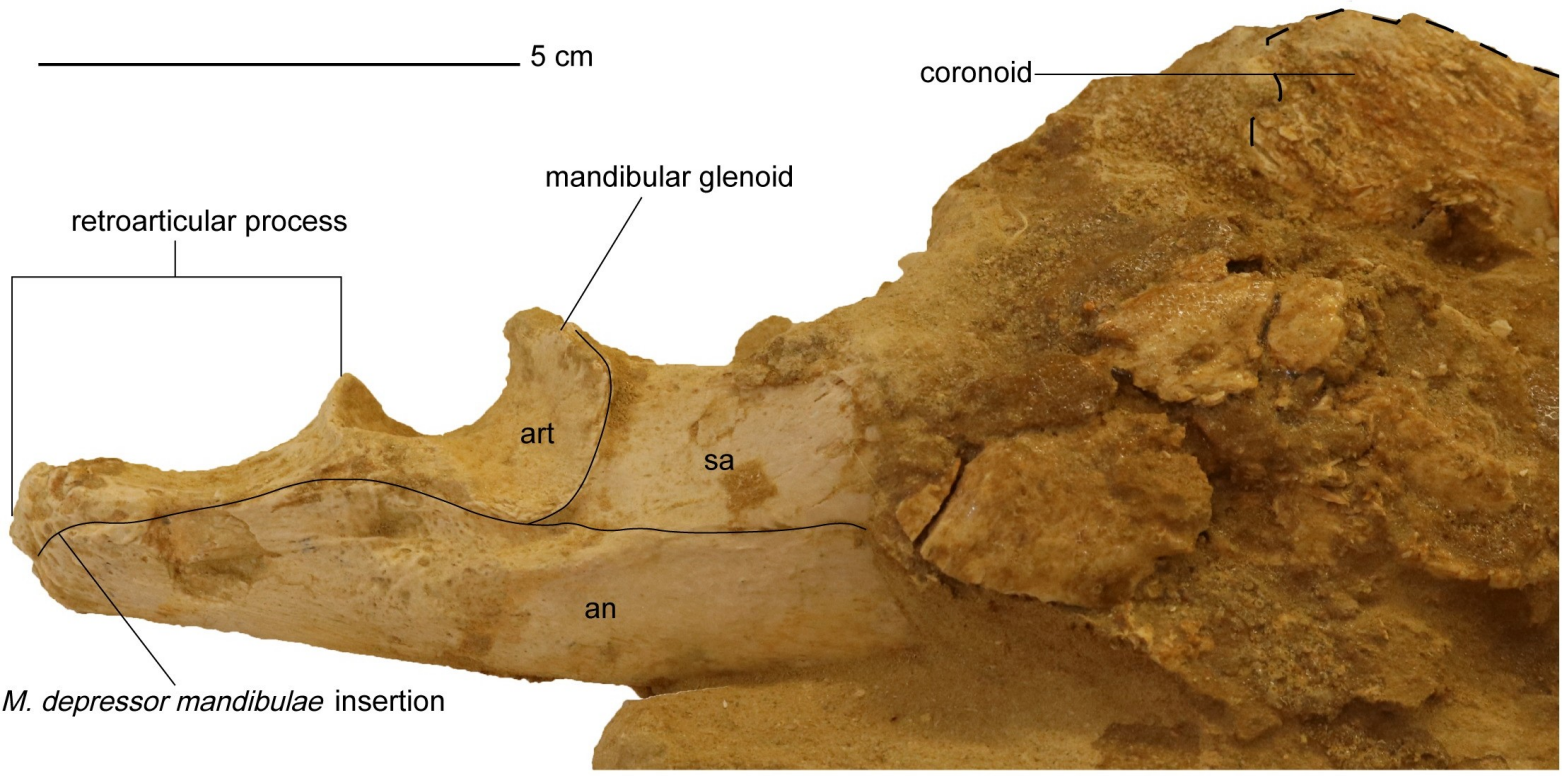
Right retroarticular process and mandibular glenoid of *Cardiocorax mukulu* (MGUAN PA278).

**Fig 15:**
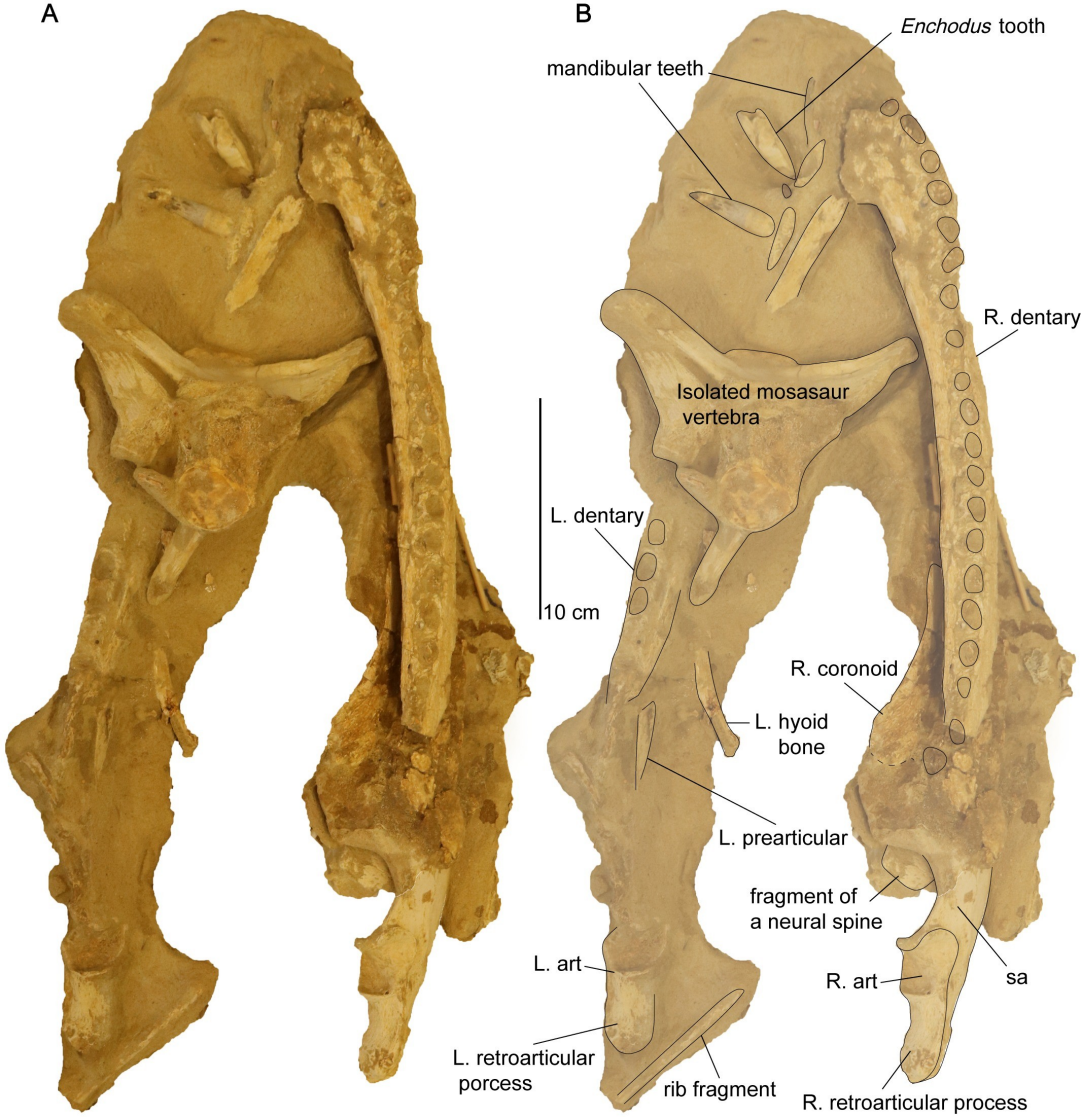
Dorsal view of the mandible from *Cardiocorax mukulu* (MGUAN PA278). A. Uninterpreted view. B. Individual bone elements labeled.

*Mandible*. The mandible is nearly complete, with weathering evident on the lateral margin of the right ramus (Fig 14). The left coronoid process is also eroded, and the left ramus appears to be broken by an overlying mosasaur vertebra (Fig 15). The maximum anteroposterior length of the mandible is 45.3 cm from the preserved anterior margin of the symphysis to the right retroarticular process. The left ramus of the mandible measures 44.5 cm from the symphysis to the left retroarticular process.

*Dentary*. The dentary is elongated and slightly bowed out laterally in dorsal view of the mandible (Fig 15). The dentary makes contact with the coronoid, splenial, and angular on its medial margin. There are at least 20 alveoli present in the dentary, like *Futabasaurus suzukii* (20 alveoli) [78], and possibly *Tuarangisaurus keyesi* (20–21 alveoli) [63]. This is contrasted with 18–19 dentary alveoli in *Libonectes morgani* [61], at least 18 in *Thalassomedon haningtoni* (S2 Fig), 16–18 in *Styxosaurus snowii* [42] (S3 Fig), 18–19 in *Nakonanectes bradti* [41], 17–18 in *Terminonatator ponteixensis* [40], 42–44 in *Kaiwhekea katiki* [66], approximately 50 or more in *Aristonectes quiriquinensis* [76], and 63–65 in *Aristonectes parvidens* [70]. The dentary alveoli of MGUAN PA278 are slightly ellipsoidal in shape, with compression along the labio-lingual axis. The alveoli are aligned close to each other, with only a slim border of dentary bone four millimeters or less present between adjacent alveoli. A row of foramina to accommodate the formation of replacement teeth is present medial to the alveoli. The row of alveoli extends from the symphysis to approximately the mid-point of the coronoid process (Fig 15).

**Fig 16:**
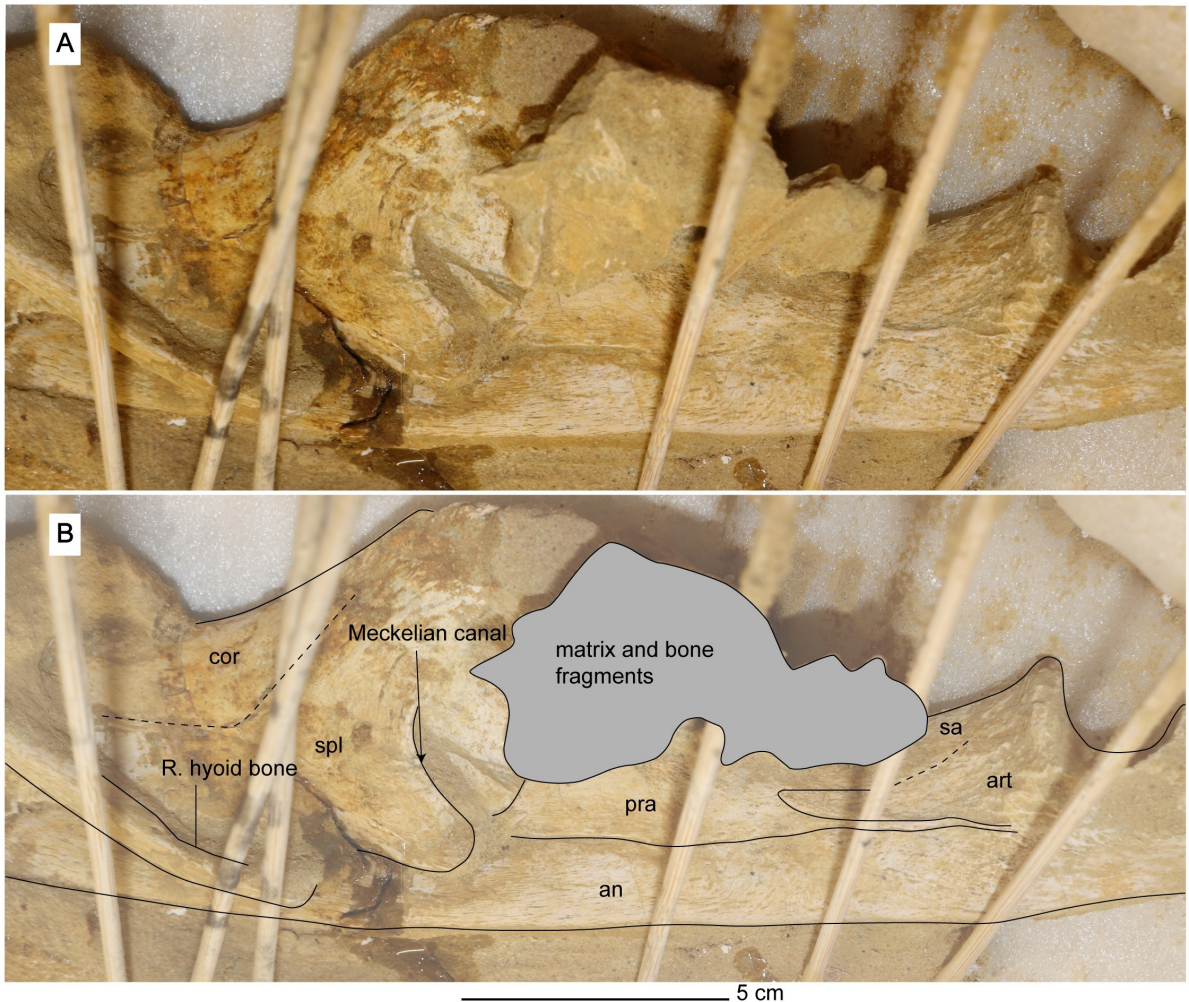
Medial view of right ramus of the mandible of *Cardiocorax mukulu* (MGUAN PA278). Wooden sticks provide additional stability.

*Angular*. The angular is elongated and is contacted dorsally by the splenial, prearticular, and articular (Fig 16). Posteriorly, the angular forms the ventral margin of the retroarticular process and contributes significantly to its lateral surface (Fig 14). It cannot be seen in MGUAN PA278 if the angular contributes to the mandibular symphysis. In lateral view of the angular, a depression is evident ventral to the mandibular glenoid and is bordered ventrally by a low ridge of bone that continues anteriorly for approximately 18 mm (Fig 14).

*Articular*. The articular forms the mandibular glenoid and is contacted medially by the prearticular, anteriorly by the surangular, and ventrally by the angular (Figs 14 and 16). Posteriorly, the articular forms the dorsal margin of the retroarticular process (Fig 14).

*Prearticular*. The prearticular is located anteromedially to the articular and dorsal to the angular (Fig 16). The prearticular tapers anteriorly and does not contribute significantly to the opening of the Meckelian canal (Fig 16).

*Splenial*. The splenial is located on the medial side of the mandible and forms a dorsoventrally deep opening for the Meckelian canal (Fig 16). The anterior extent of the splenial cannot be determined in the current state of MGUAN PA278.

*Coronoid*. The coronoid forms the apex of the coronoid process (Figs 14 and 16). The coronoid process is also formed medially by the coronoid and splenial, while the lateral side is formed by the dentary and surangular. The mandible is deepest at the apex of the coronoid process (8.3 cm in depth). The coronoid extends anteriorly however its anterior extent cannot be determined in the current state of MGUAN PA278.

*Mandibular glenoid*. The mandibular glenoid is divided into two cotyles on its articular surface, one medially and a second laterally. The anterior and posterior borders of the mandibular glenoid are expressed as high flanges of bone with the anterior flange curving slightly inward towards the center of the glenoid, while the posterior flange is oriented straight vertically (Fig 14). The medial cotyle is separated from the lateral cotyle on the mandibular glenoid by a low and smooth convex surface oriented anteromedially-posterolaterally. The lateral cotyle is located almost completely on the lateral surface of the mandible.

*Retroarticular process*. The retroarticular process is wider than high (width: 2.2 cm; height: 1.7 cm) and is formed by the articular dorsally and angular ventrally posterior to the mandibular glenoid, making the process sub-circular in cross-section (Fig 14). The right retroarticular process is 3.8 cm long, measured from the posterior end of the process until the posterior end of the right mandibular glenoid, and is longer than the mandibular glenoid (1.7 cm). At the posterior end of the retroarticular process, the bone is highly rugose for insertion of the *M*. *depressor mandibulae* [89, 90]. Along the dorsal and ventral surface, the texture of the retroarticular process is striated.

The long axis of the retroarticular process in MGUAN PA278 projects straight posteriorly and does not present a sulcus on the dorsal surface of the retroarticular process, unlike *Nakonanectes bradti* where the retroarticular process is inflected medially and a sulcus is present on the dorsal surface [41]. The retroarticular processes of MGUAN PA278 are similar to *Aristonectes quiriquinensis* [74], which exhibits a retroarticular process that is dorsoventrally compressed (W>H). However, in *Aristonectes quiriquinensis*, the retroarticular processes are curved dorsally and slightly angled dorsomedially [74]. The retroarticular process of the holotype of *Libonectes morgani* is similar to MGUAN PA278 in being oriented straight posteriorly, with the long axis pointed slightly posterodorsally [49].

**Fig 17:**
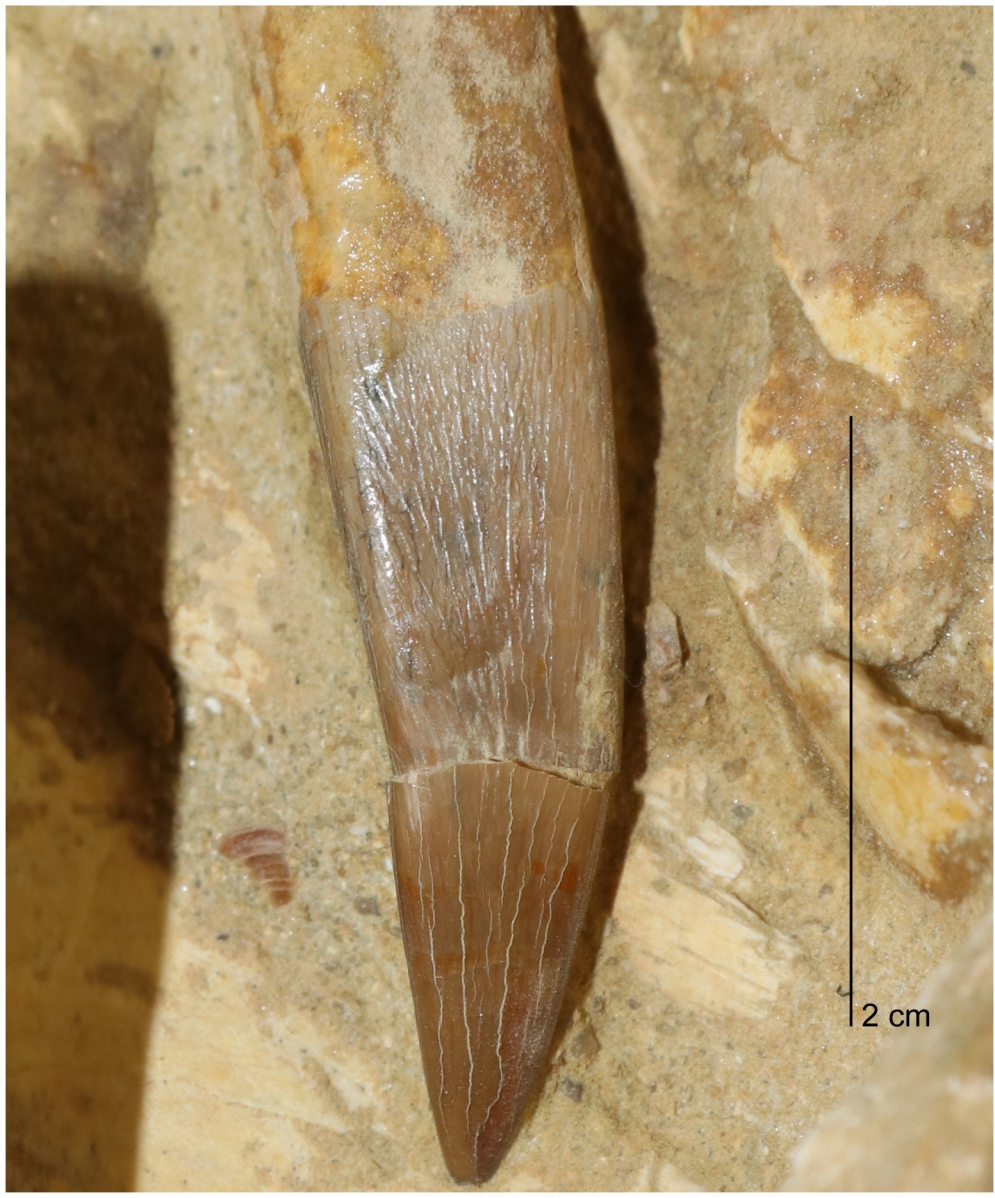
Tooth associated with *Cardiocorax mukulu* (MGUAN PA278).

*Dentition*. Twelve teeth were found associated with MGUAN PA278 displaced from the skull and mandible. Four replacement teeth remain in the cranium. Two erupted teeth remain in the mandible, one along the mandibular symphysis and a second at the anterior extent of the left ramus (Fig 15). The teeth are anisodont. All teeth are compressed along one axis and exhibit ridglets on the enamel which are oriented apicobasally [91] (Fig 17). In all observable teeth, the ridglets run subparallel to each other and taper just before the tip of the crown. Two different tooth curvature directions are evident. The anterior teeth of the mandible are curved lingually and are compressed labiolingually, thus curvature is perpendicular to the direction of compression. The second morphology, observable in the replacement teeth of the premaxillae and the loose associated teeth, shows curvature aligned more parallel to the direction of compression.

*Hyoid*. Two bones hyoid are located medial to the mandible (Fig 15). The hyoid bones of MGUAN PA278 are nearly straight and rod-like (Fig 16), similar to *Eromangasaurus australis* [15], as opposed to the more sigmoidal hyoid seen in *Nakonanectes bradti* [41].

*Axial skeleton*. A mostly complete atlas-axis complex, in addition to 17 postaxial cervical vertebrae, mostly from the anterior region of the cervical series, are preserved. The relative location of the cervical vertebrae in the vertebral column is reconstructed by the absolute size of the centra, and morphological differences between the anterior cervical vertebrae and the more posterior cervical vertebrae.

**Fig 18:**
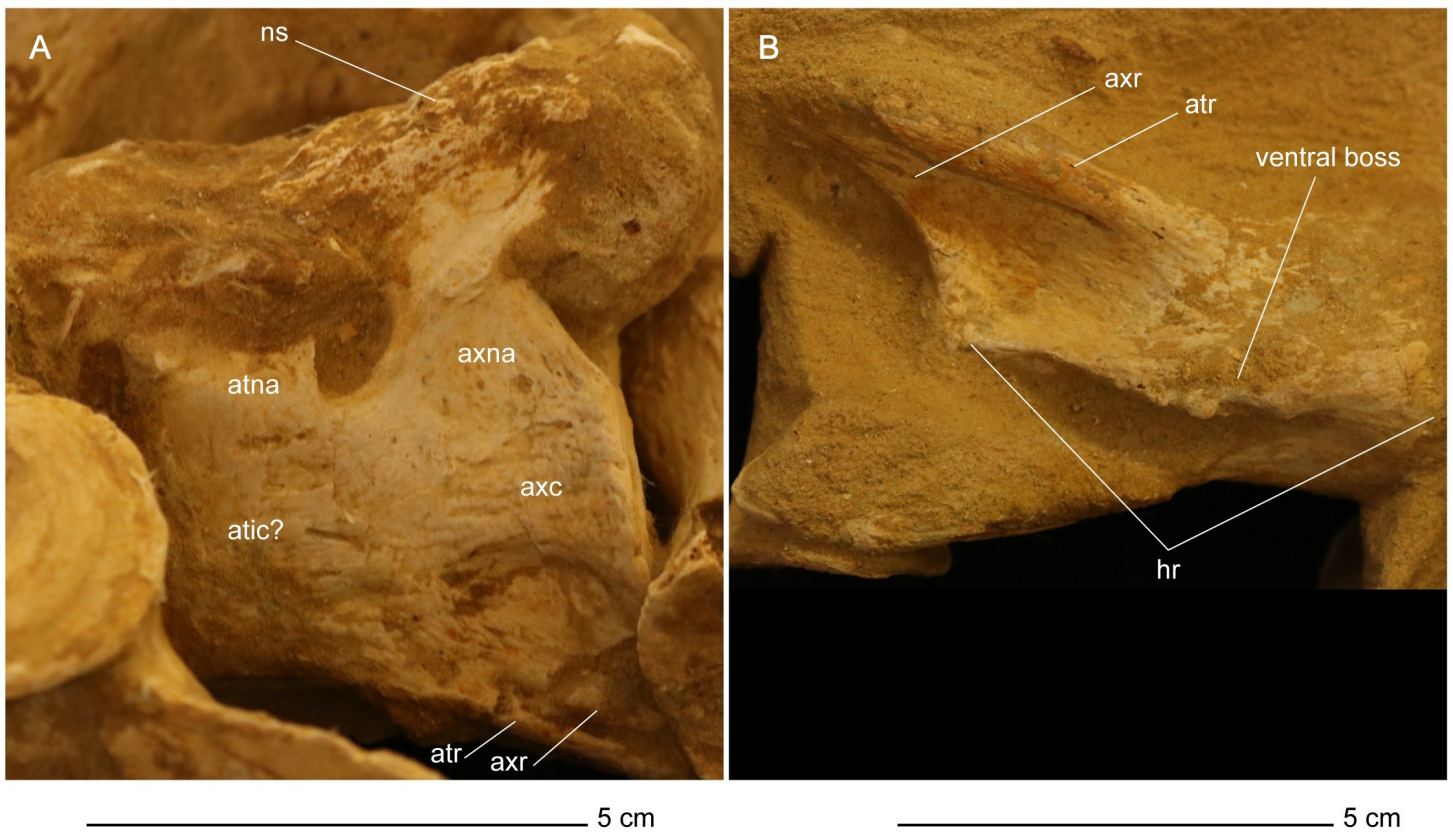
Atlas-axis complex of *Cardiocorax mukulu* (MGUAN PA278). A. Left lateral view. B. Ventral view.

*Atlas-axis complex*. The atlas and axis vertebrae are completely fused without any trace of a suture, although the atlas and axis portions of the centrum unit can be delineated by a u-shaped notch formed at approximately mid-length of the atlas-axis complex between the neural arch of the atlas vertebra and the neural arch of the axis vertebra in lateral view (Fig 18). This notch delineates the ventral margin of a fenestra that separates the neural arches. This fenestra is present in multiple elasmosaurid taxa, including *Elasmosaurus platyurus* [65], *Libonectes morgani* [92], *Nakonanectes bradti* [41], *Tuarangisaurus keyesi* [63], *Vegasaurus molyi* O’Gorman, Salgado, Olivero, and Marenssi, 2015 [93], and *Aristonectes parvidens* [70, 94]. The lateral surface of the atlas-axis complex does not present a bulge at the interface between the atlas and axis centrum, unlike *Styxosaurus snowii*, but is rather flat and smooth [42].

The pedicles of the axial neural arch extend dorsally and transition into the postzygapophyses. The neural arches of the atlas and axis are both completely fused to their respective centra without any noticeable suturing. The articular facet of the axis is amphicoelous, wider than high (W>H) and exhibits a thickened articular rim that is constricted by a shallow depression. The neural canal forms an excavation on the dorsal margin of the axial articular facet. The axis centrum becomes significantly constricted transitioning from the axial facet toward the body of the atlas-axis complex. The lateral surface of the axis centrum is rugose and grooved, with low ridges of bone oriented in a subparallel fashion anteroposteriorly. The articular facet of the atlas is narrower than the axial facet.

In ventral view, the hypophyseal ridge is a very prominent process that spans the entire anteroposterior length of the atlas-axis complex (Fig 18). The hypophyseal ridge is transversely thick at its anterior end and reaches its greatest depth below the atlas centrum. In ventral view, a swelling or ‘boss’ can be seen at the anterior end of the hypophyseal ridge. The anterior limit of the hypophyseal ridge merges with the articular facet of the atlas, as does the posterior limit of the hypophyseal ridge with the articular facet of the axis (Fig 18). The atlas and axis ribs are clearly visible and are oriented posteroventrally and posterolaterally with a narrow slit separating the atlas rib from the axis rib. The atlas rib is narrow and rod-like, while the axis rib is morphologically distinct in presenting a broad articulation with the axis centrum that tapers distally. Both the atlas and axis ribs extend beyond the axial articular facet. Overall, the morphology of the atlas-axis complex of MGUAN PA278 is morphologically similar to most known elasmosaurid atlas-axis complexes, without any distinguishing features [11, 15, 17, 41, 42, 53, 63, 70, 92, 93, 95].

**Fig 19:**
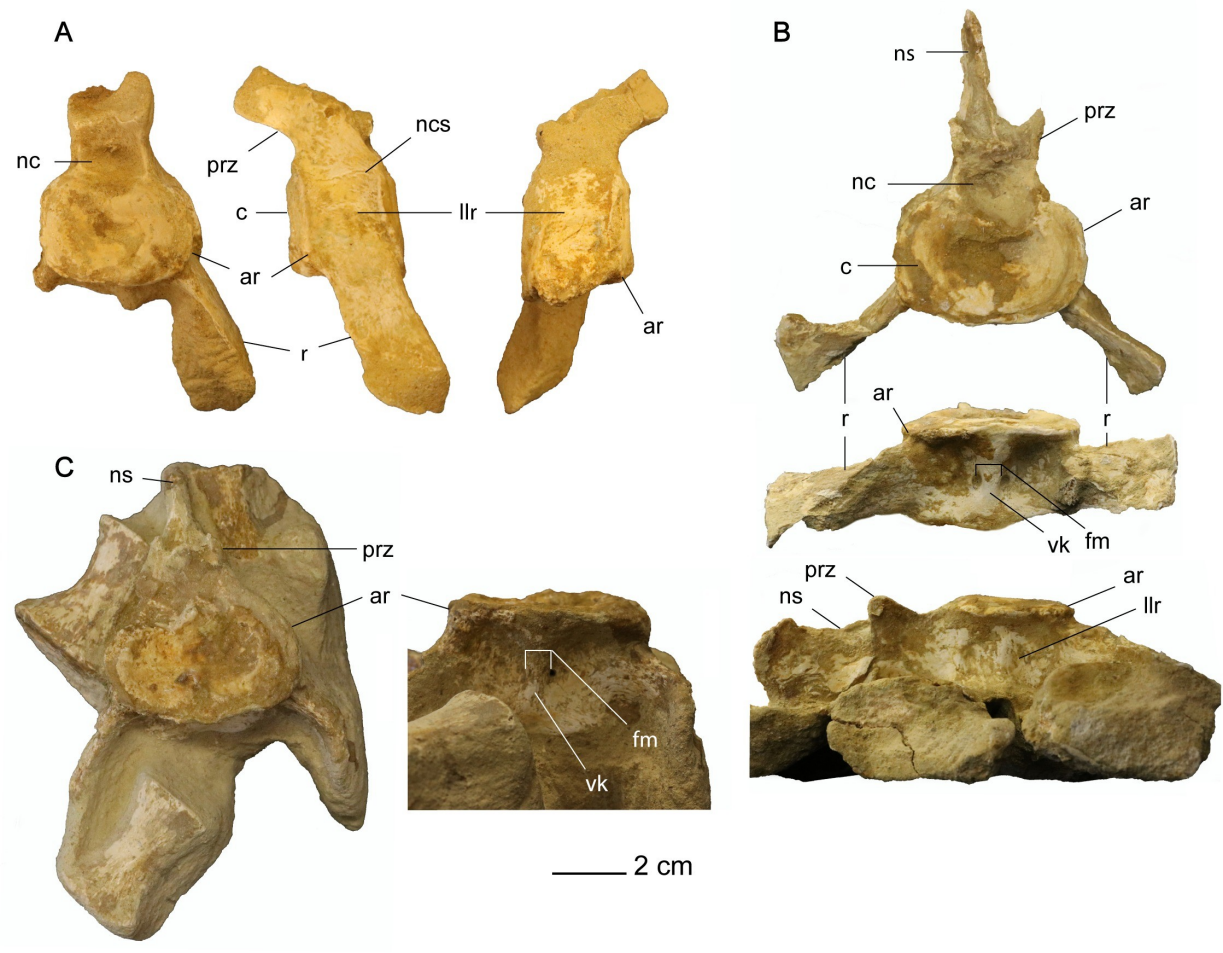
Anterior cervical vertebrae of *Cardiocorax mukulu* (MGUAN PA278). A. ‘Anterior cervical vertebra a’ in anterior, left lateral, and right lateral views (left to right) B. ‘Anterior cervical vertebra b’ in anterior, ventral, and right lateral views (top to bottom). C. ‘Anterior cervical vertebra d’ in anterior and ventral views (left to right).

**Fig 20:**
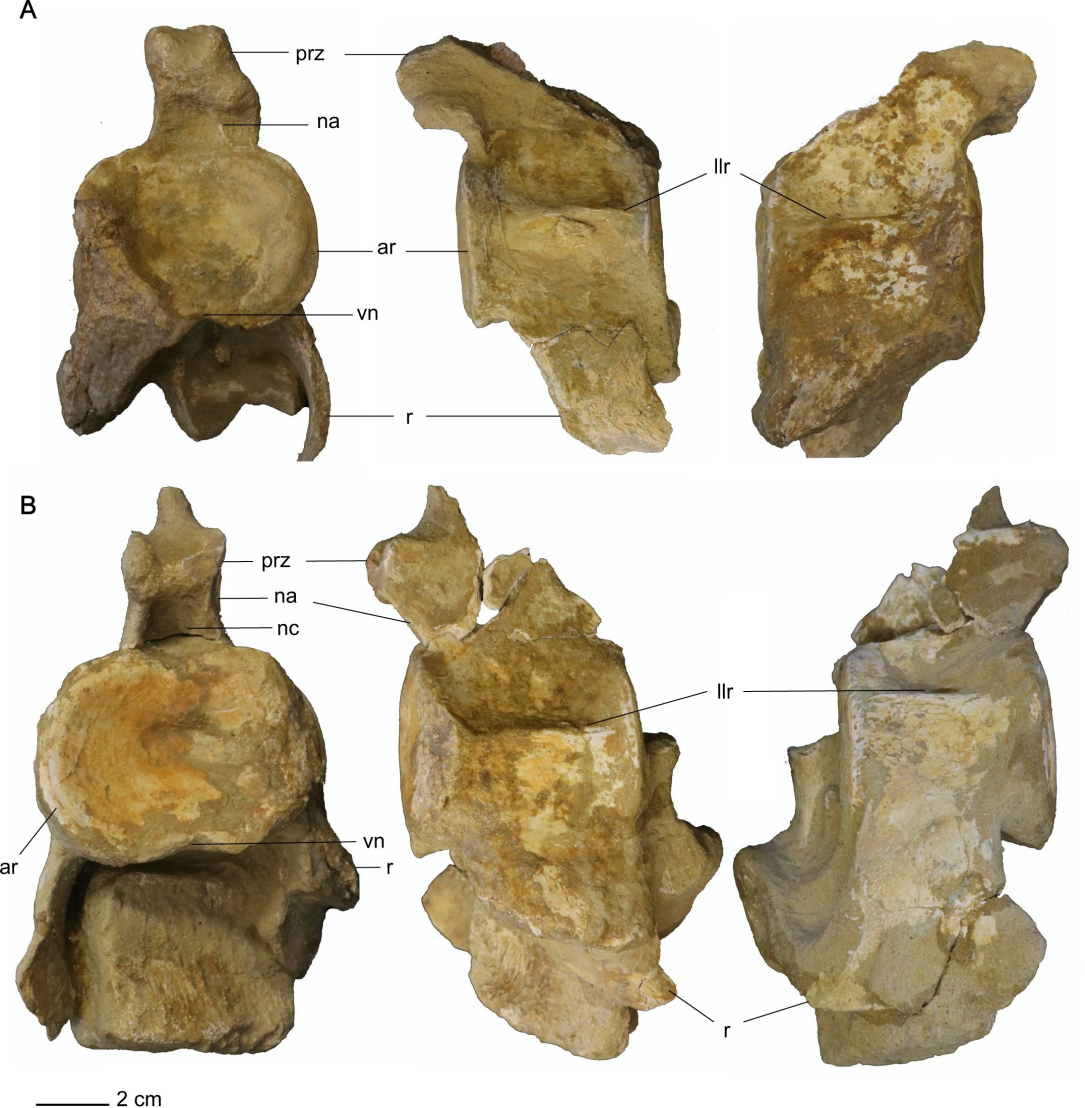
Middle cervical vertebrae a and b of *Cardiocorax mukulu* (MGUAN PA278). A. Anterior, left lateral, and right lateral view of ‘middle cervical vertebra a’ from *Cardiocorax mukulu* (MGUAN PA278). B. Anterior, left lateral, and right lateral view of ‘middle cervical vertebra b’ from *Cardiocorax mukulu* (MGUAN PA278).

*Postaxial cervical vertebrae*. The articular facets of the postaxial cervical centra exhibit a shallow excavation along the dorsal margin, formed by the neural canal, and a second shallow concavity along the ventral margin at mid-width, making the centra bilobate in anterior and posterior views (Figs 19 and 20). The articular facets of the cervical vertebrae are sub-amphicoelous with a shallow concavity situated in the center of the articular facet and exhibit a thickened articular rim that is constricted by a shallow depression. The anterior articular facet of ‘posterior cervical vertebra a’ is more platycoelous and does not exhibit an articular rim. The bilobate shape of the articular facets along with the sub-amphicoelous articular surface is sharply contrasted with the circular and platycoelous articular facets of the Hauterivian-aged elasmosaurid, *Jucha squalea* Fischer, Zverkov, Arkhangelsky, Stenshin, Blagovetshensky, and Uspensky, 2020 [12] but more similar to the centra morphology observed in Late Cretaceous elasmosaurids [7, 40, 41, 78, 92, 93, 95].

**Fig 21:**
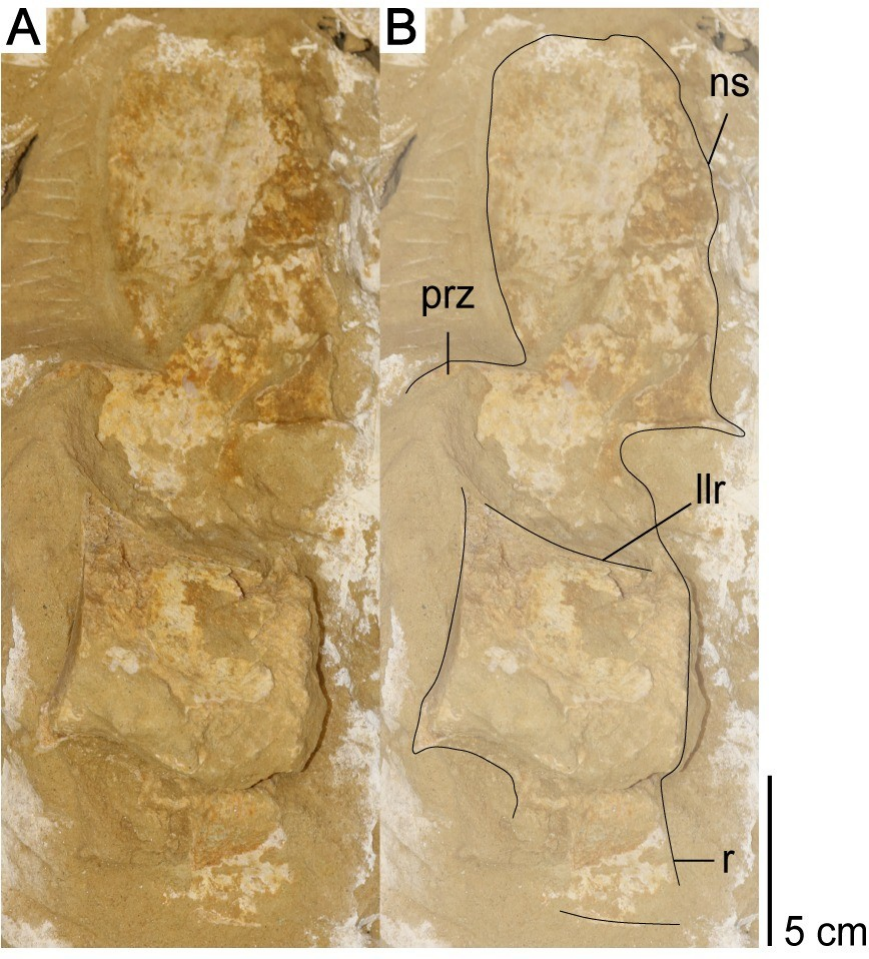
‘Posterior cervical vertebra a’ in left lateral view from *Cardiocorax mukulu* (MGUAN PA278). A. Cervical vertebra in left lateral view B. Cervical vertebra with outline and anatomical annotations.

Lateral longitudinal ridges are present at mid-height on the lateral surface of the centra (Figs 19–21). In ventral aspect, a pair of foramina is present at about mid-length of the centra and laterally straddle a prominent ventral keel that extends anteroposteriorly between the articular facets of the centra. At the interface between the keel and the articular facets, the keel splays laterally and merges with the ventral rim of the articular facets (Fig 19B and 19C). The foramina are circular in outline and are situated within sulci. All observable centra are constricted between the anterior and posterior articular facets. The neural arches and cervical ribs are fully fused to the centra. The cervical ribs present a hatchet-shaped morphology, with anterior and posterior projections at the distal end of the ribs. Only ‘anterior cervical vertebra a’ (Fig 19A) exhibits a cervical rib without an anterior projection at the distal end. Rather, the cervical rib of the ‘anterior cervical vertebra a’ is smoothly convex along its anterior margin, concave along the posterior margin, and oriented posterolaterally and ventrally (Fig 19A). The cervical ribs are all single headed without a trace of a suture. Curved ribs extend from the ventrolateral margins of the anterior and middle cervical centra, to where the distal ends are facing ventromedially (Figs 19 and 20). The postzygapophyses of the cervical vertebrae present a smoothly convex ventral surface and extend to the level of the posterior articular facet or slightly more posteriorly.

In the anterior cervical vertebrae, the prezygapophyses merge medially to form an inset surface. ‘anterior cervical vertebra b’ and ‘anterior cervical vertebra d’ both exhibit a thin ridge of bone that extends from this medial inset surface to the neural spine dorsally (Fig 19B and 19C). This same ridge dividing the prezygapophyses and transitioning to the neural spine was also noted in the anterior cervical vertebrae of *Mauisaurus haasti* Hector, 1874 [96] by Hiller et al. (2005) [97]. The neural spines from the anterior cervical vertebrae are blade-like (Fig 19). None of the anterior cervical vertebrae present ‘axial striations’ on the lateral and ventral sides of the articular facets, as is present in AMNH 1495 (*Styxosaurus* sp.) [7] or *Jucha squalea* with ridges and furrows on the lateral and ventral surface [12]. The neural spines of the anterior cervical vertebrae in MGUAN PA278 are longer than tall, as in most elasmosaurids except *Libonectes morgani* [53] and *Lagenanectes richterae* [11]. The anterior cervical vertebrae of MGUAN PA278 exhibit neural spines that curve posterodorsally, similar to *Aristonectes parvidens* [70], *Elasmosaurus platyurus* [65], and *Callawayasaurus colombiensis* [10], but distinct from *Hydrotherosaurus alexandrae* [39], *Kaiwhekea katiki* [66], and *Nakonanectes bradti* [41] which exhibit anterior cervical neural spines oriented anterodorsally.

**Fig 22:**
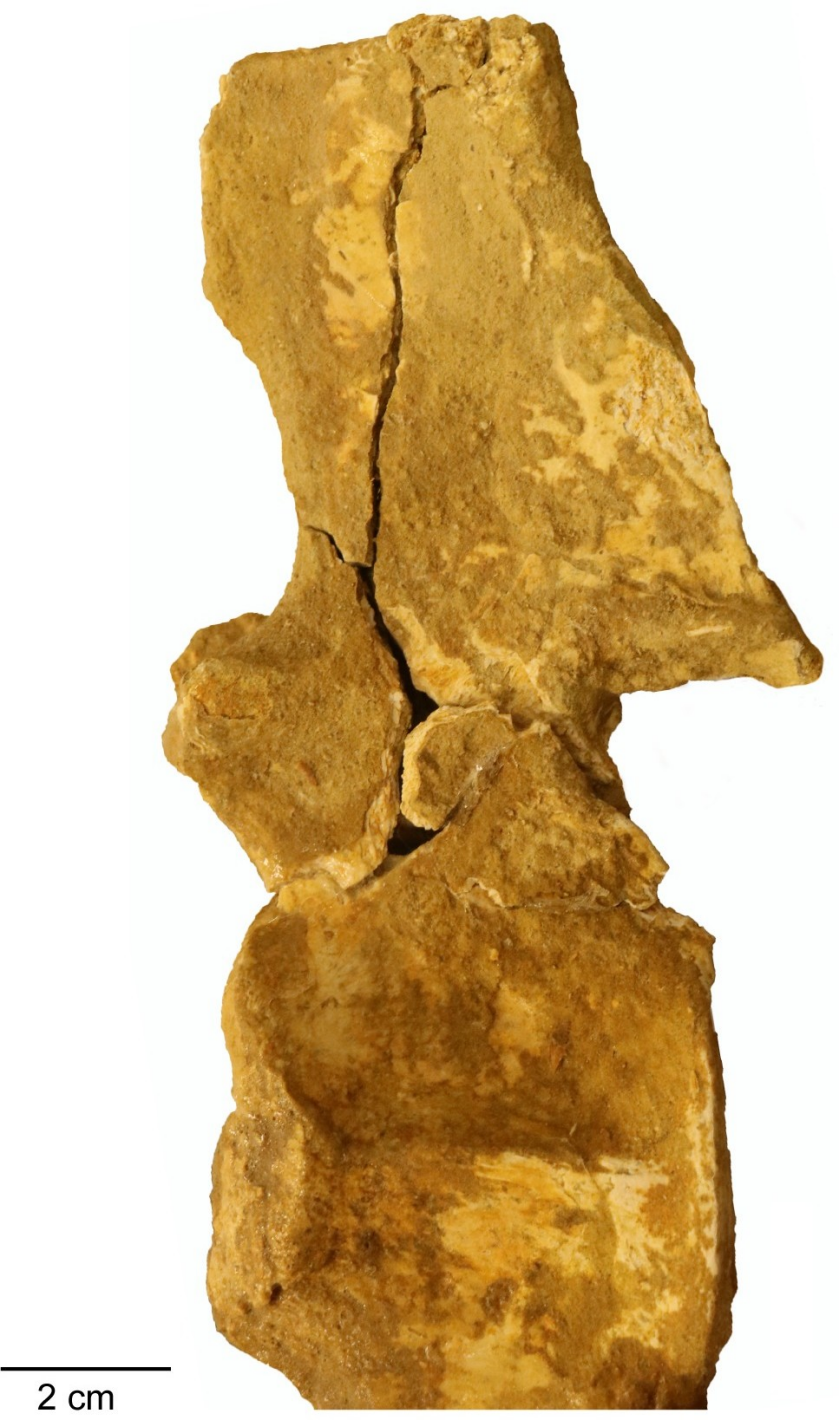
‘Middle cervical vertebra b’ with neural spine from *Cardiocorax mukulu* (MGUAN PA278). The anterior margin of the neural spine exhibitsa sinusoidal outline.

In the middle cervical vertebrae and posterior cervical vertebra, the dorsal surface of the prezygapophyses form a smooth concave articular facet to receive the postzygapophyses, however, the sharp ridge that extended between the prezygapophyses in the anterior cervical vertebrae is absent in the middle cervical vertebrae (Fig 20). The presence of this ridge in the posterior cervical vertebra (Fig 21) is obscured. The postzygapophyses are narrow and completely merge with each other medially and with the neural spine dorsally in the middle cervical vertebrae and form a smooth convexity on the ventral surface (Fig 22). The postzygapophyses of the middle and posterior cervical vertebrae extend beyond the posterior articular facet of the centra (Figs 21 and 22). The neural arches are narrow relative to the width of the centra in the middle cervical vertebrae and the posterior cervical vertebra.

The neural spine of ‘middle cervical vertebra b’ is mediolaterally compressed and blade-like (Fig 22). The apex of the neural spine is flat, without evidence of any inclination along the dorsal margin. The anterior margin of the neural spine also presents a noticeable anterior projection that is smoothly convex in outline and transitions to a concave preaxial margin in lateral view near the base of the neural spine (Fig 22). The anterior projection would have extended even further than what is preserved, as the process is eroded along its anterior margin; the posterior margin of the neural spine is damaged. The base of the neural spine in ‘middle cervical vertebra b’ is approximately equal to the length of the centrum. Within Elasmosauridae possibly *Albertonectes vanderveldei* Kubo, Mitchell, and Henderson, 2012 [95], and *Kaiwhekea katiki* exhibit middle cervical vertebrae with a sinusoidal anterior margin of the neural spines with a base that is nearly equal to that of the centrum (Fig 2 line drawing in Cruickshank and Fordyce, 2002 and Fig 3 line drawing in Kubo et al., 2012) [66, 95]. *Cardiocorax mukulu* exhibits the same sinusoidal morphology as the middle cervical neural spine as MGUAN PA278 but in a slightly more posterior position in the cervical column [26].

The width of the anterior and middle cervical centra of MGUAN PA278 is greater than their length (Table 2), similar to that of aristonectine elasmosaurids in which width of the cervical centra is significantly greater than length [27], but not elasmosaurines where the mid-cervical vertebrae are extremely elongated (two-thirds to two-times longer than the width) [7].

**Table 2:** Centrum dimensions for cervical vertebrae associated with MGUAN PA278 and MGUAN PA103.

Vertebrae dimensions of MGUAN PA278 and MGUAN PA 103	L (mid-height)	L (ventral margin)	H	W	W/H ratio	W/L (ventral margin) ratio
anterior cervical vertebra a	29.9 mm	31.1 mm	32.4 mm	46.0 mm	1.4	1.5
anterior cervical vertebra b	29.7 mm	29.9 mm	36.2 mm	53.8 mm	1.5	1.8
anterior cervical vertebra c	37.0 mm	36.6 mm	39.0 mm	51.3 mm	1.3	1.4
anterior cervical vertebra d	NA	34.2 mm	40.4 mm	51.9 mm	1.3	1.5
anterior cervical vertebra e	40.1 mm	37.9 mm	42.2 mm	58.0 mm	1.4	1.5
anterior cervical vertebra f	40.1 mm	41.2 mm	43.4 mm	61.0 mm	1.4	1.5
anterior cervical vertebra g	46.4 mm	47.1 mm	43.6 mm	62.4 mm	1.4	1.3
middle cervical vertebra a	61.5 mm	61.5 mm	53.4 mm	69.1 mm	1.3	1.1
middle cervical vertebra b	59.4 mm	55.4 mm	59.5 mm	82.2 mm	1.4	1.5
posterior cervical vertebra a	71.3 mm	NA	74.9 mm	NA	NA	NA
Fifth centrum in the cervical series of MGUAN PA103	105.7 mm	NA	77.8 mm*	NA	NA	NA

L = length; W = width; H = height. L (mid-height) refers to length being measured at mid-height of the centrum; L (ventral margin) refers to length of the centrum being measured along the midventral axis. An asterisk indicates the dimension was affected by damage.

‘Posterior cervical vertebra a’ in MGUAN PA278 lacks an articular rim around the exposed anterior articular facet, unlike the anterior and middle cervical (Figs 20 and 21). The anterior and posterior margins of the neural spine from ‘posterior cervical vertebra a’ are nearly straight (Fig 21). The height of the neural spine of ‘posterior cervical vertebra a’ (measured from the dorsal margin of the prezygapophyses to the dorsal-most margin of the neural spine) is 117.4 mm. The neural spine of the posterior cervical vertebra is significantly taller than the centrum. This condition is the same in *Albertonectes vanderveldei* [95], *Thalassomedon haningtoni* (S18 Fig), *Callawayasaurus colombiensis* [10], *Libonectes morgani* [53], *Terminonatator ponteixensis* [40], *Kawanectes lafquenianum* [98], and *Vegasaurus molyi* [93]. In contrast, *Kaiwhekea katiki* [66], *Aristonectes quiriquiensis* [74], and *Aphrosaurus furlongi* Welles, 1943 [3, 39] exhibit neural spines that are approximately equal to the centrum in height.

#### Appendicular skeleton

**Fig 23:**
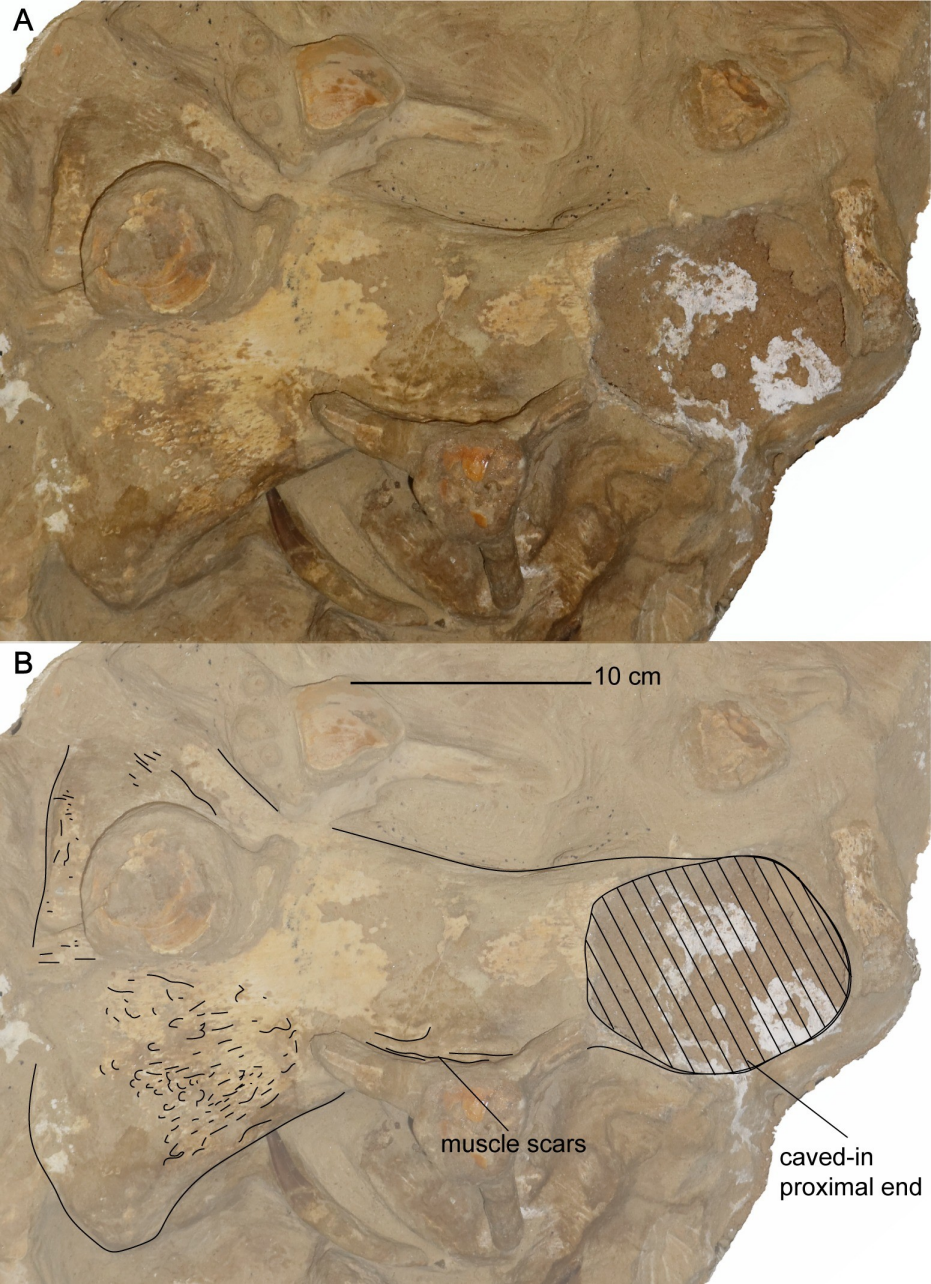
Femur of *Cardiocorax mukulu* (MGUAN PA278) in dorsal view.

*Femur*. In what is exposed of the propodial associated with MGUAN PA278, this element is indistinguishable from the femur of *Cardiocorax mukulu* (MGUAN PA270) [25, 26] and exhibits a straight shaft instead of the sigmoidal shape to the shaft that is often present in elasmosaurid humeri [7, 10, 39, 40, 99, 100]. Thus, this propodial is identified as a femur, based on the straight shaft, in addition to a calcaneum that was found associated with MGUAN PA278. The femur is exposed in dorsal view, with the dorsal half of the proximal end weathered and damaged (Fig 23). The long axis of the femur is straight without dorsal or ventral curvature. The transition from the proximal end of the femur toward the distal end is constricted at the diaphysis. The preaxial and postaxial borders of the diaphysis are straight and sub-parallel but widen toward the distal end. The distal end of the femur is substantially wider than the shaft and is covered along the dorsal surface by rugose, sub-parallel ridges. The ridges become smooth and flatten toward the diaphysis. The femur of MGUAN PA278 does not present a posterodistal expansion as in AMNH 1495 (*Styxosaurus* sp.) [7], *Terminonatator ponteixensis* [40], *Vegasaurus molyi* [93], and *Aphrosaurus furlongi* [3]. Rather, the anterodistal and posterodistal margins of the femur in MGUAN PA278 are more evenly expanded, as in *Futabasaurus suzukii* [78], *Callawayasaurus colombiensis* [10], and *Jucha squalea* [12].

**Fig 24:**
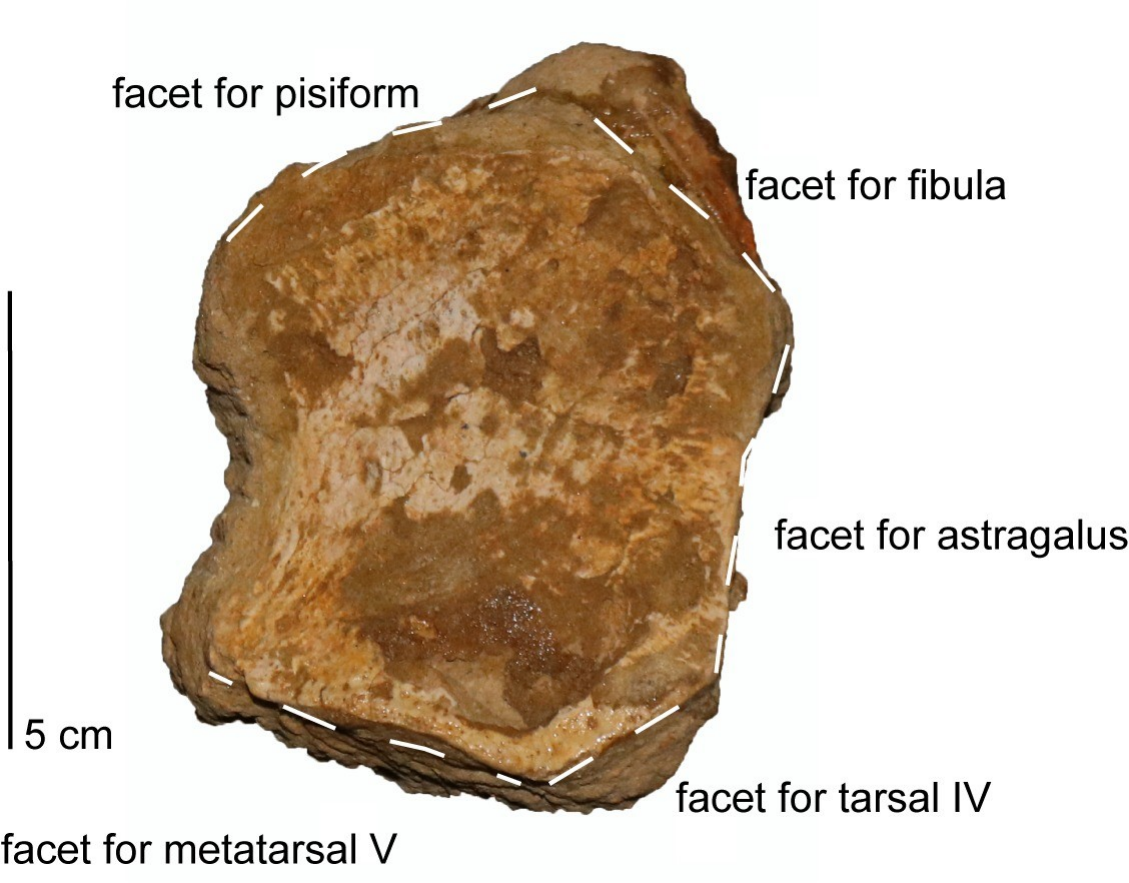
Calcaneum of *Cardiocorax mukulu* (MGUAN PA278) in ventral view facets labeled.

*Calcaneum*. The calcaneum measures 6.7 cm along its proximodistal axis and is 5.1 cm anteroposteriorly (Fig 24). The texture of the exposed surface is smooth at the center but becomes more rugose near the margins. The calcaneum is 2.9 cm thick along the proximal margin. Five discrete articular facets are present (Fig 24), as also observed in the calcaneum of *Styxosaurus* sp. (AMNH 1495) [7] and *Terminonatator ponteixensis* [40]. The posteroproximal facet would have articulated with a pisiform, like the condition of *Styxosaurus* sp. (AMNH 1495) [7]. The anteroproximal facet articulated with the fibula, the anterior facet with the astragalus, the anterodistal facet articulated with distal tarsal IV, and the posterodistal margin articulated with metatarsal V. The posterior border is smoothly concave and would have contributed to part of the posterior margin of the paddle or perhaps accommodated an additional pisiform.

**Fig 25:**
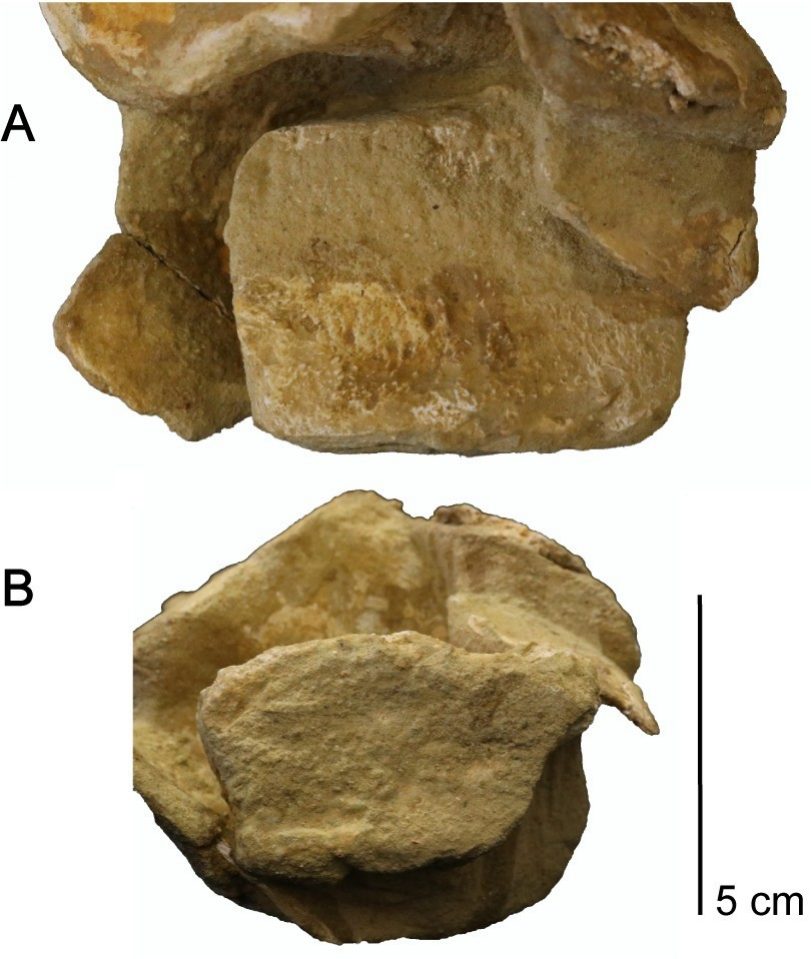
Distal tarsal element of *Cardiocorax mukulu* (MGUAN PA278). A. Dorsal/ventral view. B. Lateral view.

*Distal tarsal element*. The element presents a rectangular outline in dorsoventral view (Fig 25). The exposed bone is thick dorsoventrally and the surface is rugose.

**Fig 26:**
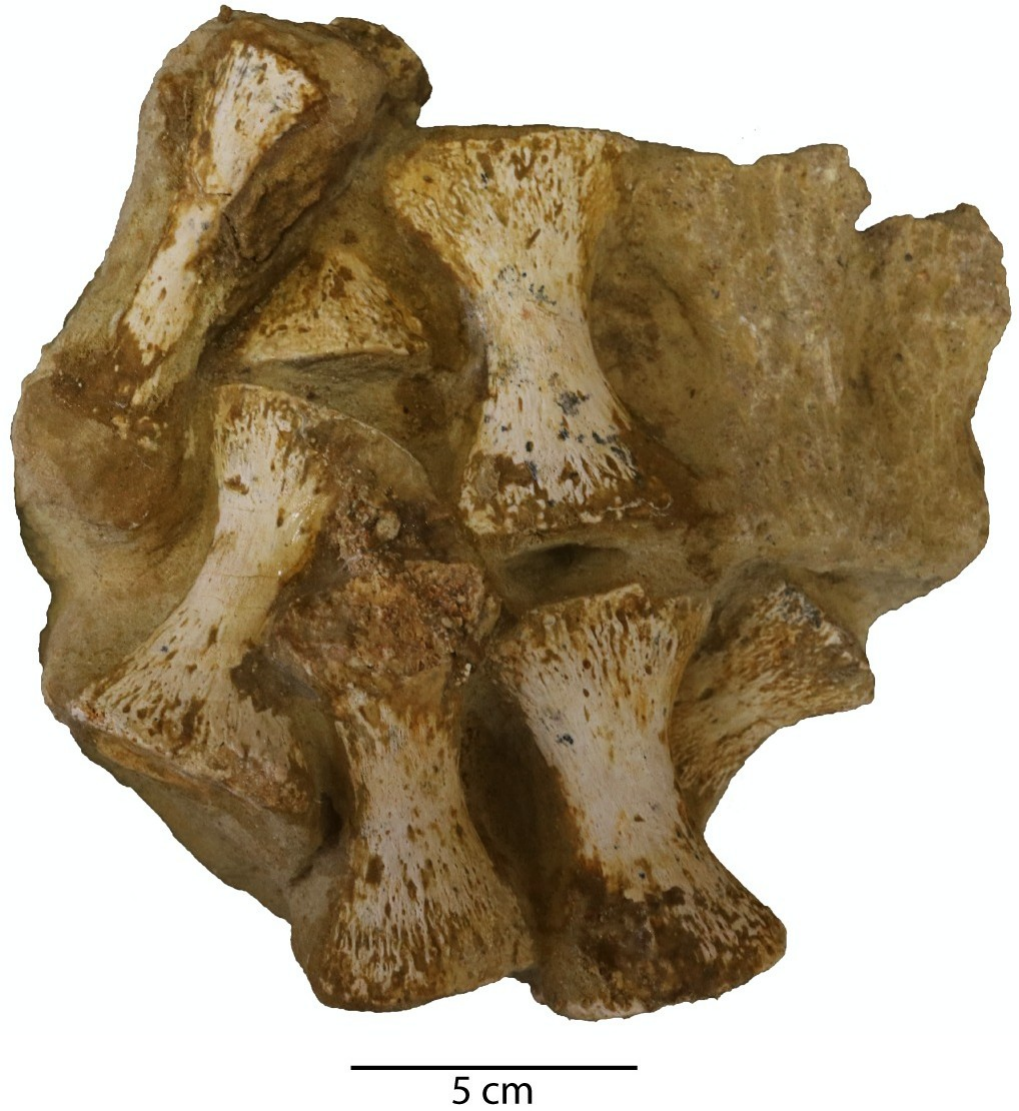
Semi-articulated phalanges of *Cardiocorax mukulu* (MGUAN PA278).

*Phalanges*. The phalanges exhibit the usual plesiosaurian spool shape with wide distal ends and a constricted mid-section (Fig 26). The texture of the mid-section of the phalanges is smooth but the distal ends are rugose with low ridges of bone that run sub-parallel to each other. The distal and proximal articular facets of the phalanges are flat with no foramina present. The phalanges are slightly compressed along the dorsal-ventral axis. The phalanges exhibit a short and robust morphology, where the proximodistal length is less than twice as long as the anteroposterior width [4].

### Taxonomic identification of MGUAN PA278

The following traits assign MGUAN PA278 to Elasmosauridae: lateral ridge on anterior cervical vertebrae [3, 6], closed pineal foramen [6], ventral surface of cervical vertebrae bears rounded ridge [3], anterior to middle cervical vertebrae substantially longer than high [3], premaxilla excluded from border of internal nares [72], vomer extends posterior to internal nares [72], number of cervical rib heads reduced to one [72]; combined width of cervical zygapophyses distinctly narrower than centrum [7]; absence of anterior interpterygoid vacuity [31], and cervical vertebrae with a ventral notch giving the cervical vertebrae a bilobed articular facet [7].

**Fig 27:**
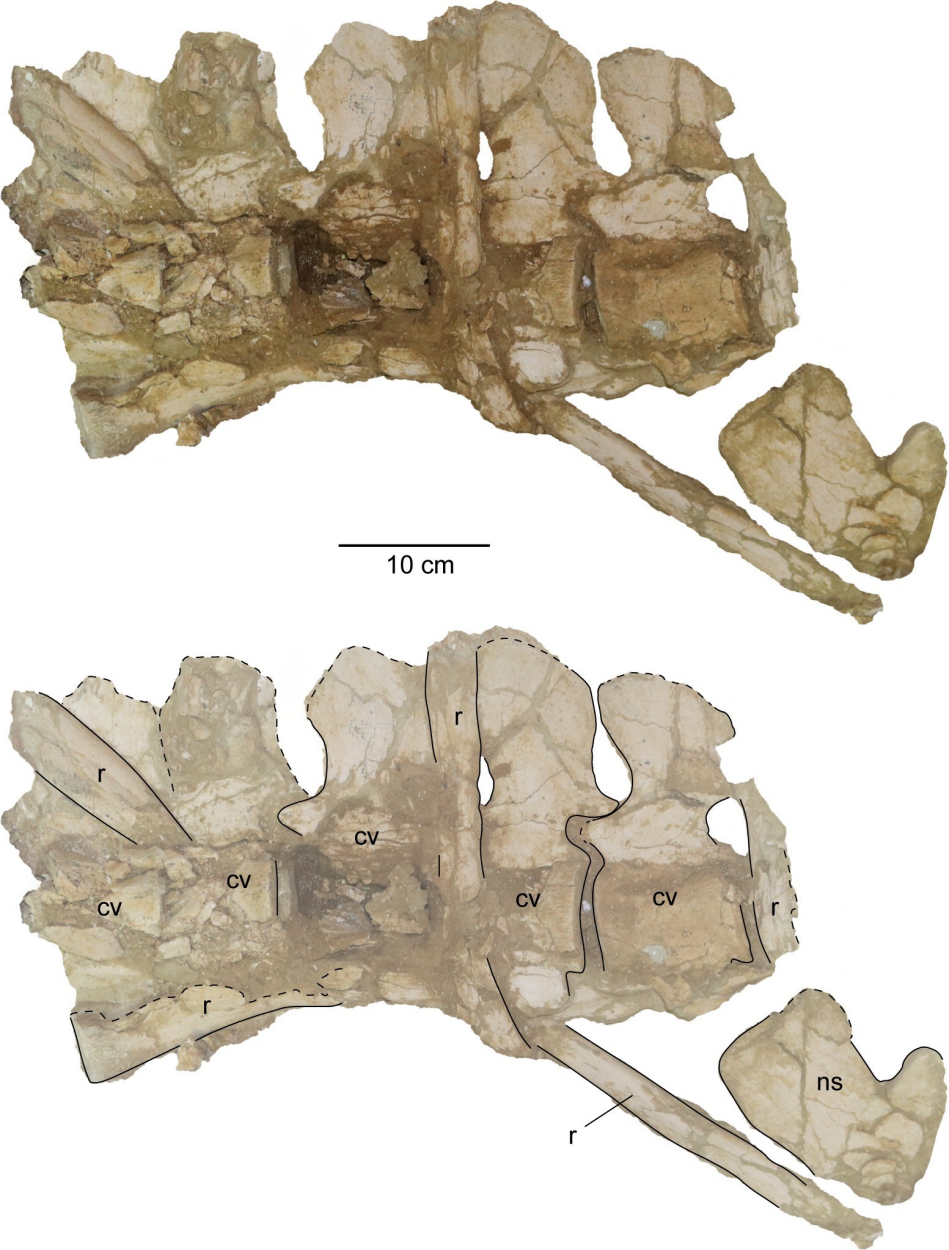
Posterior cervical series from the holotype of *Cardiocorax mukulu* (MGUAN PA103). The anteroposteriorly elongated neural spines nearly touch adjacent neural spines and form a sinusoidal anterior margin that in combination with other characters is diagnostic of *Cardiocorax mukulu*.

The anterior cervical vertebra associated with MGUAN PA103 (Fig 2 in Araújo et al., 2015) [26] is morphologically indistinguishable from the anterior cervical vertebrae of MGUAN PA278. MGUAN PA278 shares with the holotype of *Cardiocorax mukulu* (MGUAN PA103) a single apomorphy where the morphology of the neural spines allows for near contact between adjacent neural spines. This condition is derived from anterior and posterior projections along the margins of the neural spines (Fig 27). Araújo et al. (2015) [26] state that a posterior projection of the neural spine is shared between *Cardiocorax mukulu* and *Callawayasaurus colombiensis*; however, the anterior projection of the neural spine is autapomorphic for *Cardiocorax mukulu*. Thus, a more appropriate wording for this character state is here proposed: middle and posterior cervical neural spines are broad at the base (subequal to the length of the centrum) and exhibit a sinusoidal outline along the anterior margin in lateral view. *Kaiwhekea katiki* and *Albertonectes vanderveldei* may also exhibit middle cervical vertebrae with anteroposteriorly broad neural spines and a sinusoidal outline along the anterior margin [66, 95]. Thus, we do not consider this character an autapomorphy for *Cardiocorax mukulu*, it is however still useful for diagnosis.

The only other known elasmosaurid taxon represented at Bentiaba is an aristonectine gen et sp. indet. [27]. The aristonectines from Bentiaba are dwarf elasmosaurids that exhibit a skeletal anatomy readily distinguishable from *Cardiocorax mukulu* [27]. The centrum and cervical ribs, as well as the neural arches, of adult aristonectine elasmosaurids from Bentiaba lack fusion [27]. The cervical centra of the aristonectine elasmosaurids from Bentiaba also lack an articular rim encircling the articular facets of the centra, are sub-platycoelous, and lack a lateral longitudinal ridge [27].

Moreover, magnetostratigraphy supported by the δ^13^C stratigraphy, from the stratigraphic section at Bentiaba places the locality that produced MGUAN PA278 within the top of magnetochron C32n.1n, (71.64–71.40 Ma) the same time interval as the holotype of *Cardiocorax mukulu* (MGAUN PA103) [43, 44]. MGAUN PA278 was also recovered approximately 250 meters northeast of the holotype of *Cardiocorax mukulu* (MGUAN PA103). Spatial proximity and an overlapping time bin for MGUAN PA103 and MGUAN PA278 reduces the possibility for two morphologically indistinguishable and spatially and temporally contemporaneous but distinct taxa. With these lines of evidence, MGUAN PA278 is referred to *Cardiocorax mukulu*.

### Phylogenetic analysis

#### Analysis 1: Heuristic search of plesiosauria

**Fig 28:**
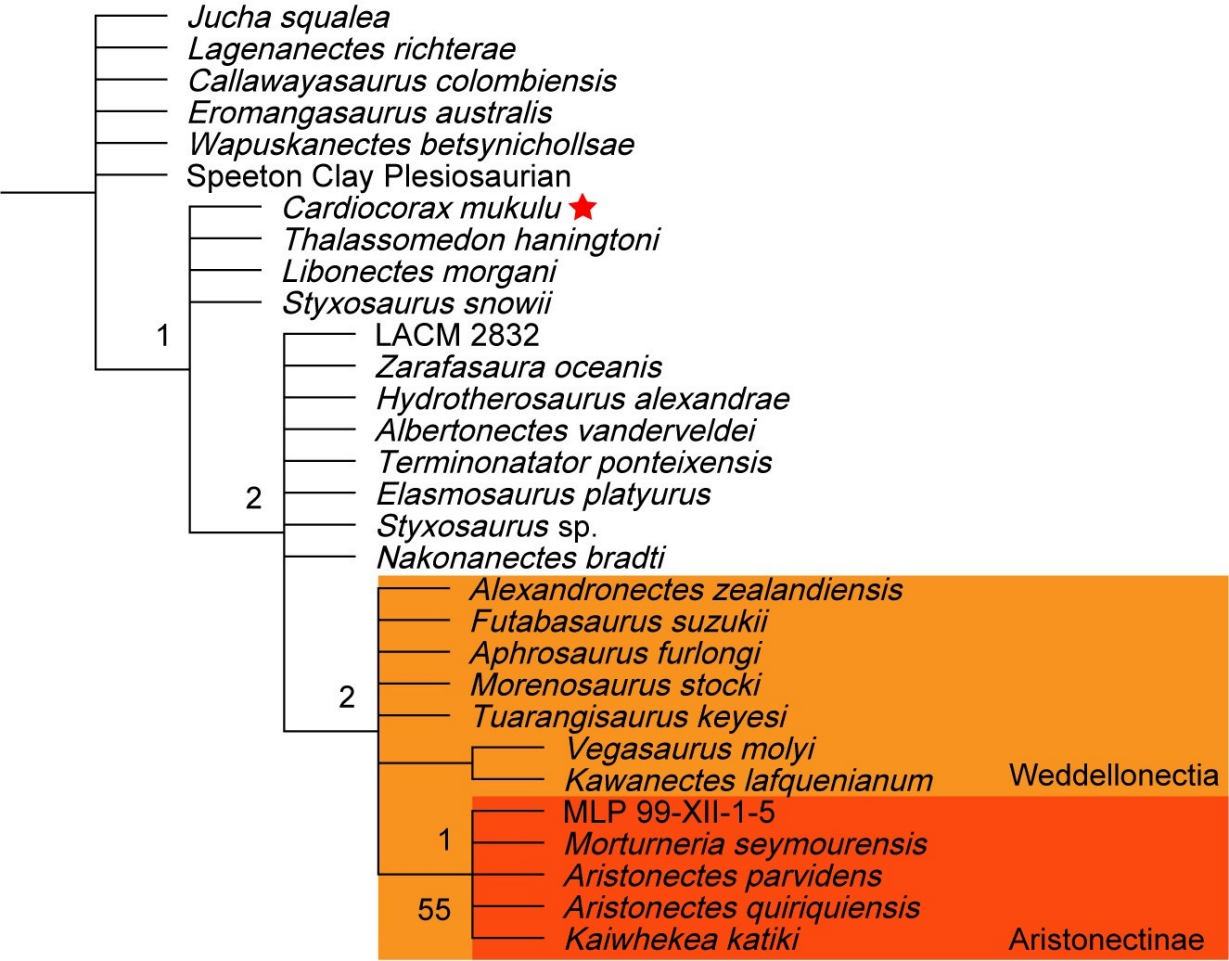
Analysis 1: Phylogenetic relationships of elasmosaurid plesiosaurians from heuristic search of plesiosauria. Bremer scores are displayed above the node. All bootstrap values above 50 are displayed below the node.

Our first phylogenetic analysis is a heuristic search of Plesiosauria (130 taxa) under the following parameters: ‘mxram 1000’, memory set to 100,000 (‘hold 100000’), number of Wagner trees set to 1,000 with 10 trees saved per replication. This heuristic search returned 50 MPTs (CI: 0.224, RI: 0.693; RCI: 0.155; length: 1903) (Fig 28). From the heuristic analysis, *Cardiocorax mukulu* is returned in a polytomy with *Libonectes morgani*, *Thalassomedon haningtoni*, and *Styxosaurus snowii* near the base of Elasmosauridae (Fig 28).

#### Analysis 2: ‘New technology’ search

Our ‘New Technology’ search of Plesiosauria was done in two steps. The first step entailed a ratchet analysis (‘mxram 1000’, ‘hold 100000’, ratchet set to 100 iterations and random addition sequences turned on with 1,000 random addition sequences,) with drift, sectorial search, and tree fusing turned on with default settings. The purpose of this first step is to locate the shortest length tree. This first step yielded 10 MPTs. These trees were then used as the starting point to perform TBR swapping. TBR swapping returned 100,000 trees, with some trees overflowing. The consensus tree after TBR swapping returned nearly the same topology for Elasmosauridae as the heuristic search (CI: 0.225; RI: 0.693; RCI: 0.156; length: 1902). *Cardiocorax mukulu* was returned in the same position as the heuristic search of Plesiosauria (Fig 28).

#### Analysis 3: Implied weighting analysis

**Fig 29:**
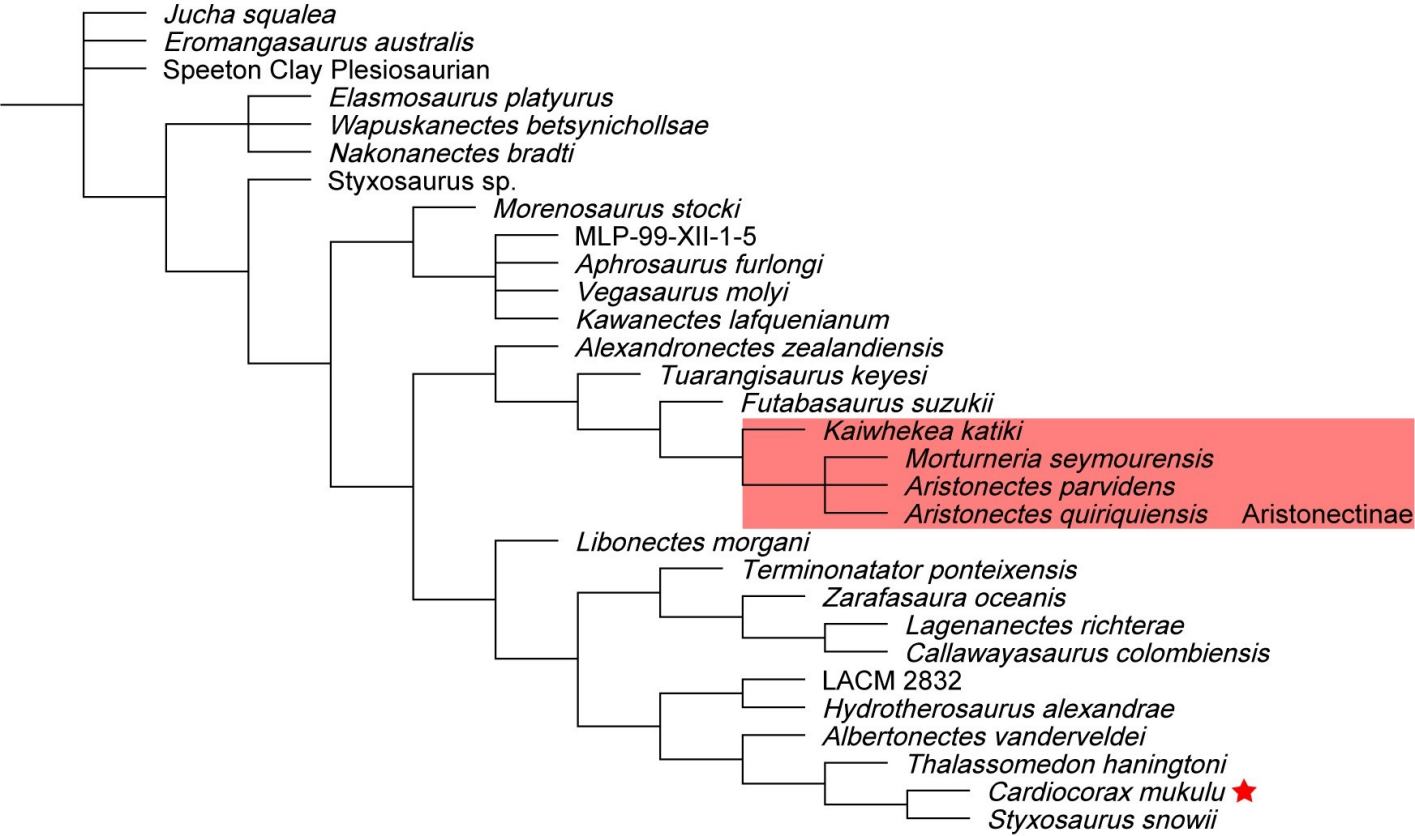
Analysis 3: Consensus tree of elasmosauridae from implied weighting analysis. (K = 3). Symmetric resampling values all below 50.

**Fig 30:**
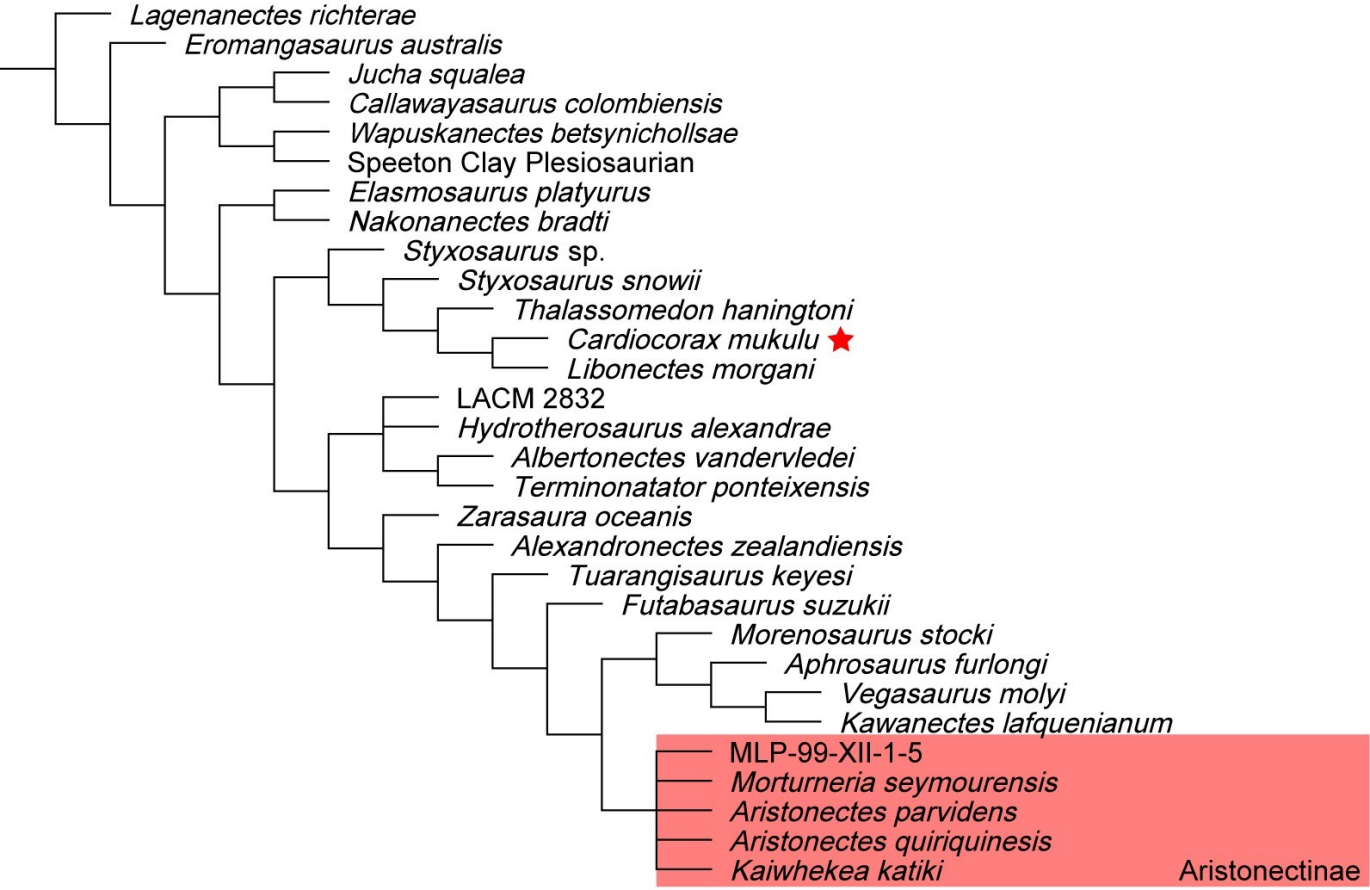
Analysis 3: Consensus tree of Elasmosauridae from implied weighting analysis. (K = 12). Symmetric resampling values were all below 50.

In this analysis set we employed implied weighting with two different analyses using different concavity constants, with extended implied weighting turned on in both (‘xpiwe = ‘). The first analysis used a constant of 3 (‘piwe = 3’) and the second analysis used a constant of 12 (‘piwe = 12’). Both analyses were first run with the same ‘New Technology’ search parameters of analysis 2. After the ‘New Technology’ search, our K = 3 analysis returned 10 MPTs while our K = 12 analysis returned 5 MPTs. After TBR swapping, our K = 3 analysis returned 100,000 trees with overflow and our K = 12 analysis returned 3,696 trees. For the K = 3 analysis, *Cardiocorax mukulu* is returned in the consensus tree as a derived elasmosaurid, and the sister taxon to *Styxosaurus snowii* (CI: 0.218, RI: 0.681, RCI: 0.149, length: 1958) (Fig 29). The second implied weighting analysis (K = 12) returned *Cardiocorax mukulu* as an early-branching taxon and the sister taxon to *Libonectes morgani* (CI: 0.224, RI: 0.692, RCI: 0.155, length: 1909) (Fig 30).

#### Analysis 4: Phylogenetic analysis using Sachs et al. (2021) data matrix

Our last set of analyses uses the recent data matrix of Sachs et al. (2021) [13] to test the phylogenetic position of *Cardiocorax mukulu* with alternative character scores and a different set of taxa. Thus, in this set of analyses we simply included *Cardiocorax mukulu* without altering character scores to other plesiosaur taxa. Two analyses were run, one with K = 3 and the second with K = 12 with the same ‘New Technology’ search parameters used for the TNT analysis in Sachs et al. (2021) [13]: 100 addition sequences and sectorial searches, ratchet, drift, and tree fusing with default settings. TBR was subsequently run after the ‘New Technology’ search with 200,000 trees saved to memory.

**Fig 31:**
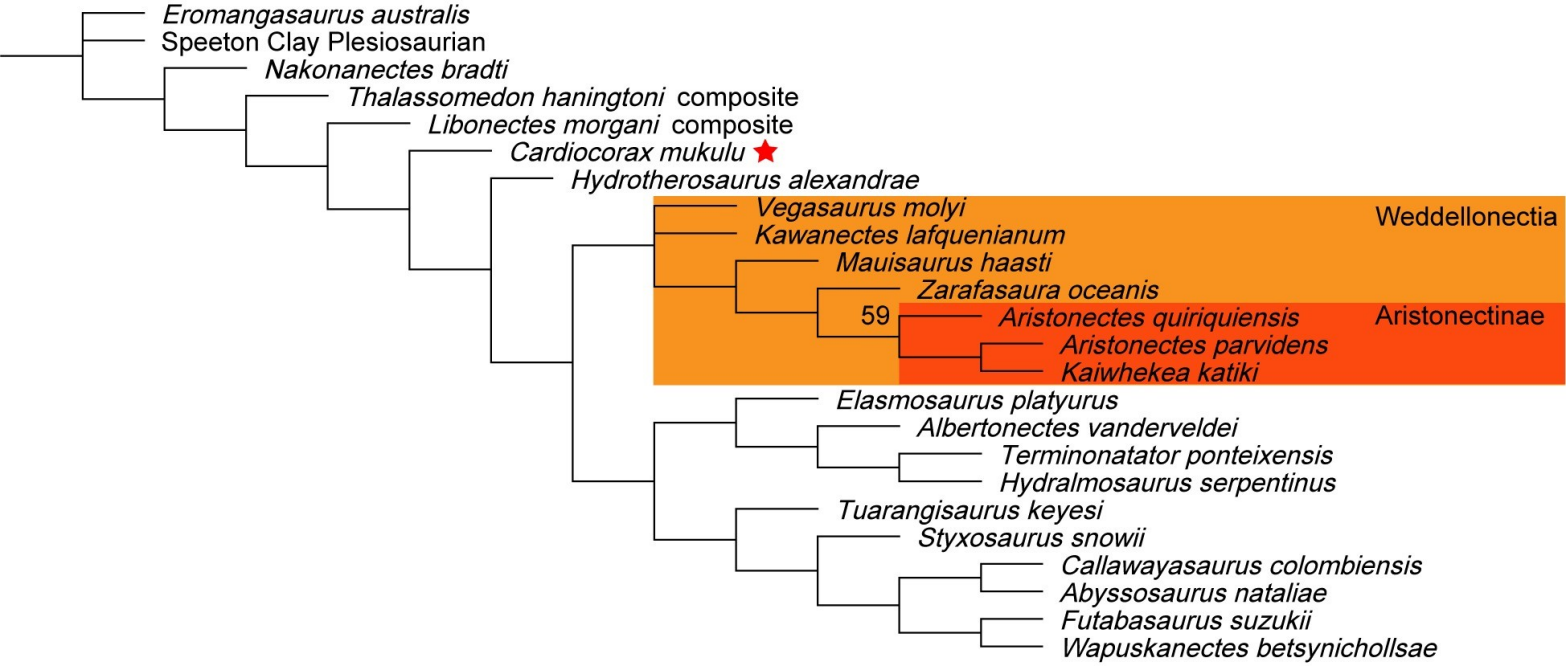
Implied weighting with Sachs et al. (2021) matrix (K = 3). Symmetric resampling values above 50 displayed above nodes.

**Fig 32:**
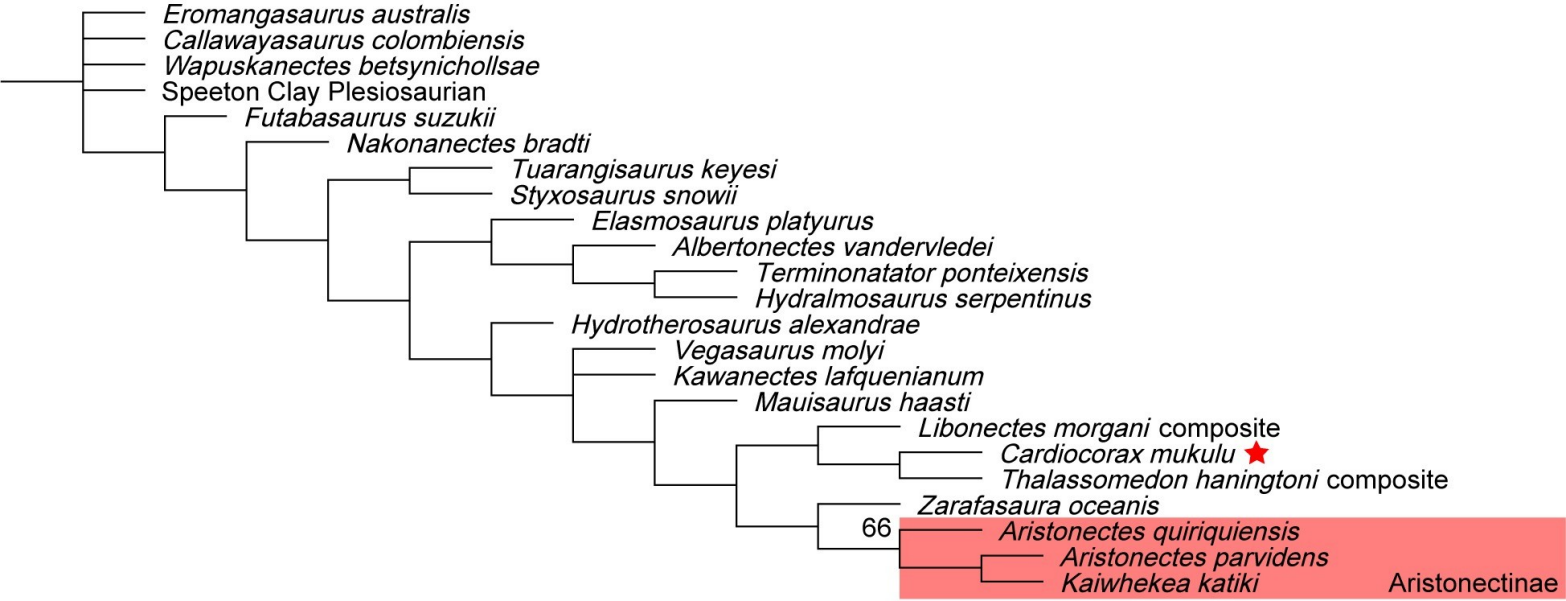
Implied weighting with Sachs et al. (2021) matrix (K = 12). Symmetric resampling values above 50 displayed above nodes.

The K = 3 analysis returned 4 MPTs after the ‘New Technology’ search, with 32,319 trees recovered after TBR (CI: 0.194, RI: 0.679, RCI: 0.131, length: 2080). *Cardiocorax mukulu* is returned in this K = 3 analysis as an early-branching elasmosaurid (Fig 31). The K = 12 analysis returned 26 MPTs after the ‘New Technology’ search, with 46,170 trees recovered after TBR (CI: 0.197, RI: 0.685, RCI: 0.135, length: 2045) and places *Cardiocorax mukulu* as the sister taxon to *Thalassomedon haningtoni* (composite), with *Libonectes morgani* branching outside of this group (Fig 32).

## Discussion

The results from our phylogenetic analyses returned *Cardiocorax mukulu* branching early in the evolution of Elasmosauridae or in an intermediate position in four out of the six analyses. These results are congruent with that of O’Gorman (2020) [3] who recovered *Cardiocorax mukulu* as an early-branching elasmosaurid. *Cardiocorax mukulu* is returned as the sister taxon of *Styxosaurus snowii* in our K = 3 implied weighting analysis with the modified Fischer et al. (2020) [12] data matrix. This result is in agreement with the results of Araújo et al. (2015) [26] and Fischer et al. (2020) [12] in which *Cardiocorax mukulu* is returned in a derived clade with *Hydrotherosaurus alexandrae*, *Terminonatator ponteixensis*, *Albertonectes vanderveldei*, and LACM 2832. The phylogenetic analyses of Araújo et al. (2015) [26], O’Gorman (2020) [3], and Fischer et al. (2020) [12] relied upon the scoring provided by the holotype of *Cardiocorax mukulu*, which until now only included postcranial material (Araújo et al., 2015) [26]. The consensus among our analyses suggests that *Cardiocorax mukulu* likely represents an early or intermediary lineage of elasmosaurids that persisted into the Maastrichtian.

*Cardiocorax mukulu* also lacks the elongated cervical vertebrae that characterizes elasmosaurines [7] and instead exhibits the condition where in most vertebrae W:L is at least 1.5 (Table 2). It is however possible that this character may have been reversed, if *Cardiocorax mukulu* is closely related to elasmosaurines. *Nakonanectes bradti* for example was returned with styxosaurines despite lacking elongated cervical vertebrae by Serratos et al. (2017) [41]. Although there is conflict regarding the phylogenetic position of *Nakonanectes bradti*, Fischer at al. (2020) [12] recovered *Nakonanectes bradti* in a basal polytomy within Elasmosauridae, and Sachs et al. (2021) [13] returned *Nakonanectes bradti* often forming a clade with *Thalassomedon haningtoni* and *Libonectes morgani* [12], while O’Gorman (2020) [3] recovered *Nakonanectes bradti* within Elasmosaurinae. Aristonectine plesiosaurs provide a second example of centrum length reduction within Elasmosauridae [12]. Although *Cardiocorax mukulu* lacks other diagnostic traits of aristonectines [3, 75] and is not recovered within Aristonectinae in any of our phylogenetic analyses. In our implied weighting analysis (K = 12) using the matrix of Sachs et al. (2021) [13] (Fig 32), *Cardiocorax mukulu* technically falls within the clade Weddellonectia according to the definition outlined by O’Gorman (2020) [3]. However, in the consensus tree *Cardiocorax mukulu* forms a clade with the Cenomanian-Turonian aged *Libonectes morgani*, which has unambiguously been regarded as early-branching or intermediary elasmosaurid in recent phylogenetic analyses [3, 7, 13, 42, 53, 76, 101], and *Thalassomedon haningtoni* which from the results of Sachs et al. (2021) [13] was demonstrated to also be an early-branching elasmosaurid taxon.

The weakly supported phylogenies in our analyses (low consistency and retention indices, as well as low symmetric resampling values) indicate significant homoplasy within Elasmosauridae, which can lead to various topologies with little consistency in structure. This is an observation that has been recounted multiple times by previous studies [12, 13, 41, 42]. The only consistent clade recovered in our analyses is Aristonectinae, which are a highly derived group of elasmosaurids characterized by high tooth counts, shortened cervical vertebrae, and a unique cranial morphology [68]. As mentioned by Serratos et al. (2017) the reasons for this issue could include the use of different character matrices, taxon sampling, and a lack of descriptive data [41]. In our review of character scores for specimens studied in-person, we found 113 of our scores differed from that of work done by Sachs et al. (2017) [53], Sachs et al. (2018) [42], and Sachs et al. (2021) [13]. The implications of this are simply that morphological character scores should be checked from those of previous analyses in an effort to produce the most accurate matrices possible for phylogenetic analysis. Along with this, any character that is modified should be accompanied with an argument for the character change. This effort to improve character scores with accompanied arguments for each character change was previously done by Serratos et al., (2017) [41] for *Libonectes morgani*. The scores provided by published work on revised descriptions and character scores for *Libonectes morgani*, and *Styxosaurus snowii* by Sachs et al. (2017) [53] and Sachs et al. (2018) [42] were not incorporated into the matrix of Fischer et al. (2020) [12], which in turn was based on O’Gorman (2020) [3]. We strongly suggest that updated character scoring must be incorporated into data matrices to keep up with the rate of revised data. We also suggest providing arguments for introducing character scores that are modified from that of the parent matrix. Despite the updated character scoring provided in our study, our results suggest that a more detailed level of character analysis may be required to resolve the evolutionary relationships of elasmosaurids outside of Aristonectinae and improve resolution.

## Conclusion

The new partial plesiosaur skeleton, MGUAN PA278, which includes the most complete plesiosaur skull from sub-Saharan Africa, preserves a nearly complete skull along with postcranial elements. The new skull of *Cardiocorax mukulu* is significant in that the three-dimensional preservation of most of the skull remains intact. This new specimen is identified as *Cardiocorax mukulu* because overlapping skeletal material is morphologically indistinguishable from the holotype, and it shares with the holotype an apomorphy where the cervical neural spines are approximately the same length as the centra and exhibit a sinusoidal anterior margin. The new specimen allows an updated diagnosis based on a combination of characters from the skull, the cervical vertebrae, and the pectoral girdle. Six phylogenetic analyses were run to test the position of *Cardiocorax mukulu* within Plesiosauria, this included equal weighting and implied weighting analyses. Most of these analyses suggest an early-branching or intermediate position for *Cardiocorax mukulu* within Elasmosauridae. The lack of elongated cervical vertebrae, which is a characteristic of elasmosaurines, is absent in *Cardiocorax mukulu*. Our results indicate several lineages of elasmosaurids persisted into the Maastrichtian.

### Repository information for studied specimens

MGUAN PA103:Museu da Lourinhã, Lourinhã, Portugal.
MGUAN PA270: Southern Methodist University, Dallas, Texas, U.S.A.
MGUAN PA278: Universidade Nova de Lisboa, Faculdade de Ciências e Tecnologia, Caparica, Portugal.
KUVP 1301: University of Kansas, Vertebrate Paleontology, Museum of Natural History, Lawrence, Kansas, U.S.A.
UNSM 50132: University of Nebraska State Museum, Lincoln, Nebraska, U.S.A.
SMU SMP 69120: Southern Methodist University, Dallas, Texas, U.S.A.
UCMP 38349: University of California Museum of Paleontology, Berkeley, California, U.S.A.

## Supporting information

S1 Fig Tooth count of *Styxosaurus snowii* (KUVP 1301) on the right side of the skull. (TIF)

S2 Fig Interpretation of *Thalassomedon haningtoni* (UNSM 50132) cranial and mandibular anatomy on the left side of the skull. Dashed lines indicate approximate sutural contacts. Postfrontal is indicated by pf. Photo of UNSM 50132 courtesy of Elliot Armour Smith. Carpenter (1999) was used as a reference to interpret bone sutures.
(TIF)

S3 Fig Interpretation of *Styxosaurus snowii* (KUVP 1301) cranial and mandibular anatomy on the left side of the skull. Dashed lines indicate approximate boundaries or sutural contacts. Skull interpretations from Carpenter (1999) and Sachs et al. (2018) were used as references to interpret bone sutures.
(TIF)

S4 Fig Interpretations of *Styxosaurus snowii* (KUVP 1301) cranial and mandibular anatomy on the right side of the skull. Dashed lines indicate approximate sutural contacts. Skull interpretations from Carpenter (1999) and Sachs et al. (2018) were used as references to interpret bone sutures.
(TIF)

S5 Fig Dorsal view of *Libonectes morgani* (SMU SMP 69120) with character scores indicated. (TIF)

S6 Fig View of occiput of *Libonectes morgani* (SMU SMP 69120) with character scores indicated. (TIF)

S7 Fig Anterior end of the palate of *Libonectes morgani* (SMU SMP 69120) with character scores indicated. (TIF)

S8 Fig Oblique view of quadrate ramus of the pterygoid indicating the absence of a lappet. Interpretation is based on a high-resolution cast of *Libonectes morgani* (SMU SMP 69120) based on CT data.
(TIF)

S9 Fig Posterior portion of the palate from *Libonectes morgani* (SMU SMP 69120) showing character scores. (TIF)

S10 Fig The suture between the prefrontal and the maxilla is identified in KUVP 1301. (TIF)

S11 Fig *Styxosaurus snowii* (KVUP 1301) character 110 score. (TIF)

S12 Fig *Styxosaurus snowii* (KVUP 1301) character 114 score. (TIF)

S13 Fig *Styxosaurus snowii* (KVUP 1301) character 143 score. (TIF)

S14 Fig *Styxosaurus snowii* (KVUP 1301) character 145, 146, and 147 scores. (TIF)

S15 Fig *Styxosaurus snowii* (KVUP 1301) scores for characters 157, 163, 171, 172, and 175. (TIF)

S16 Fig *Thalassomedon haningtoni* (UNSM 50132) scores for characters 18 and 44. (TIF)

S17 Fig *Thalassomedon haningtoni* (UNSM 50132) scores for characters 48 and 58. (TIF)

S18 Fig *Thalassomedon haningtoni* (UNSM 50132) scores for characters 158 and 159. (TIF)

S19 Fig *Thalassomedon haningtoni* (UNSM 50132) score for character 165 state 1. (TIF)

S20 Fig *Thalassomedon haningtoni* (UNSM 50132) character 165 state 0. (TIF)

S21 Fig *Thalassomedon haningtoni* (UNSM 50132) character 172 score. (TIF)

S22 Fig *Callawayasaurus colombiensis* (UCMP 38349) in dorsal view with character scores indicated. (TIF)

S23 Fig *Callawayasaurus colombiensis* (UCMP 38349) in right lateral view with character scores indicated. (TIF)

S24 Fig *Callawayasaurus colombiensis* (UCMP 38349) with occiput in view and character scores indicated. (TIF)

S25 Fig *Callawayasaurus colombiensis* (UCMP 38349) in ventral view. (TIF)

S26 Fig Scapula, clavicle, and interclavicle of *Cardiocorax mukulu* holotype (MGUAN PA103). Abbreviations: cl., clavicle; icl., interclavicle; sc., scapula.
(TIF)

S27 Fig Pectoral girdle of *Cardiocorax mukulu* holotype (MGUAN PA103). Intercoracoid fenestra is indicated by icf.
(TIF)

S28 Fig Consensus tree of heuristic search of Plesiosauria with the modified matrix of Fischer et al. (2020). (TIF)

S1 File (DOCX)
