# Supplementary figures and images for: The cranial anatomy and relationships of Cardiocorax mukulu (Plesiosauria: Elasmosauridae) from Bentiaba, Angola
Source: PLoS One. 2021 Aug 17;16(8):e0255773. doi: 10.1371/journal.pone.0255773 (PMC8370651; doi:10.1371/journal.pone.0255773)

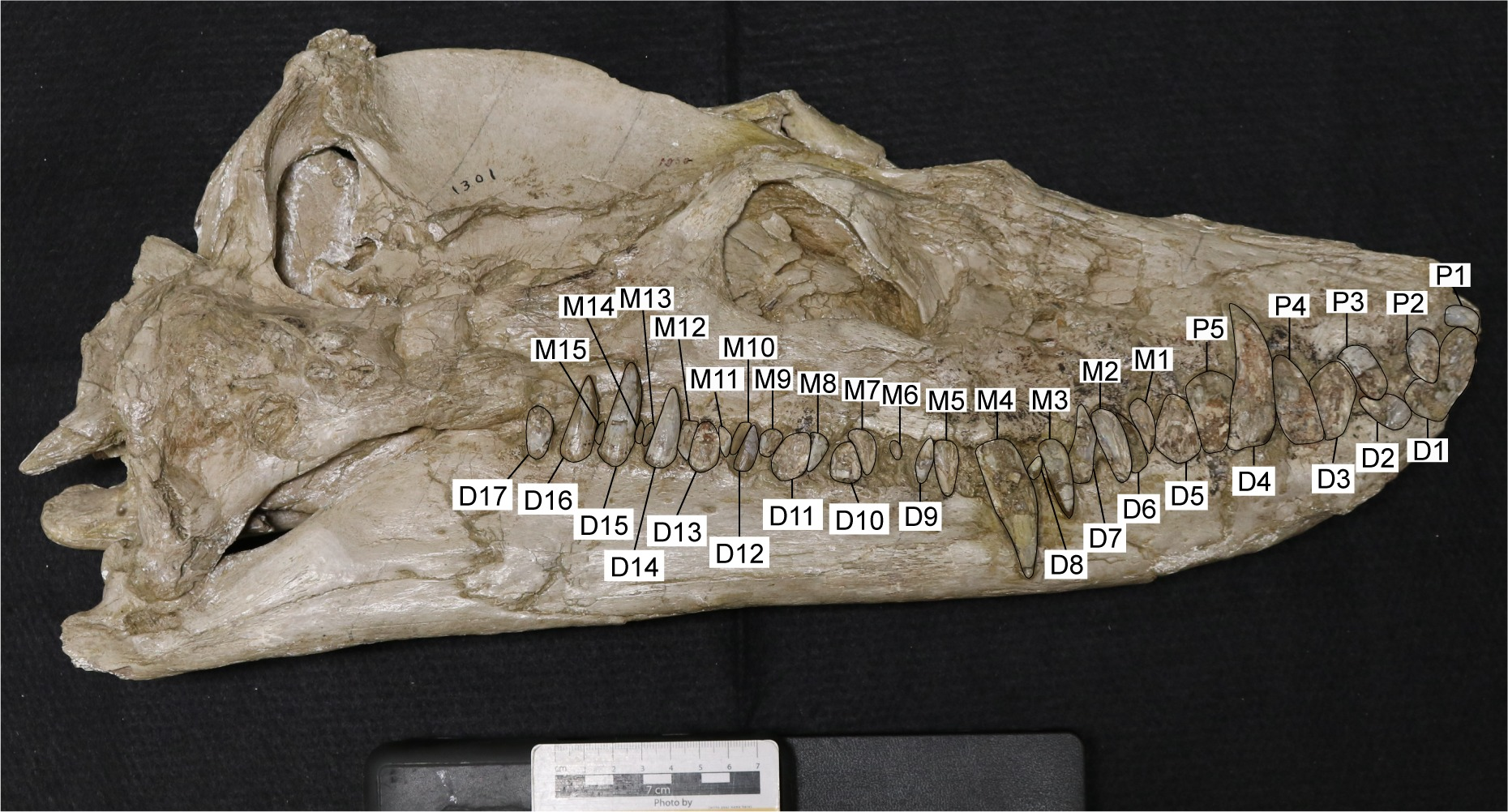

Supplement: S1 Fig — (TIF) [file pone.0255773.s001.tif]

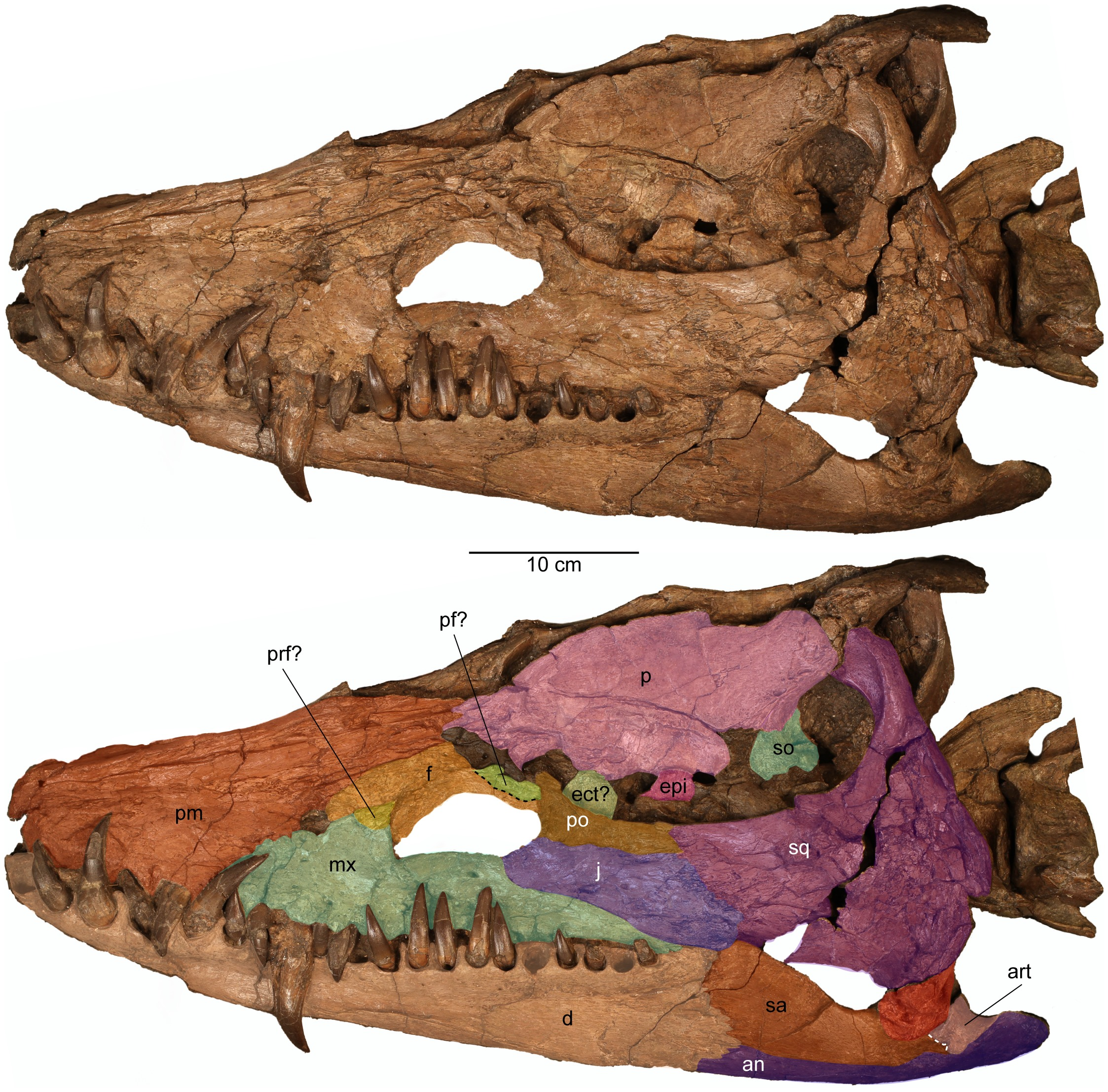

Supplement: S2 Fig — Dashed lines indicate approximate sutural contacts. Postfrontal is indicated by pf. Photo of UNSM 50132 courtesy of Elliot Armour Smith. Carpenter (1999) was used as a reference to interpret bone sutures. (TIF) [file pone.0255773.s002.tif]

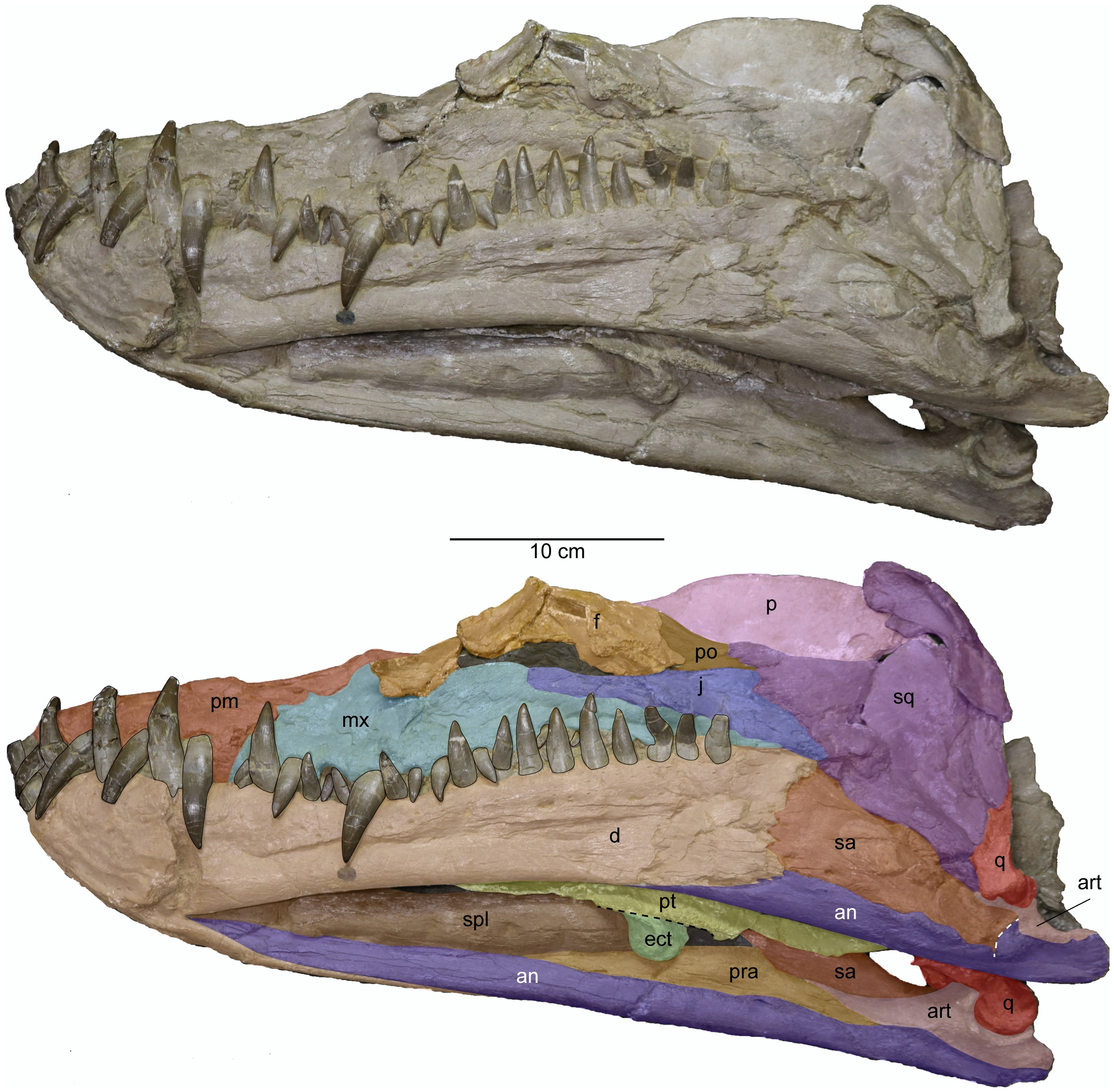

Supplement: S3 Fig — Dashed lines indicate approximate boundaries or sutural contacts. Skull interpretations from Carpenter (1999) and Sachs et al. (2018) were used as references to interpret bone sutures. (TIF) [file pone.0255773.s003.tif]

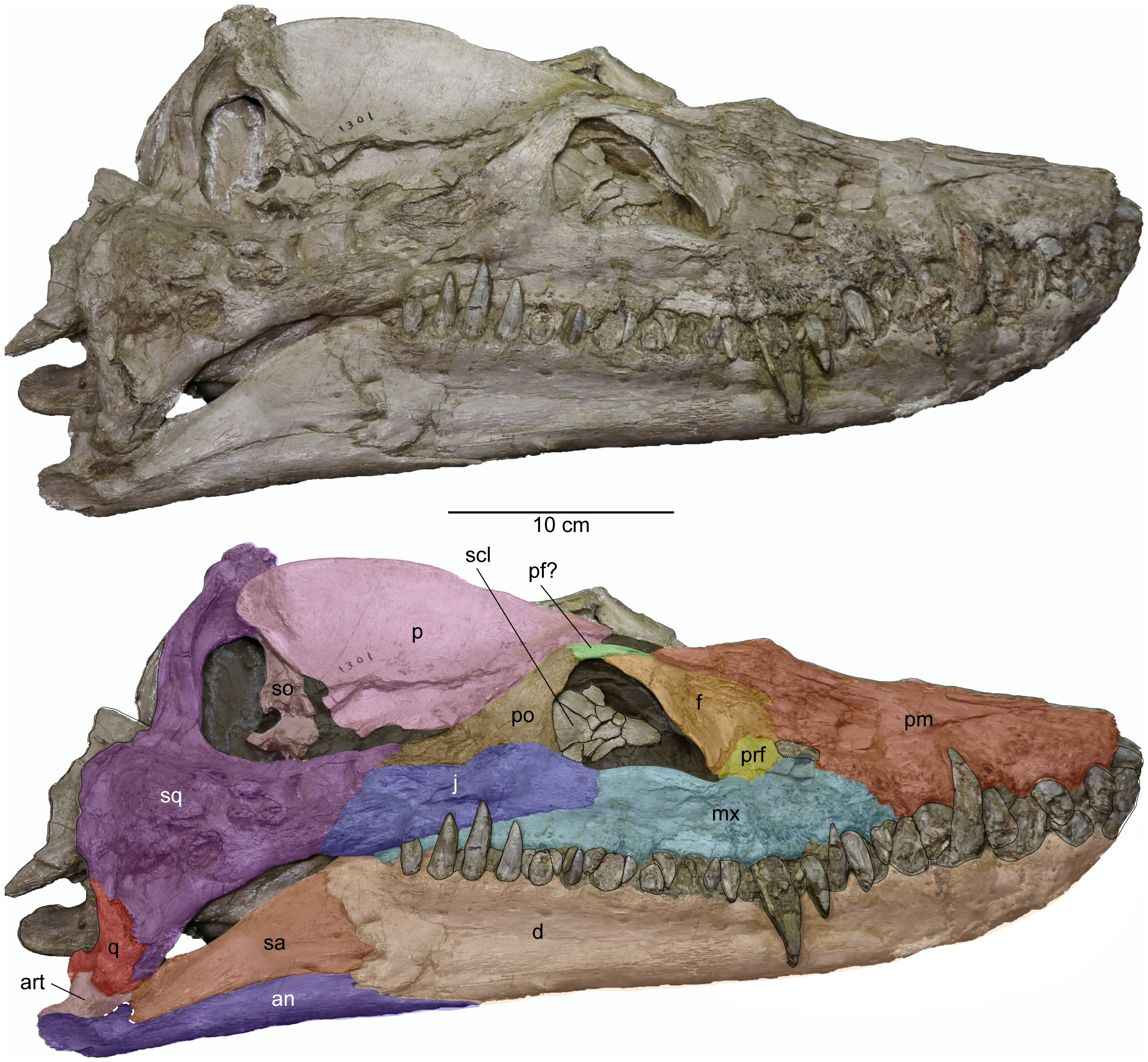

Supplement: S4 Fig — Dashed lines indicate approximate sutural contacts. Skull interpretations from Carpenter (1999) and Sachs et al. (2018) were used as references to interpret bone sutures. (TIF) [file pone.0255773.s004.tif]

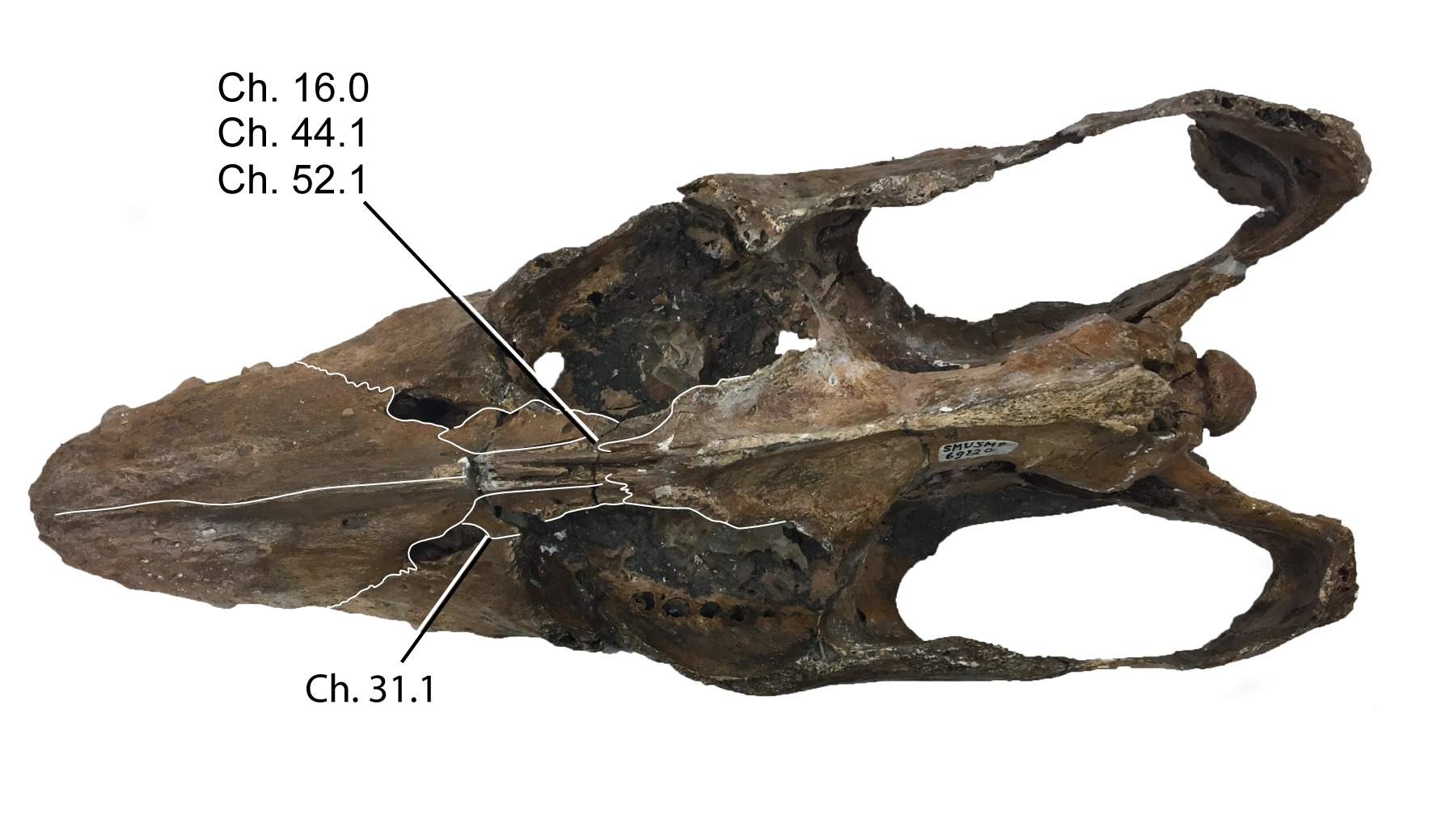

Supplement: S5 Fig — (TIF) [file pone.0255773.s005.tif]

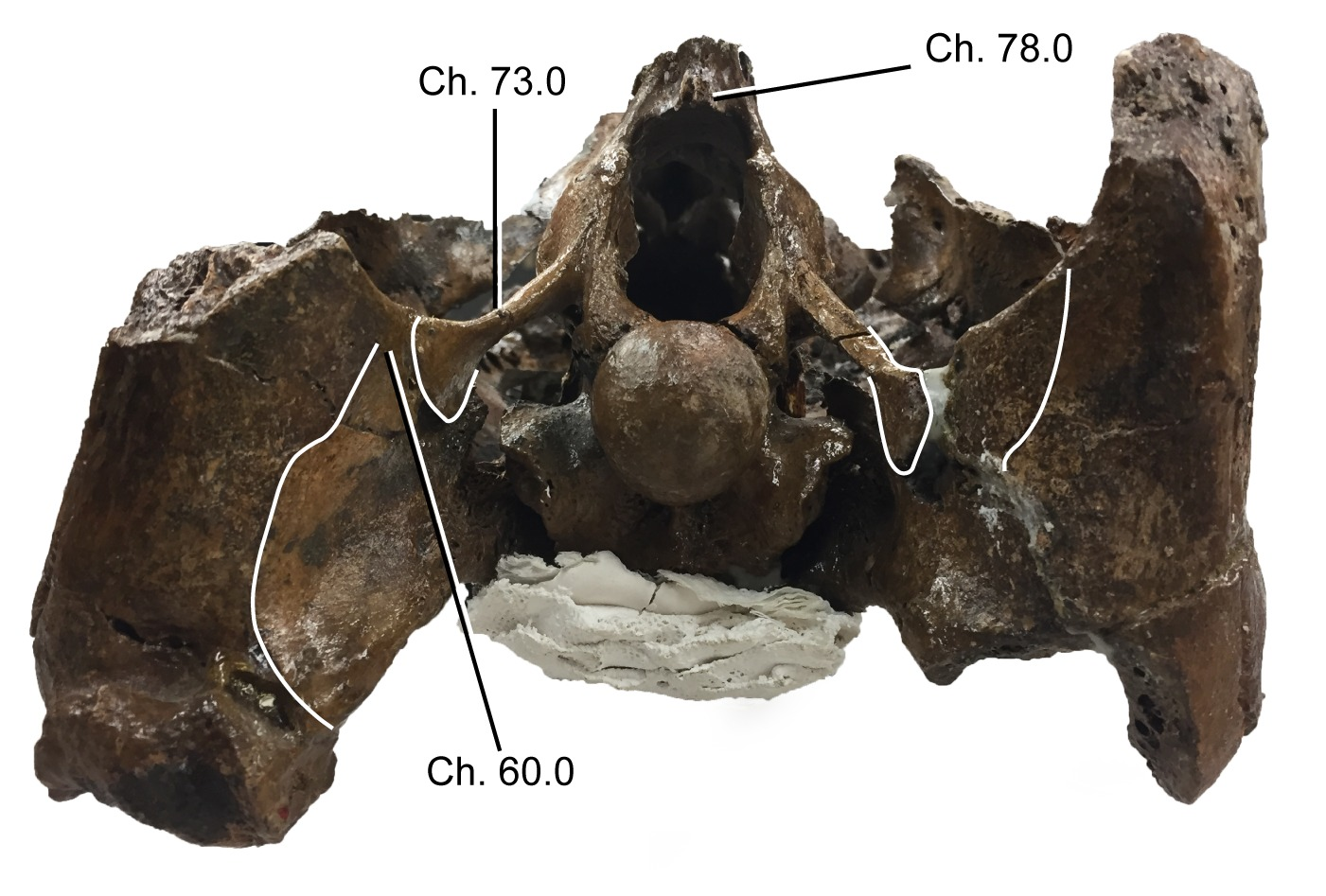

Supplement: S6 Fig — (TIF) [file pone.0255773.s006.tif]

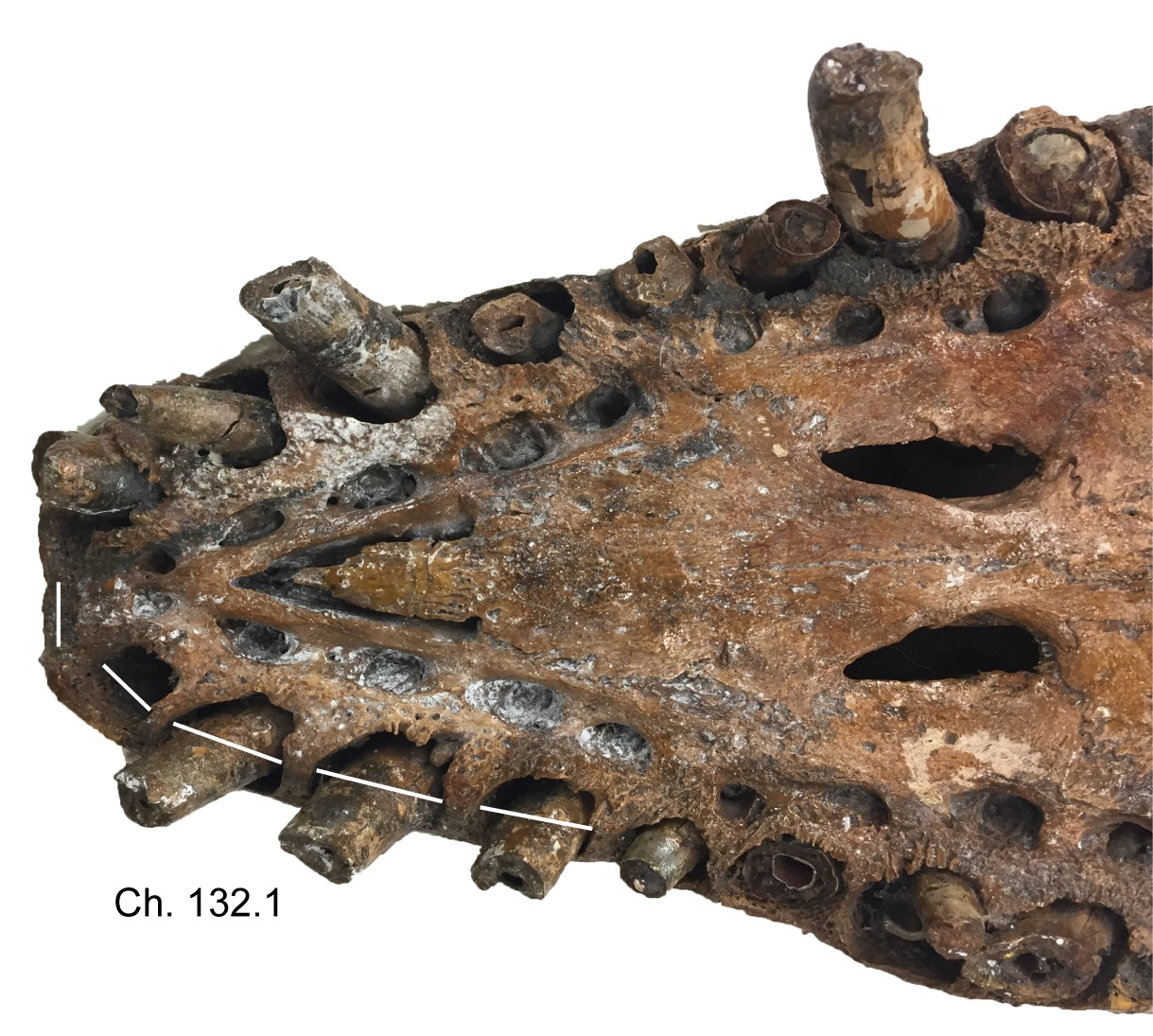

Supplement: S7 Fig — (TIF) [file pone.0255773.s007.tif]

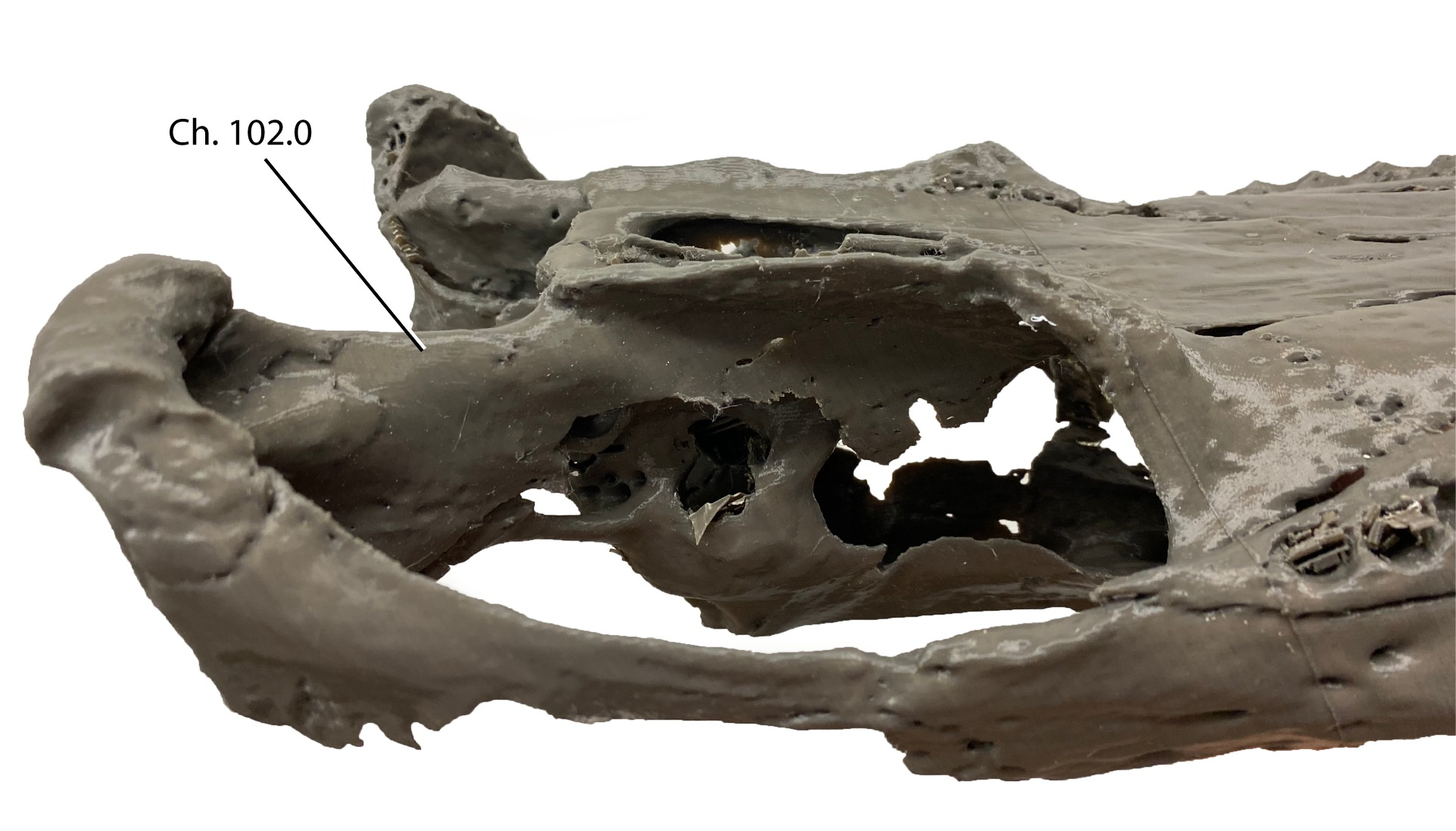

Supplement: S8 Fig — Interpretation is based on a high-resolution cast of Libonectes morgani (SMU SMP 69120) based on CT data. (TIF) [file pone.0255773.s008.tif]

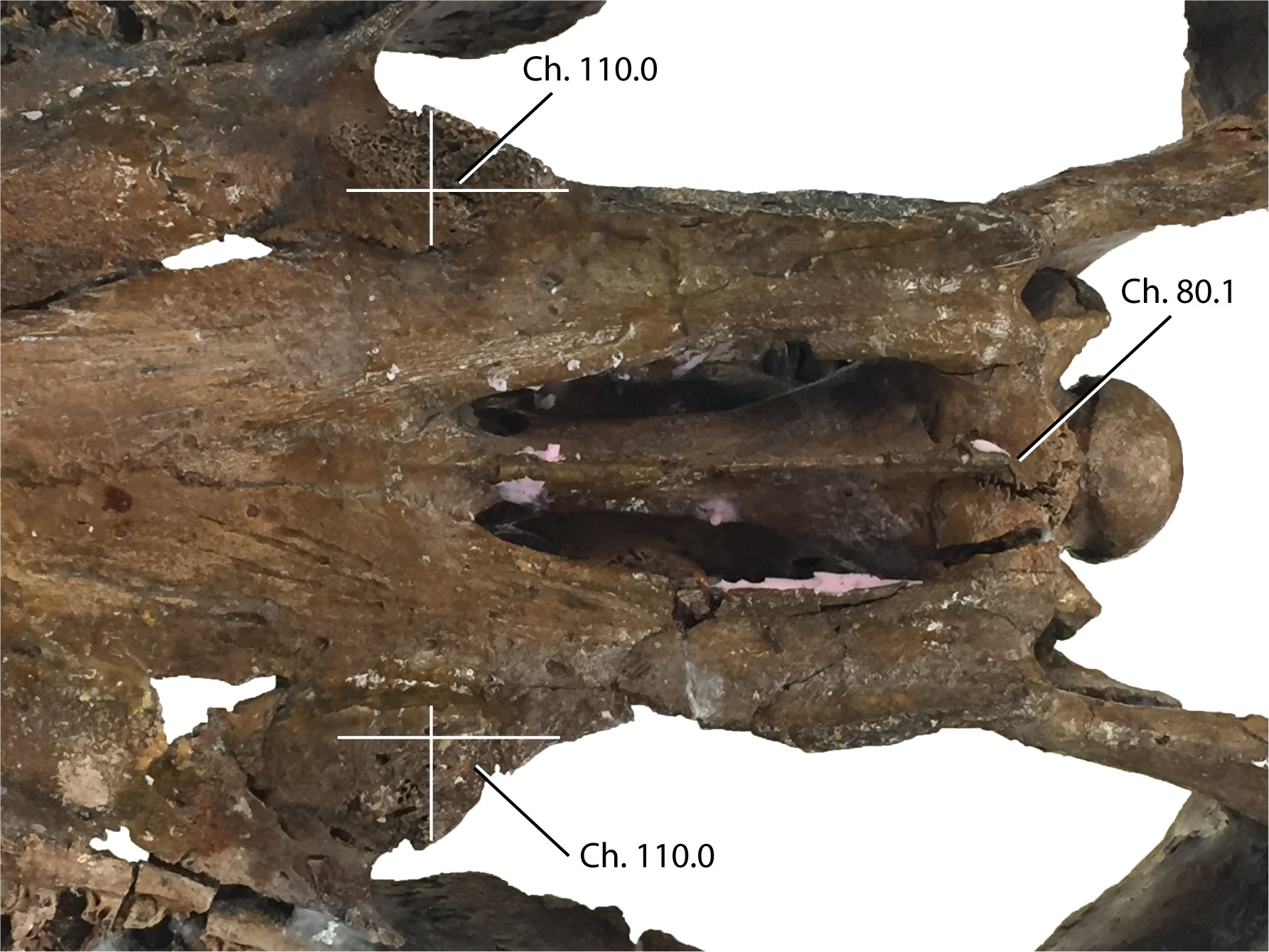

Supplement: S9 Fig — (TIF) [file pone.0255773.s009.tif]

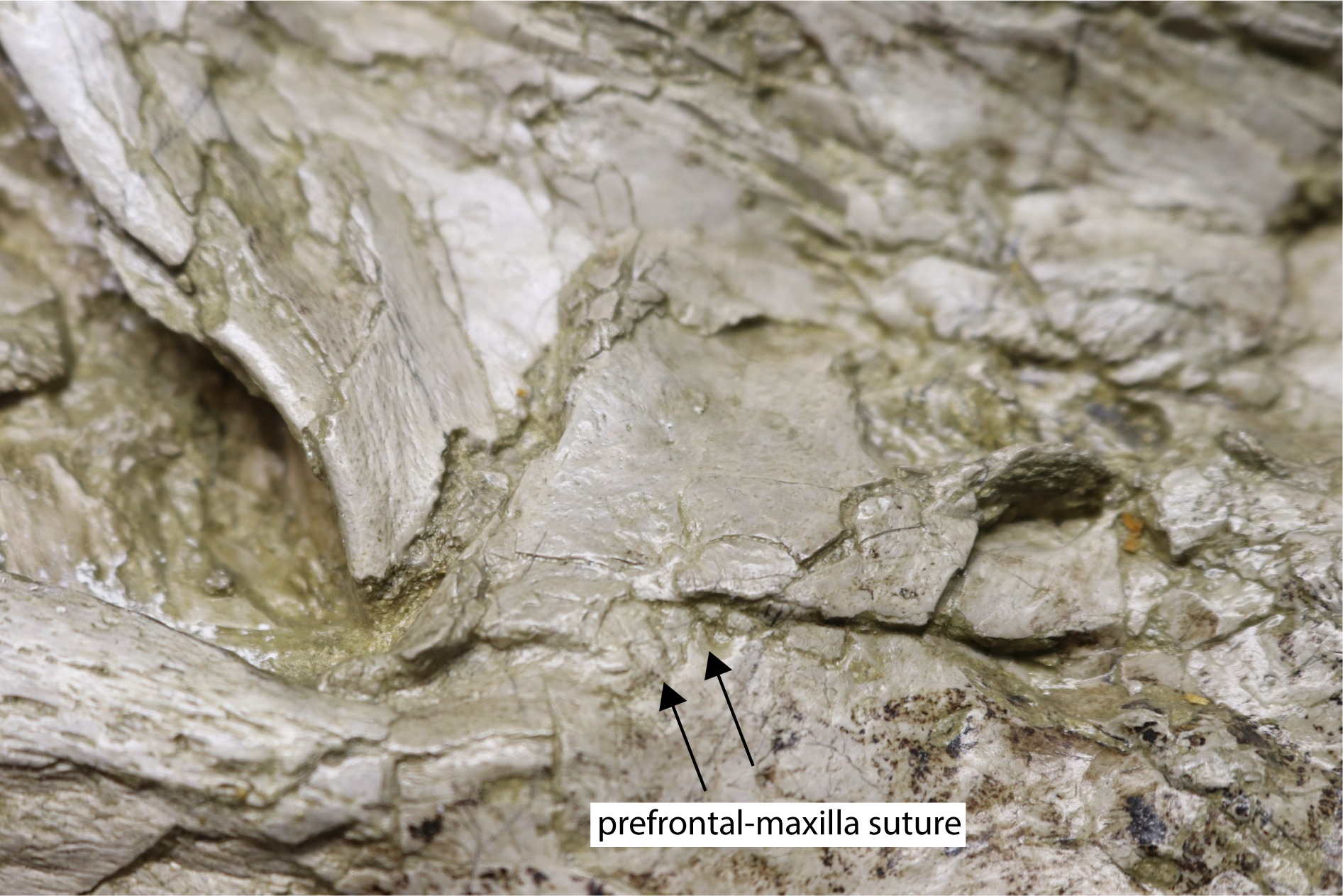

Supplement: S10 Fig — (TIF) [file pone.0255773.s010.tif]

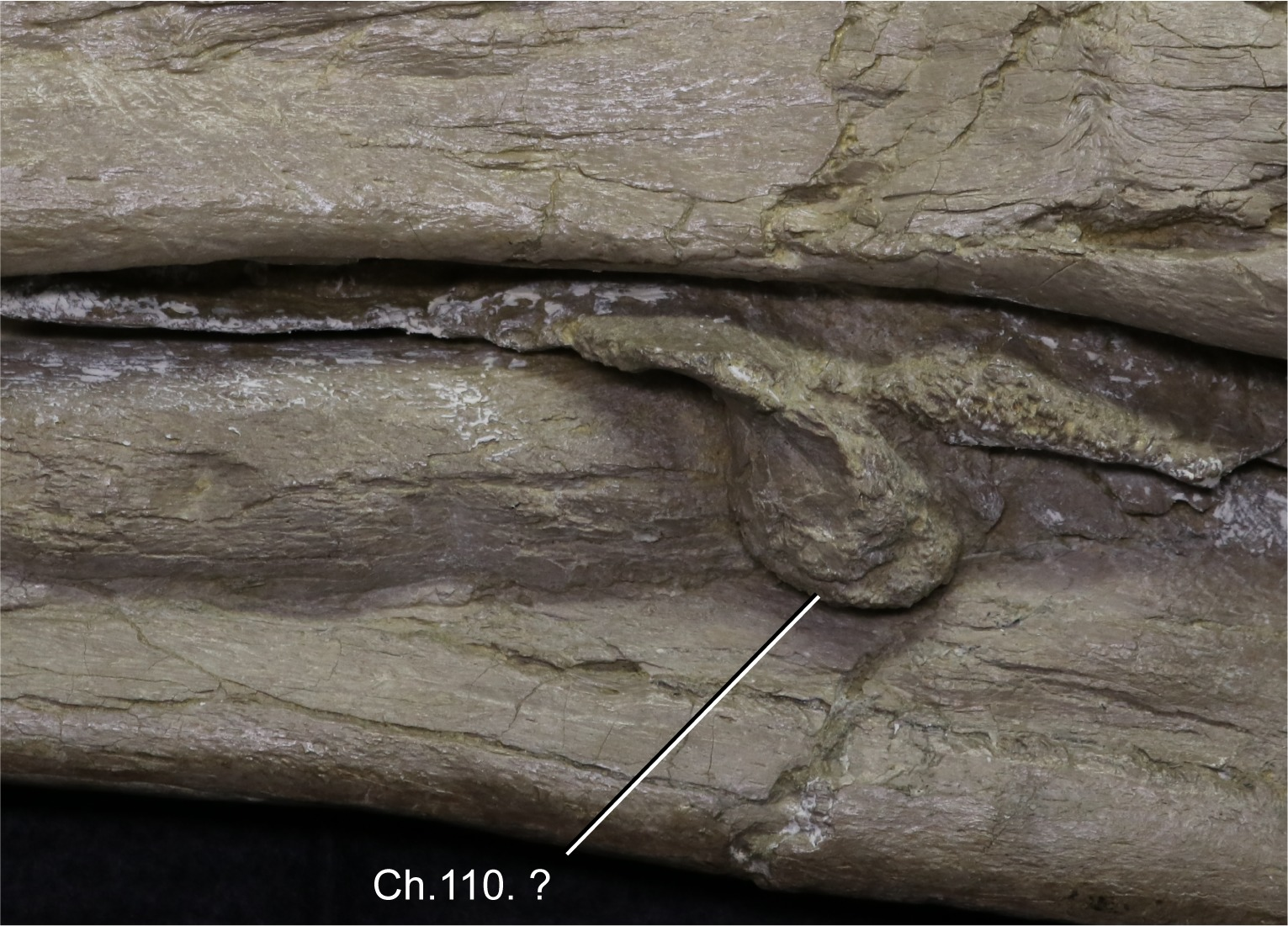

Supplement: S11 Fig — (TIF) [file pone.0255773.s011.tif]

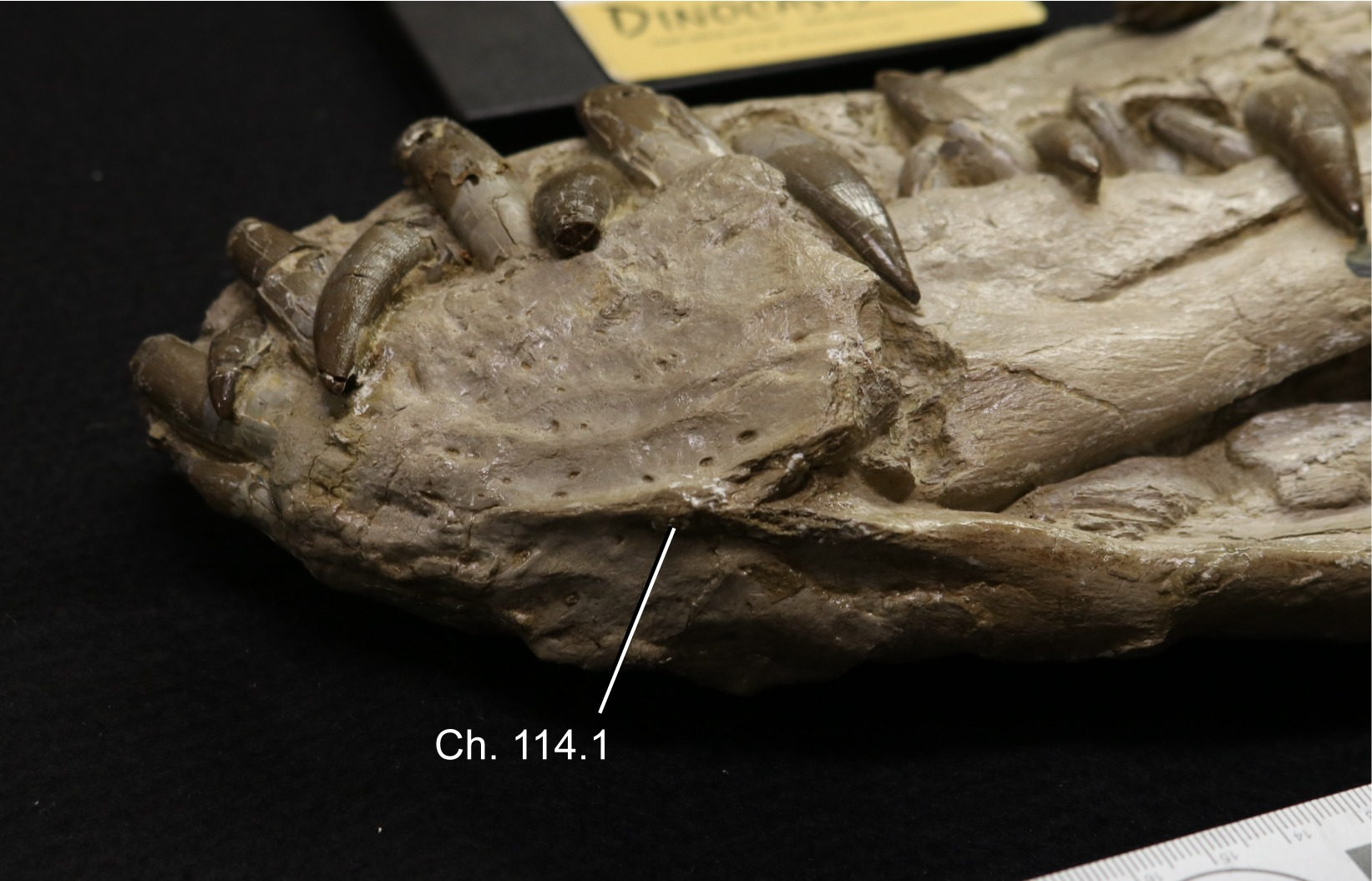

Supplement: S12 Fig — (TIF) [file pone.0255773.s012.tif]

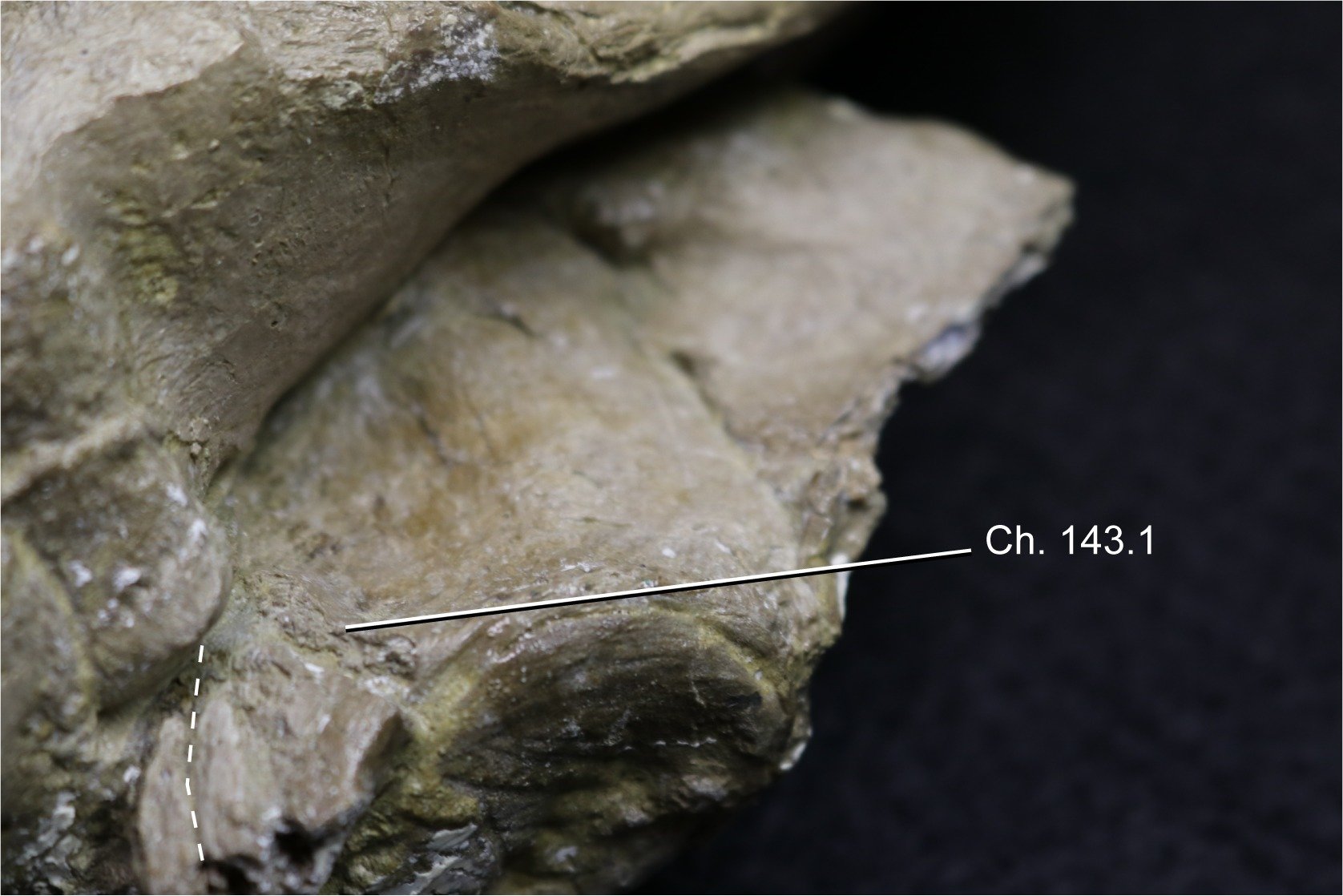

Supplement: S13 Fig — (TIF) [file pone.0255773.s013.tif]

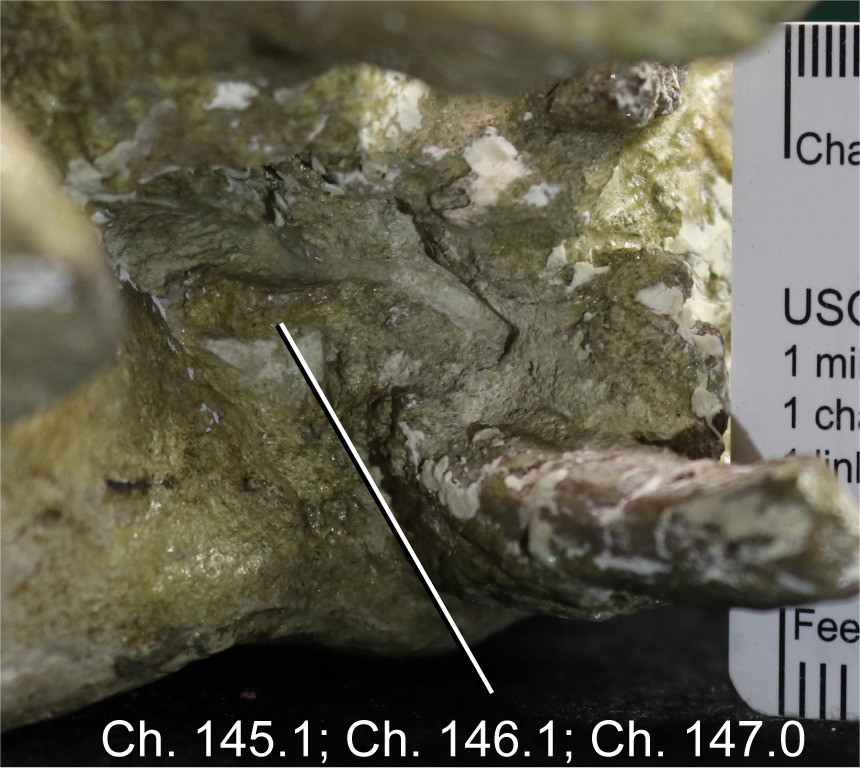

Supplement: S14 Fig — (TIF) [file pone.0255773.s014.tif]

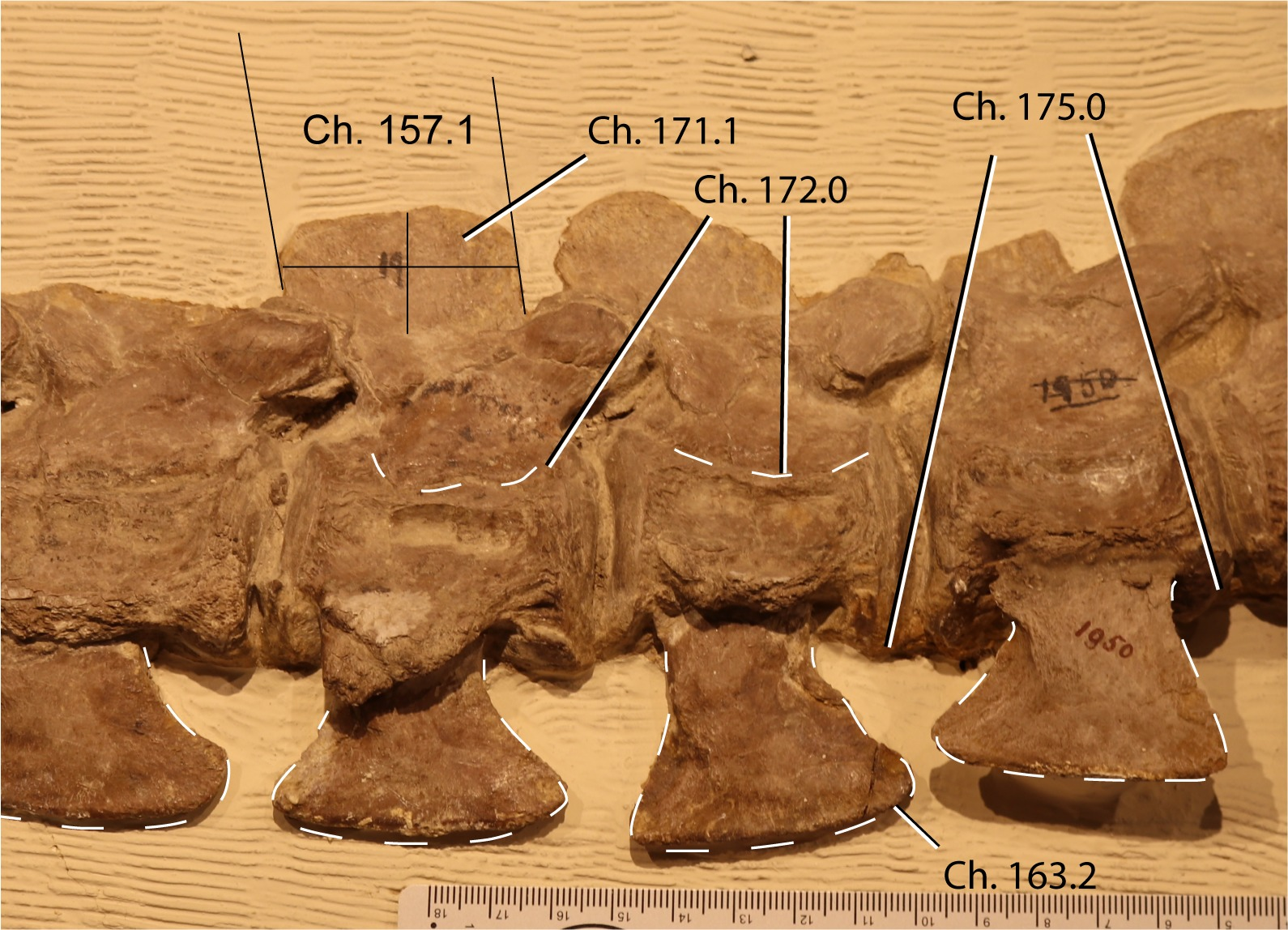

Supplement: S15 Fig — (TIF) [file pone.0255773.s015.tif]

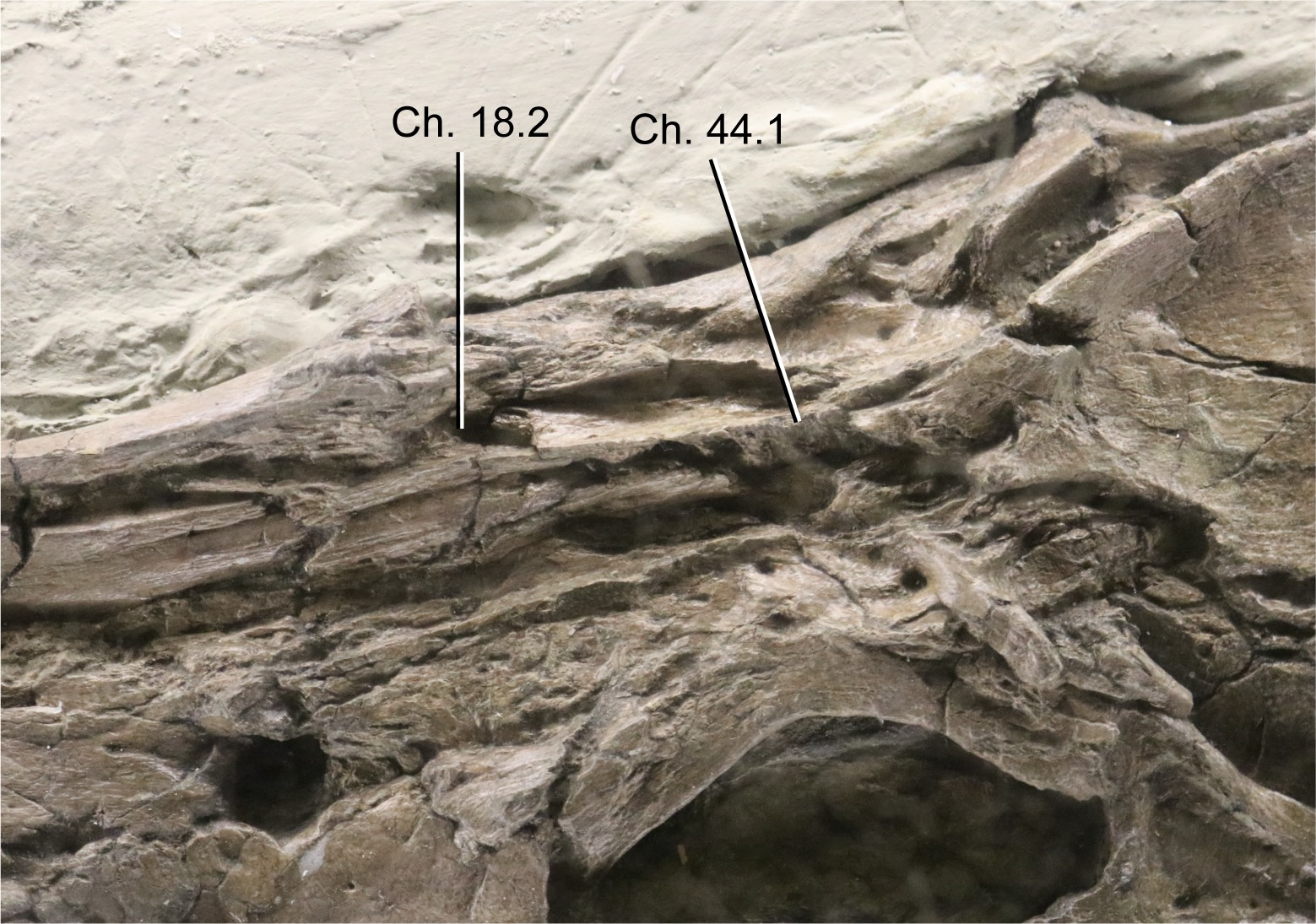

Supplement: S16 Fig — (TIF) [file pone.0255773.s016.tif]

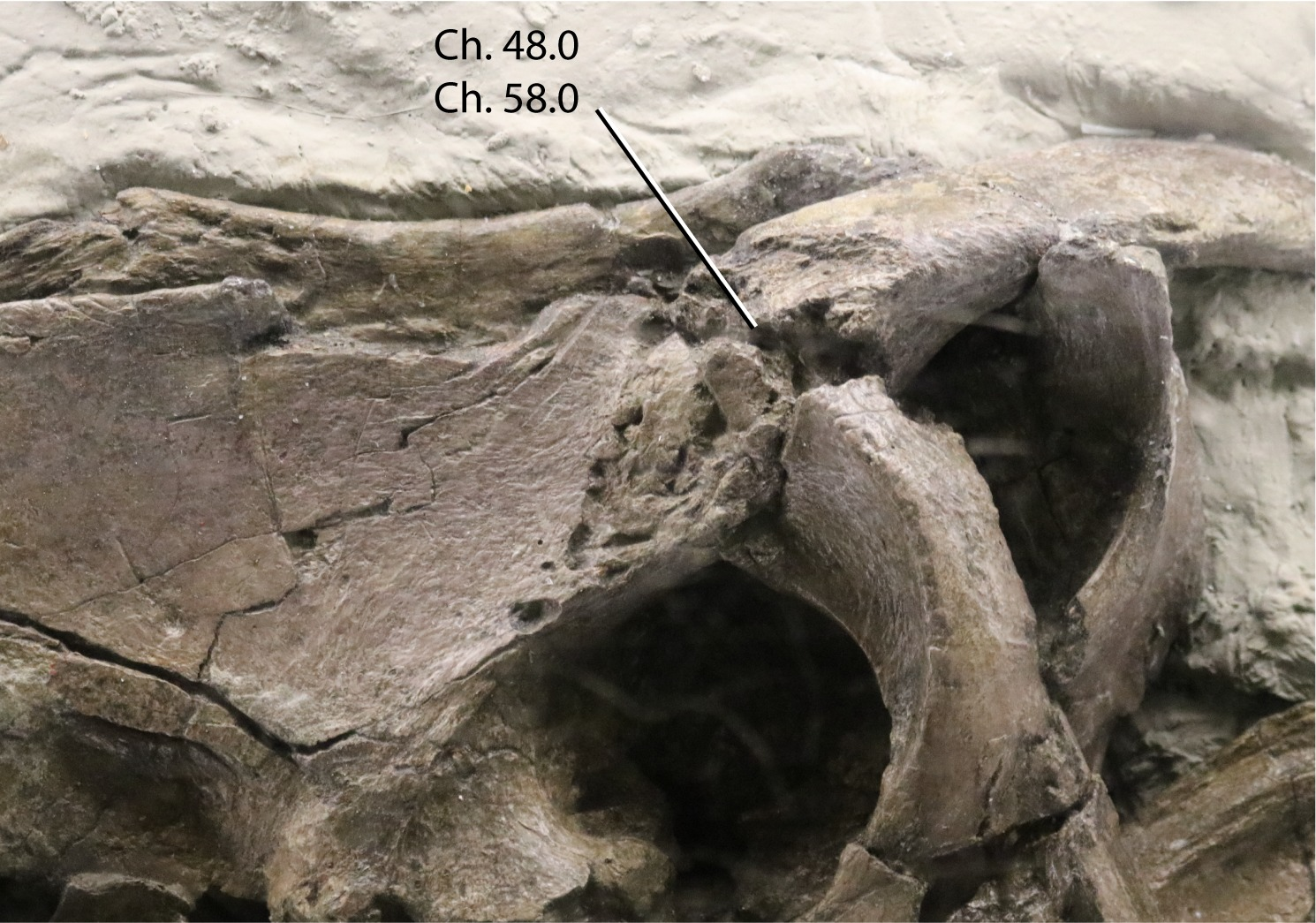

Supplement: S17 Fig — (TIF) [file pone.0255773.s017.tif]

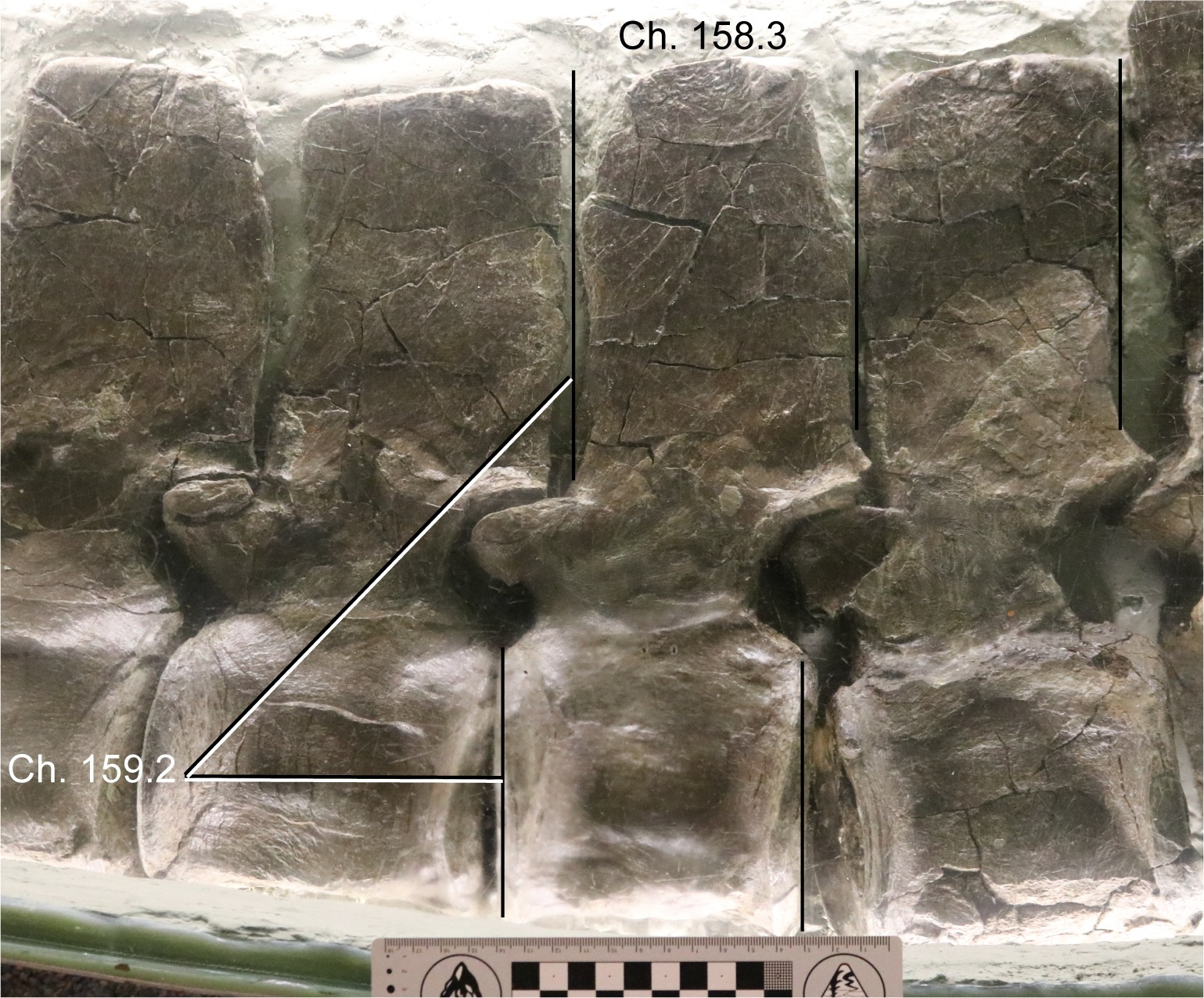

Supplement: S18 Fig — (TIF) [file pone.0255773.s018.tif]

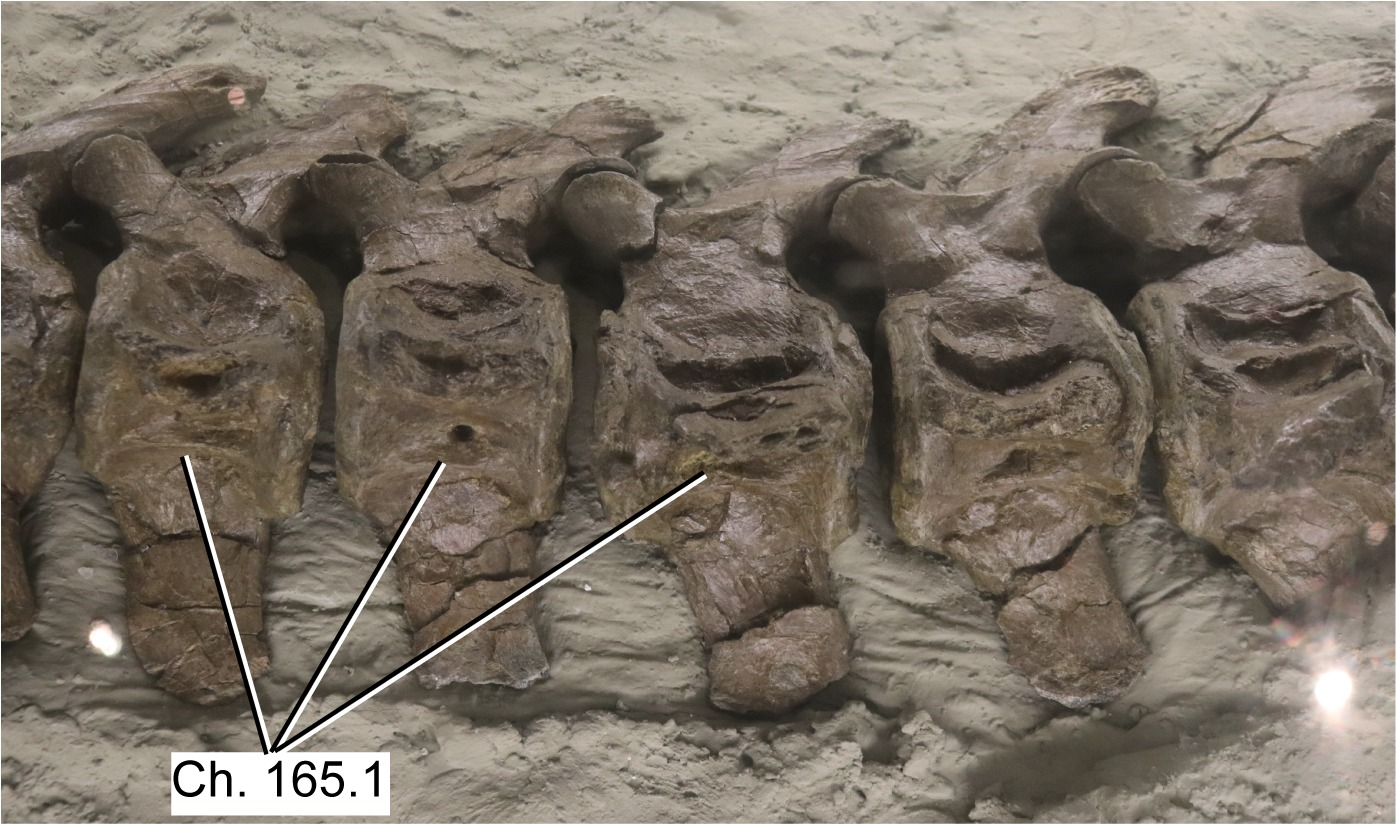

Supplement: S19 Fig — (TIF) [file pone.0255773.s019.tif]

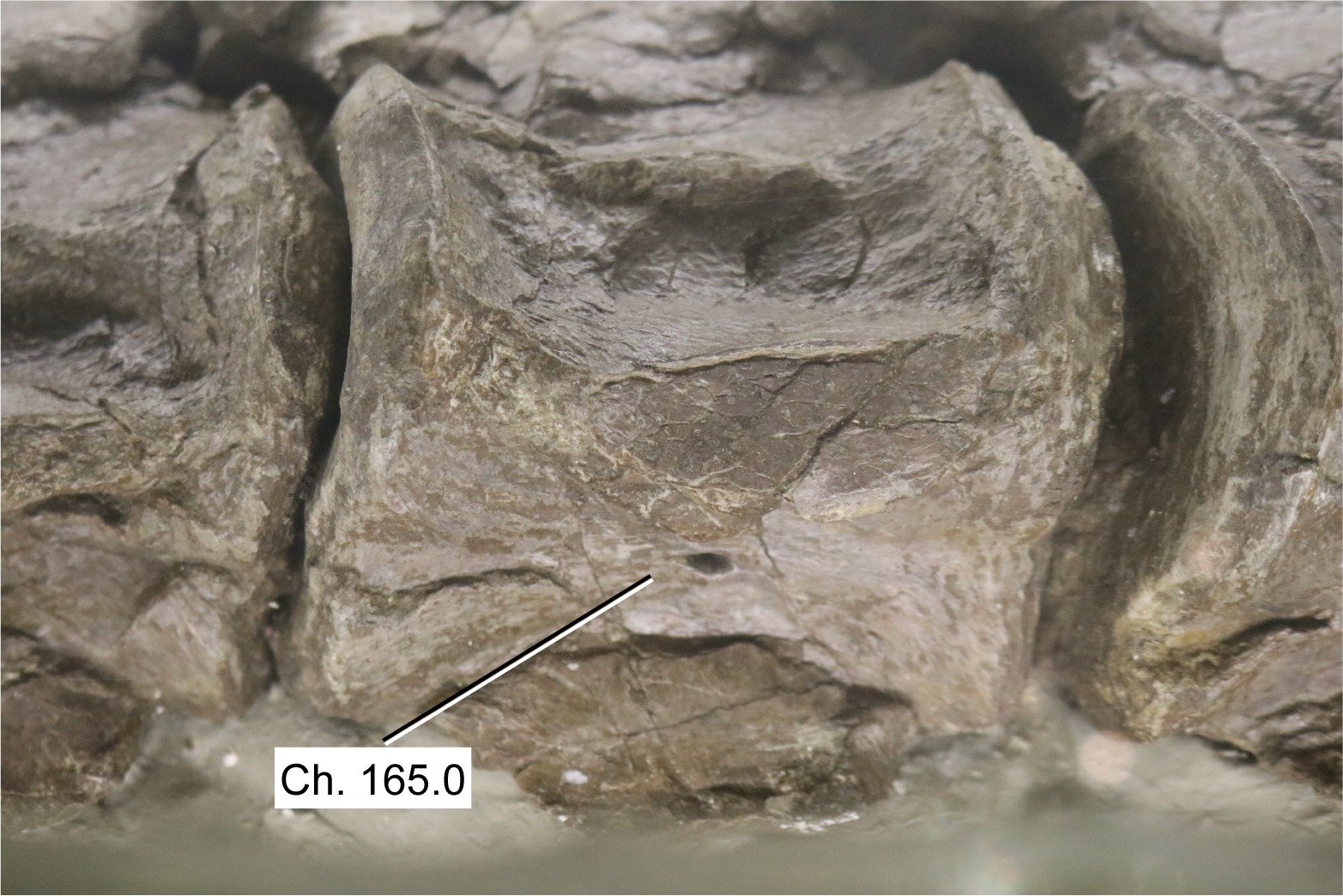

Supplement: S20 Fig — (TIF) [file pone.0255773.s020.tif]

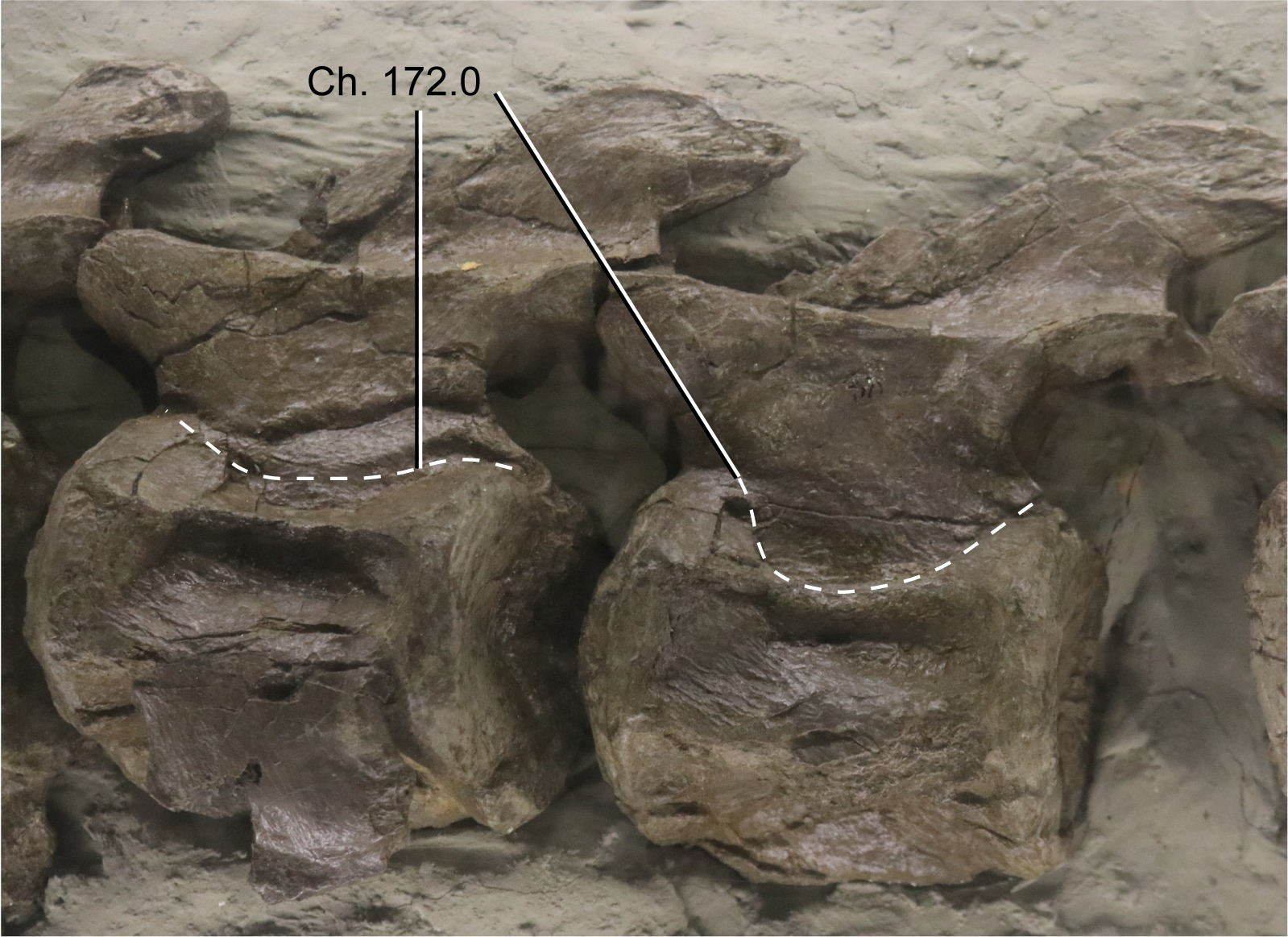

Supplement: S21 Fig — (TIF) [file pone.0255773.s021.tif]

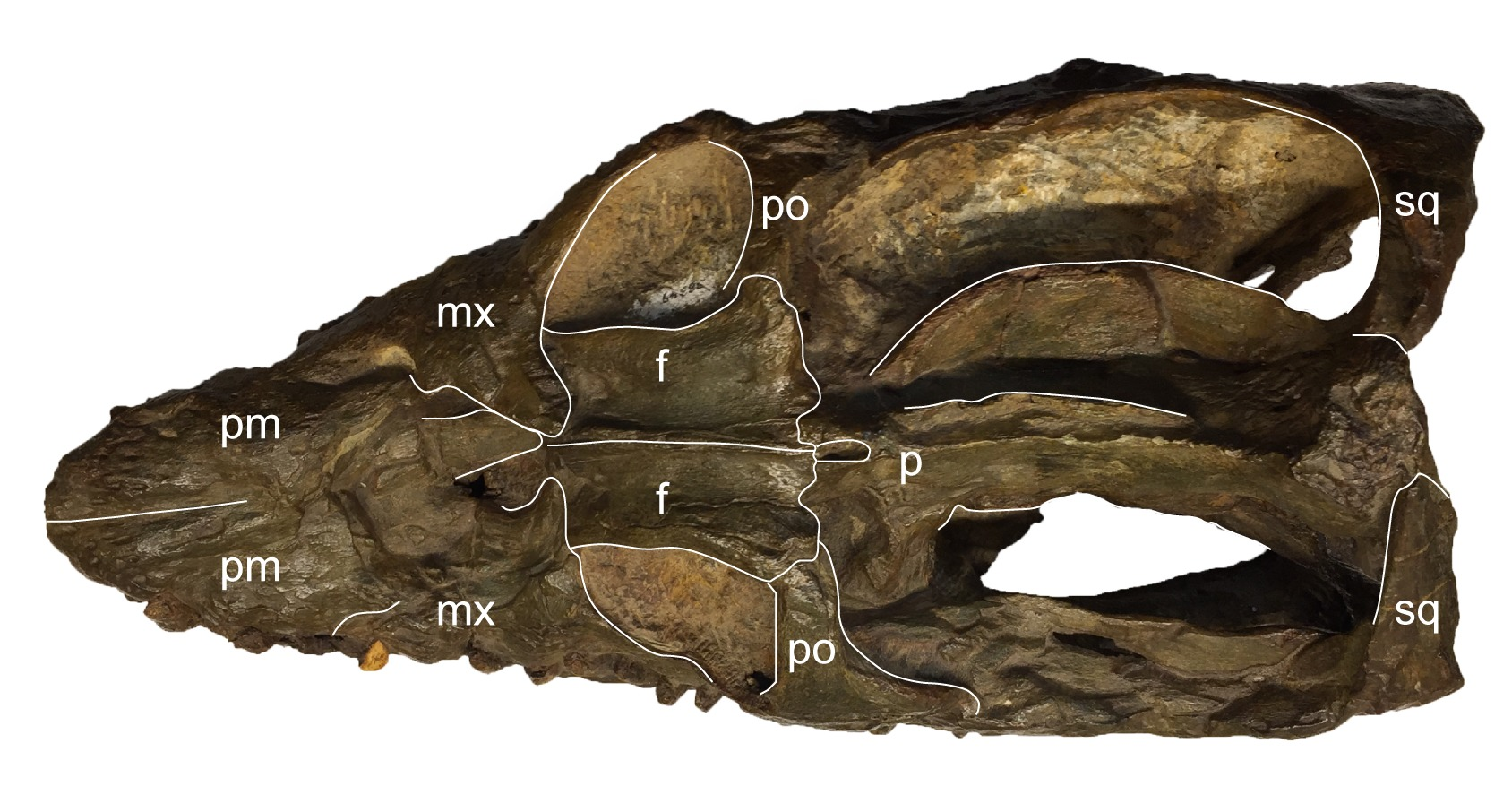

Supplement: S22 Fig — (TIF) [file pone.0255773.s022.tif]

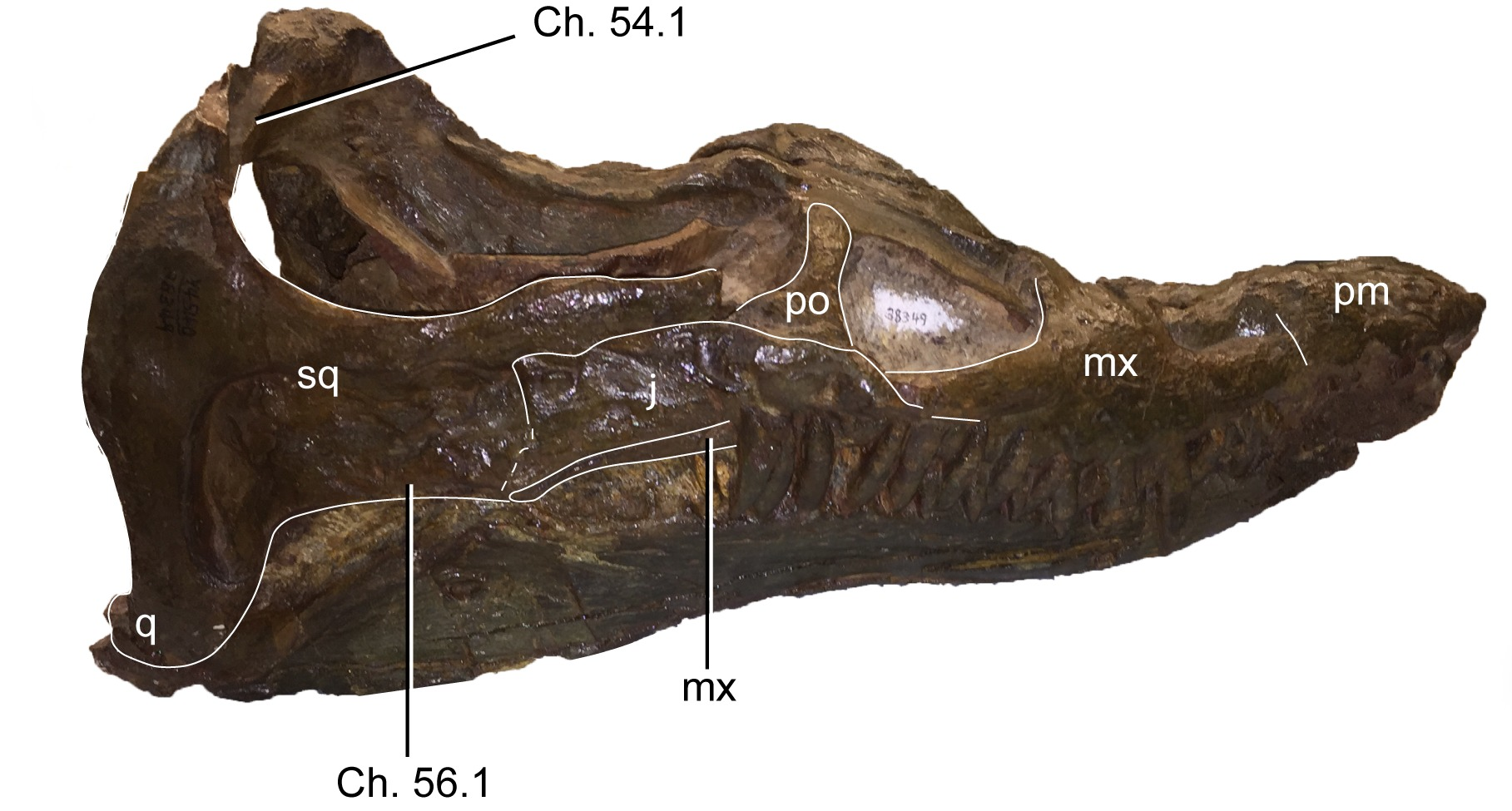

Supplement: S23 Fig — (TIF) [file pone.0255773.s023.tif]

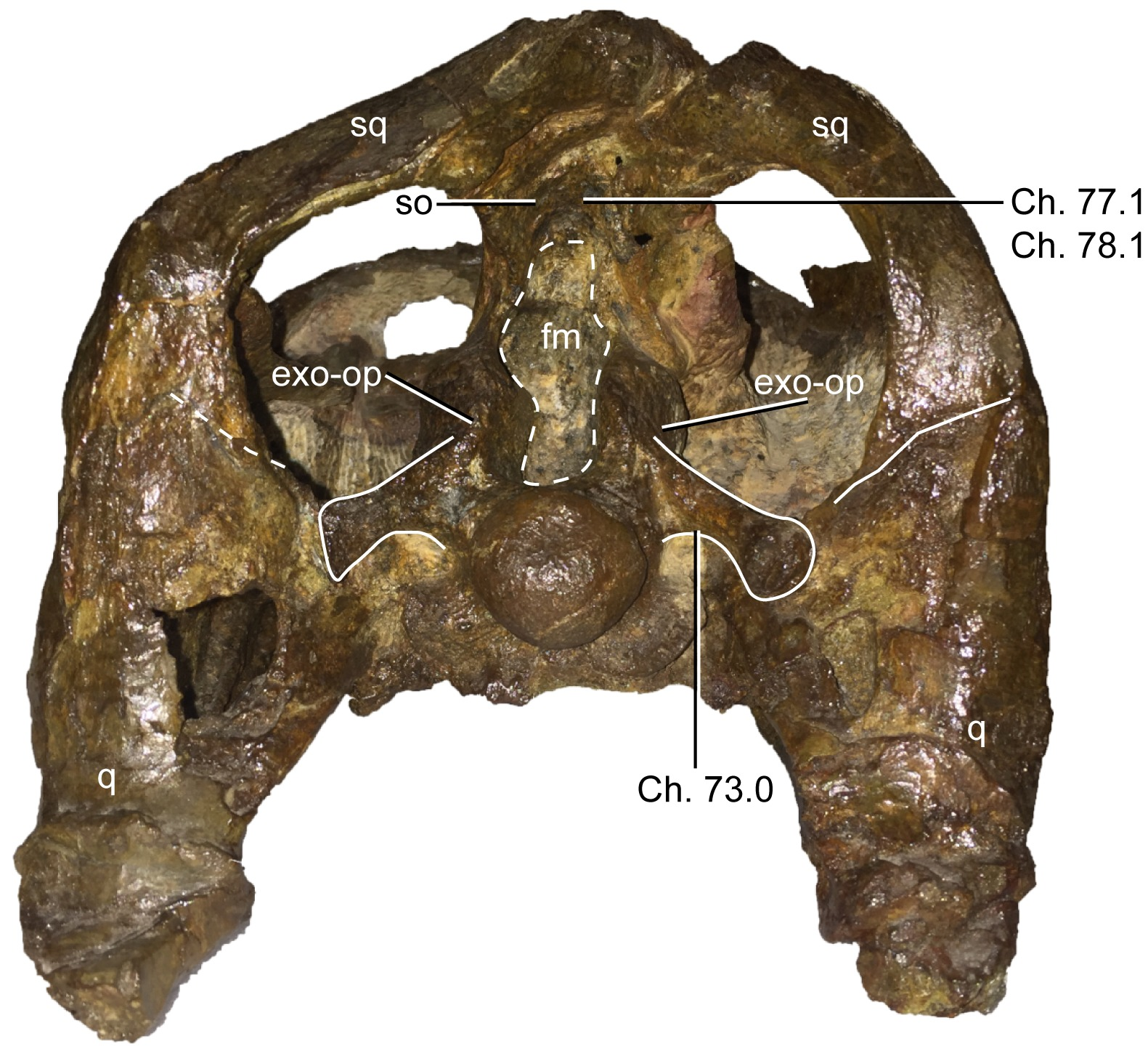

Supplement: S24 Fig — (TIF) [file pone.0255773.s024.tif]

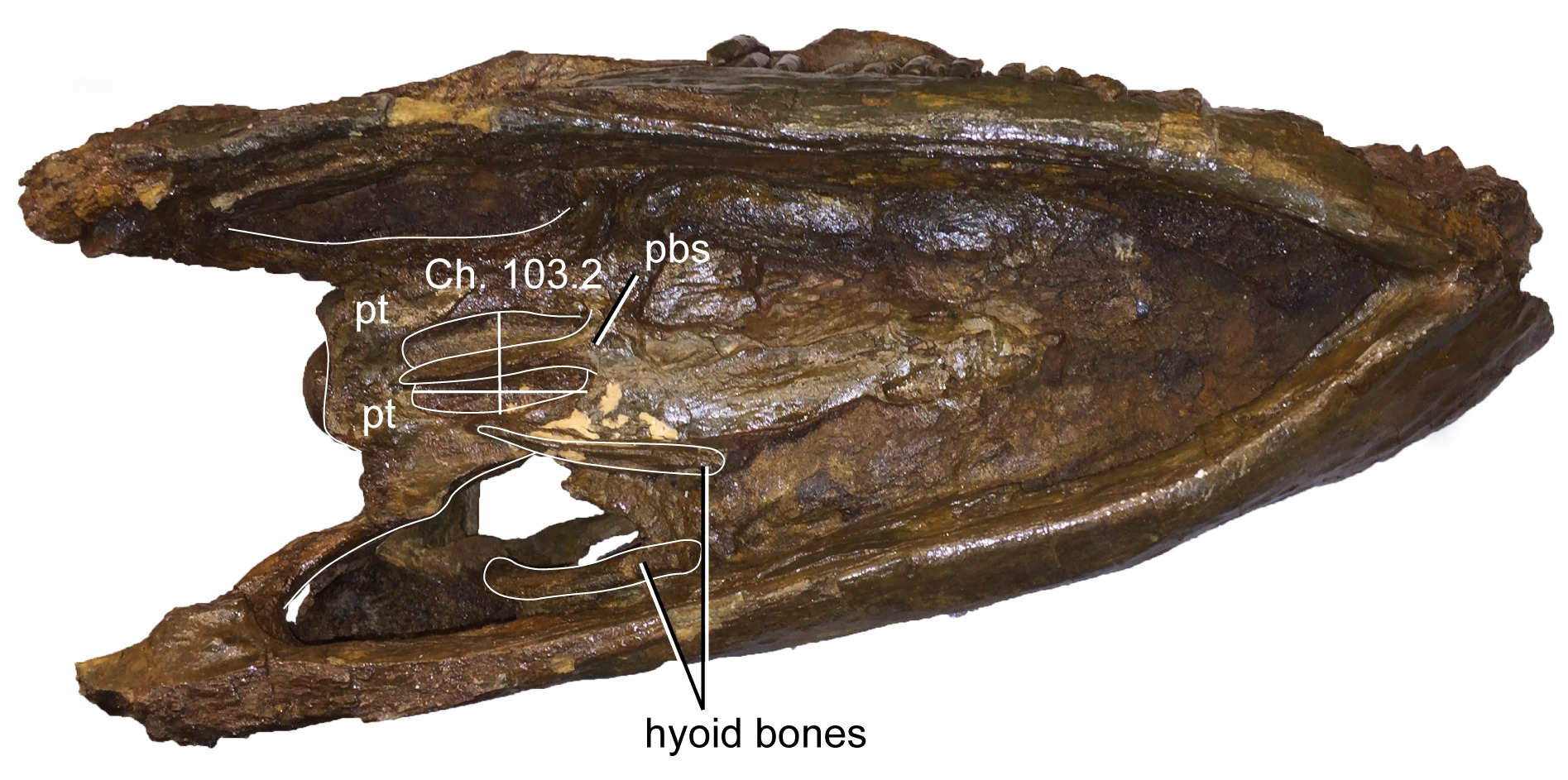

Supplement: S25 Fig — (TIF) [file pone.0255773.s025.tif]

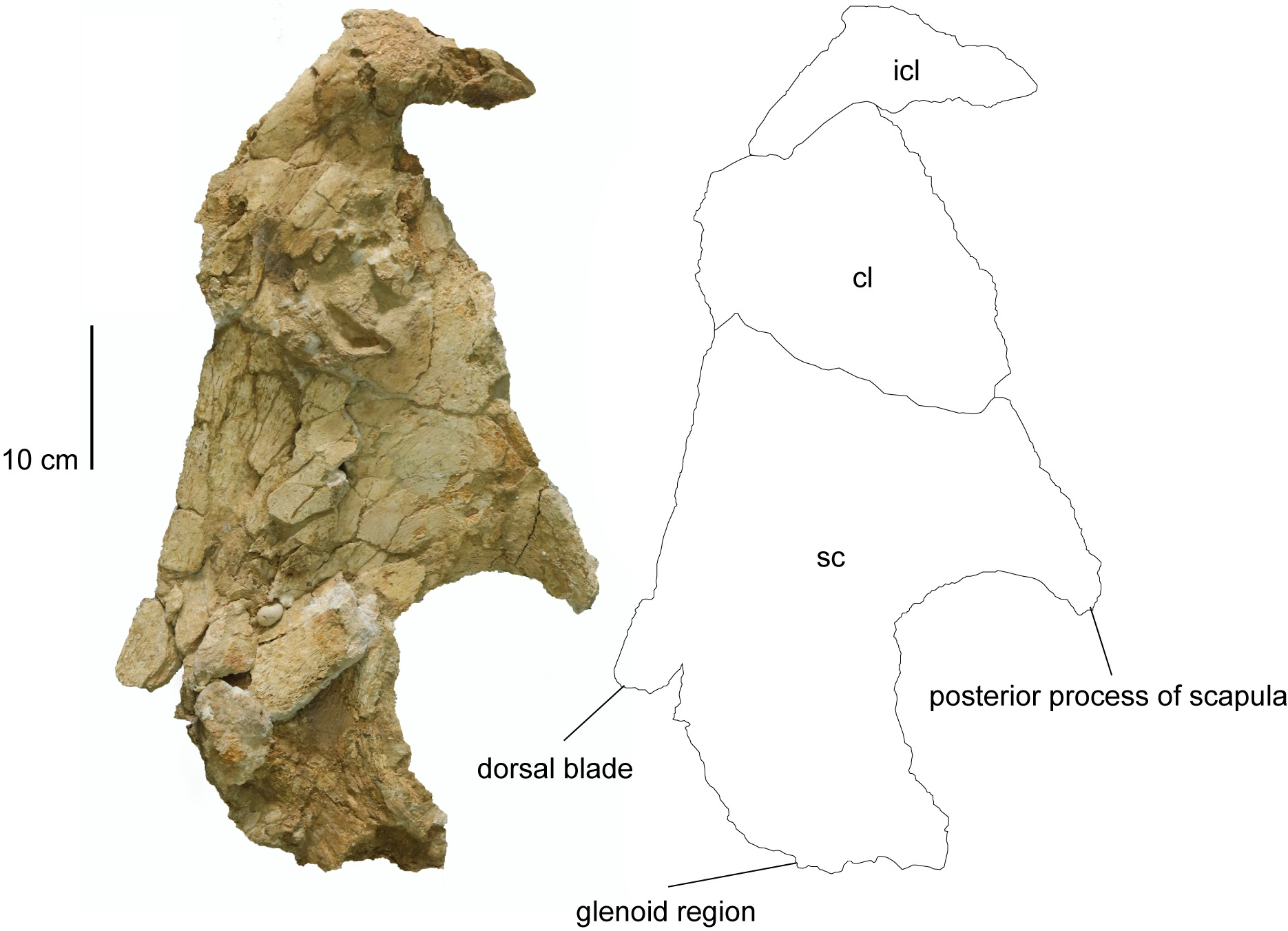

Supplement: S26 Fig — Abbreviations: cl., clavicle; icl., interclavicle; sc., scapula. (TIF) [file pone.0255773.s026.tif]

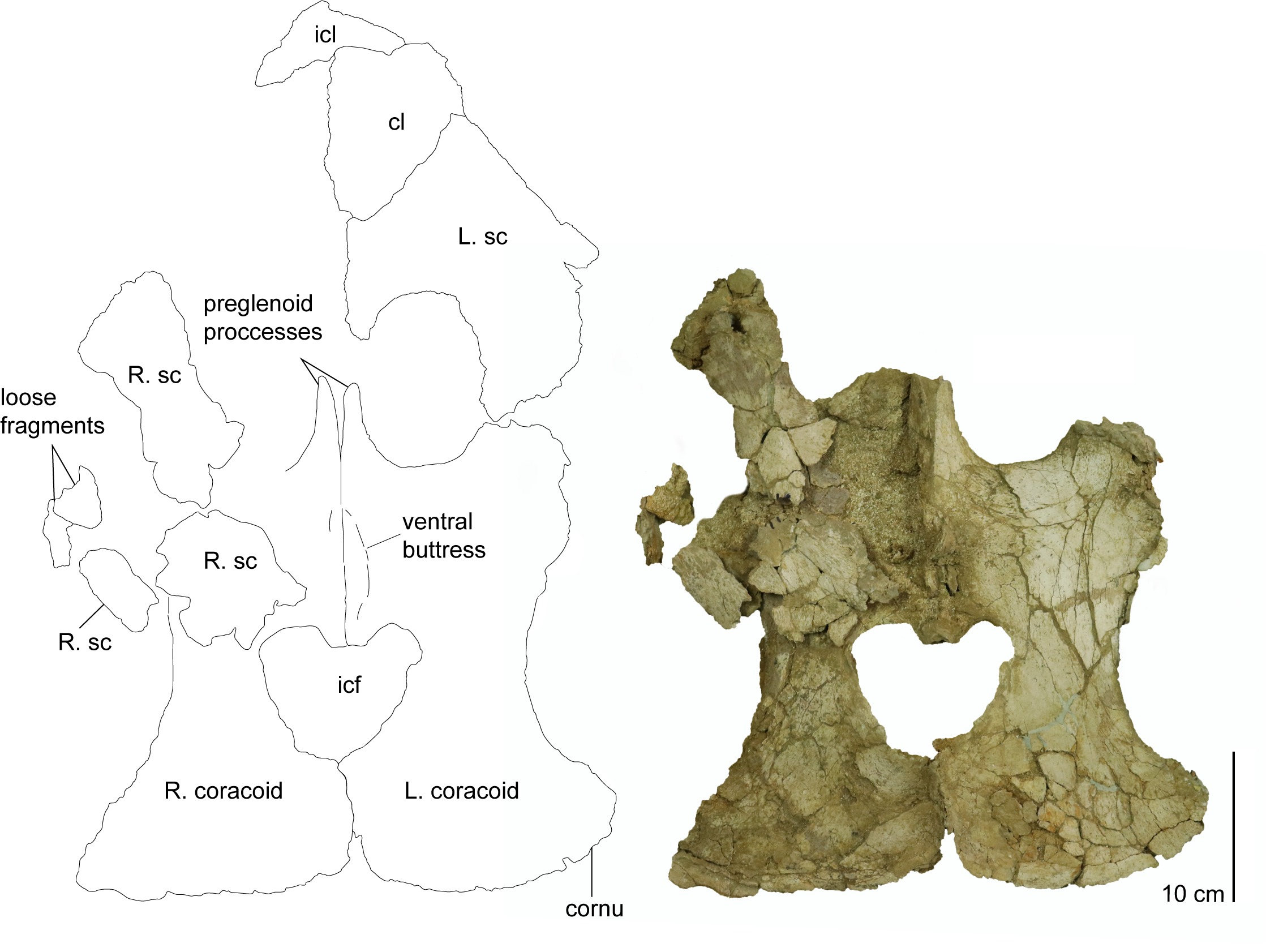

Supplement: S27 Fig — Intercoracoid fenestra is indicated by icf. (TIF) [file pone.0255773.s027.tif]

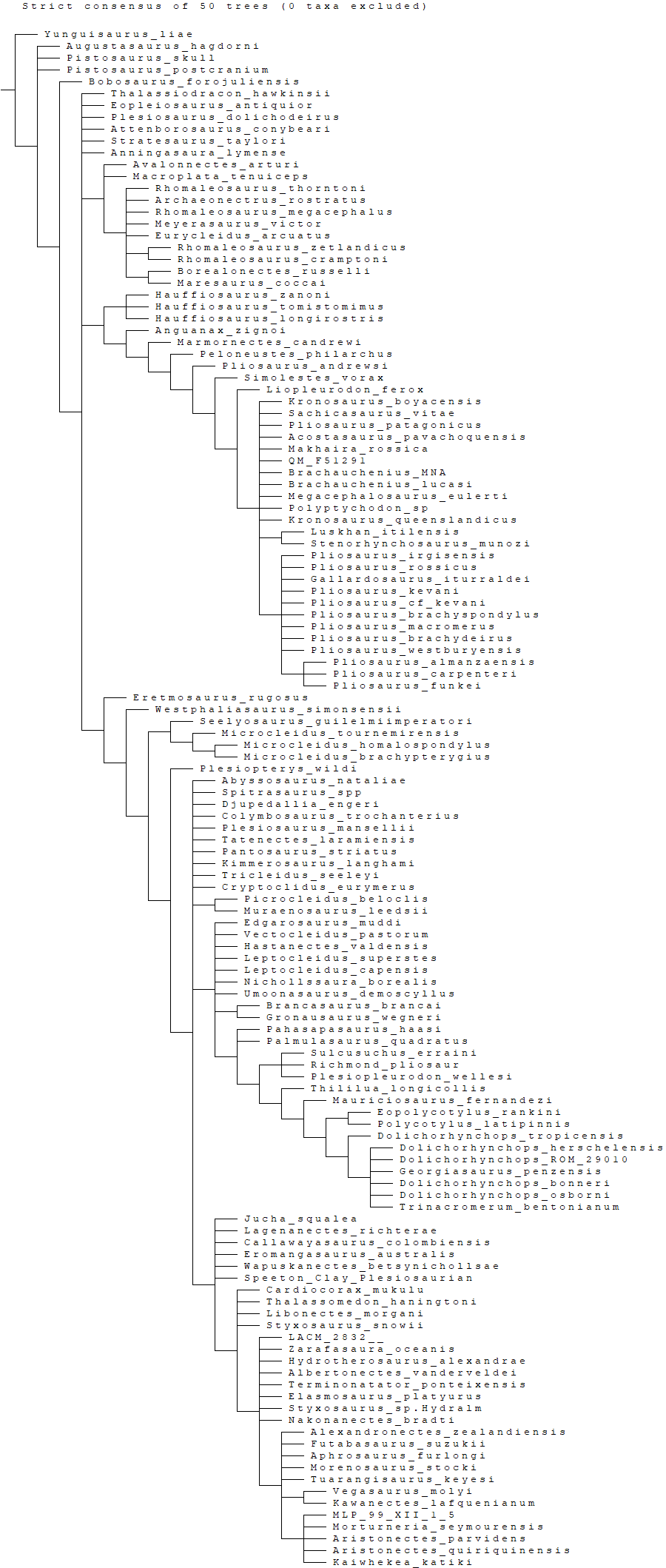

Supplement: S28 Fig — (TIF) [file pone.0255773.s028.tif]
