# Supplementary material for: The cranial anatomy and relationships of Cardiocorax mukulu (Plesiosauria: Elasmosauridae) from Bentiaba, Angola
Source: PLoS One. 2021 Aug 17;16(8):e0255773. doi: 10.1371/journal.pone.0255773 (PMC8370651; doi:10.1371/journal.pone.0255773)
Supplement: S1 File — (DOCX) [file pone.0255773.s029.docx]

The cranial anatomy and relationships of *Cardiocorax mukulu* (Plesiosauria: Elasmosauridae) from Bentiaba, Angola

Miguel P. Marx^1,2,3*^ Octávio Mateus^2,3^ Michael J. Polcyn^1^ Anne S. Schulp^4,5^ A. Olímpio Gonçalves^6^ Louis L. Jacobs^1^

^1^ Huffington Department of Earth Sciences, Southern Methodist University, Dallas, Texas, USA

^2^ GeoBioTec + Faculdade de Ciências e Tecnologia, Universidade Nova de Lisboa, Caparica, Portugal

^3^ Museu da Lourinhã, Lourinhã, Portugal

^4^ Naturalis Biodiversity Center, Leiden, the Netherlands

^5^ Department of Earth Sciences, Faculty of Geosciences, Utrecht University, Utrecht, the Netherlands

^6^ Departamento de Geologia, Faculdade de Ciências, Universidade Agostinho Neto, Luanda, Angola

*Corresponding author

E-mail: [mpmarx1@gmail.com](mailto:mpmarx1@gmail.com) (MM)

**Character changes explained:**

Here we explain the character changes made to elasmosaurid taxa used in our character matrix, modified from Fischer et al. (2020).

In each case we present the character (e.g., Ch. 1) followed by the original score of the character, and then the character score we provide.

Example: Ch. 1; 0 to 1. Here, the original score of 0 for character 1 was changed to 1.

***Libonectes morgani* (SMU SMP 69120 and SMNK-PAL 3978)**

Ch. 5; 0 to 1

The ventral margins of the orbits in SMU SMP 69120 are damaged and thus cannot be assessed for this character. However, a clear convex surface is apparent in left lateral view of the skull of SMNK-PAL 3978 (Fig. 1A in Sachs and Kear, 2017). In right lateral view of the skull of SMNK-PAL 3978 the ventral margin appears rugose and damaged (Fig. 1B in Sachs and Kear, 2017).

Ch. 18; 1 to 0

The dorsomedian ridge of SMU SMP 69120 is steep relative to the surrounding dorsal surface of the premaxilla and thus appears more crest-like. The dorsomedian ridge also does not occupy a significant degree of internarial width (S5 Fig). The internarial width of the dorsomedian ridge in SMNK-PAL 3978 does appear to occupy most of the internarial width on the surface of the premaxillae, although still crest-like (Fig 2A in Sachs and Kear, 2017). The relative width of dorsomedian ridges in these two specimens of *Libonectes morgani* are narrow relative to that of *Thalassomedon haningtoni* (UNSM 50132) which is clearly not crest-like and broad; occupying the entire surface of the premaxillae between the external nares (S2 Fig).

Ch. 26; ? to 1

SMU SMP 69120 does not preserve all of the squamosal along the ventral margin of the temporal bars. SMNK-PAL 3978 exhibits slight contact between the maxilla and squamosal (Fig S6A in Sachs and Kear, 2017). Sachs and Kear (2017) confirm this contact in another specimen of *Libonectes morgani* (SMNS 81783).

Ch. 31; 0 to 1

The posterior margin of the external nares of SMU SMP 69120 are missing the prefrontal (restored in Carpenter, 1997), exposing the frontal which contributes posteriorly and slightly medially to the external nares (S5 Fig). The frontal is interpreted to contribute to the external nares in SMNK-PAL 3978 in Sachs and Kear (Fig. S6B in Sachs and Kear, 2017).

Ch. 37; ? to 0

The jugal of SMNK-PAL 3978 contributes to the posteroventral margin of the orbit (Fig. S6A and S6B in Sachs et al., 2017).

Ch. 38; ? to 0

The jugal of SMNK-PAL 3978 is elongated and extends anteriorly along the ventral margin of the orbit (Fig. S6A and S6B in Sachs et al., 2017).

Ch. 48; ? to 0

The inter-squamosal suture in lateral view of SMNK-PAL 3978 does not appear to rise and is rather flat (Fig. 1A and 1B in Sachs et al., 2017).

Ch. 50; 2 to 3

The sagittal crest of SMNK-PAL 3878 rises steeply above the skull roof above the orbits (S6A in Sachs and Kear, 2017) and is dome-like, similar to *Styxosaurus snowii*, and *Thalassomedon haningtoni* (S2, S3, and S4 Figs).

Ch. 53; 2 to 1

In dorsal view of SMNK-PAL 3978 the dorsal rami of the squamosals extends laterally and are not v-shaped (Fig. 2A in Sachs et al., 2017), as in *Kimmerosaurus langhami* (Brown, 1981), *Kaiwhekea katiki* (Cruickshank and Fordyce, 2002), or *Aristonectes quiriquiensis* (Otero et al., 2014).

Ch. 58; ? to 0

In dorsal and lateral view of SMNK-PAL 3978 the squamosal suture is low and flat (Fig. 1A and 1B and 2A in Sachs et al., 2017).

Ch. 61; 0 to 1.

The dorsal portion of the squamosal in lateral views of SMNK-PAL 3978 are inflected anterodorsally. This characteristic has also been argued for SMU SMP 69120 (Serratos et al., 2017 Supplementary Info).

Ch. 73; 1 to 0

The shaft of the opisthotic is circular and not dorsoventrally flattened in SMU SMP 69120 (S6 Fig).

Ch. 80; ? to 1

The parabasisphenoid does not contribute to the basitubera of SMU SMP 69120. Rather, the parabasisphenoid terminates anterior to the suture formed by the pterygoids beneath the basioccipital (S9 Fig).

Ch. 81; 1 to 0

There is no posterior notch in the clivus apparent between the basisphenoid and basioccipital in our study of SMU SMP 69120. The basisphenoid and basioccipital are fully sutured with no perforations apparent between them.

Ch. 102; 1 to 0

The quadrate process of the pterygoid does not present a lappet ventrally in SMU SMP 69120 (S8 Fig).

Ch. 103; 3 to 2

Serratos et al. (2017) (Supplementary Info) demonstrate that the posterior interpterygoid vacuity exhibits a maximum length to combined width ratio of 1.8-2.5. We confirmed character state with our study of SMU SMP 69120.

Ch. 110; 1 to 0

The ectopterygoid boss length is less than width in SMU SMP 69120 (S9 Fig).

Ch. 111; 1 to 0

Benson and Druckenmiller (2014) do not provide a method for which to measure bowing of the mandible. However, bowing of the mandible can be measured using the approach of Druckenmiller and Russell (2008) who identify a bowed mandible as one that extends laterally beyond a line drawn between the glenoid of the mandible and the symphysis. Serratos et al. (2017) demonstrate that bowing is present in SMU SMP 69120 and we confirm this in our study of SMU SMP 69120.

Ch. 115; 0 to [01]

The coronoid eminence of SMU SMP 69120 is divided equally between the dentary and the surangular (S2 Fig in Sachs and Kear, 2017).

Ch. 123; ? to 0

The retroarticular process of SMU SMP 69120 is not currently preserved, however plate 3 in Welles (1949) this portion of the mandible is preserved and is not significantly inflected medially.

Ch. 124; ? to 0

The dorsal rim of the lingual mandibular fenestra of SMU SMP 69120 appears to be formed by the prearticular (Fig. 15 in O’Keefe, 2001).

Ch. 132; 0 to 1

The premaxillary teeth of SMU SMP 69120 are considered heterodont because the anterior-most pair of premaxillary alveoli is smaller relative to the posterior premaxillary alveoli (S7 Fig).

Ch. 153; 2 to [123]

This character measures the proportions of anterior-middle cervical centra (L/H). In SMU SMP 69120, state 1 is present in cervical vertebra 8, state 2 is present in cervical vertebra 15, and state 3 is present in cervical 17 based on the measurements provided by Welles (1949) (Table 1).

Ch. 155; 2 to [12]

The anterior cervical vertebrae of SMU SMP 69120 and SMNK-PAL 3978 exhibit a ventral notch; the posterior cervical vertebrae of SMU SMP 69120 also exhibit this feature in the posterior cervical vertebrae (Sachs and Kear, 2015).

Ch. 157; ? to 0

The anterior cervical neural spines in SMNK-PAL 3978 (Figure 3B and 4B in Sachs and Kear, 2017) appear to be curved posterodorsally.

Ch. 158; 2 to 3

Posterior cervical neural spines (preserved in SMNK-PAL 3978) are oriented straight dorsally (Sachs and Kear, 2017). Sachs and Kear (2017) describe the caudad cervical neural spines as being upright.

Ch. 173; 2 to 3

Character modified by O’Gorman (2020). Anterior cervical vertebrae B/H greater than 1.3.

Ch. 199; ? to1

Fig. 2 in Welles (1949) shows the scapula of SMU SMP 69120 as being triradiate with an extensive ventral plate.

Ch. 207; ? to1

Fig. 2 in Welles (1949) presents the cornu of the coracoid extending to the level of the glenoid.

Ch. 212; 0 to ?

The dimensions of the preglenoid process of the coracoid are unknown in SMU SMP 69120 (Welles, 1949).

Ch. 223; ? to 1

In Fig 8B of Sachs and Kear (2017) the dorsal margin of the ilium is exposed and presents anterior and posterior margins that appear to be equally expanded.

Ch. 225; ? to 1

In Fig 8B of Sachs and Kear (2017) a tubercle is present on the posterior margin of the ilium.

Ch. 241; 1 to 0

The humerus of SMNK-PAL 3978 is short and robust. Sachs and Kear (2017) report the proximodistal length of the humerus as 229 mm (correcting for breakage) and the proximodistal length of the femur as 313 mm [0]. 313/229 = 0.73.

Ch. 244; ? to 4

The proximodistal length of the humerus of SMNK-PAL 3978 is 313 mm and its distal width is 261 mm based on Sachs and Kear (2017). 313/261 = 1.2 [4].

Ch. 248; 0 to 1

Changed to humerus by O’Gorman (2020). The angle between the epipodial facets is 180° in dorsal view (Fig. 7B in Sachs et al., 2017).

Ch. 251; ? to 2

The proximodistal length of the femur of SMNK-PAL 3978 is 313 mm and the distal width is 179 mm based on Sachs and Kear (2017). 313/179 =1.75 [2].

***Styxosaurus snowii* (KUVP 1301)**

Ch. 1; 0 to ?

This character cannot be scored because the cranium of KUVP 1301 is compressed.

Ch. 2; 0 to ?

Due to compression of the skull of KUVP 1301, transverse constriction of the rostrum cannot be scored.

Ch. 4; 0 to ?

The length of the skull is typically measured from the tip of the premaxillae to the occipital condyle in plesiosaurians (Welles, 1943; Druckenmiller and Russell, 2008; Serratos et al., 2017; Allemand et al., 2017). The occipital condyle is obscured in KUVP 1301, making this character un-scorable.

Ch. 22; ? to 2

The premaxillae of KUVP 1301 become constricted at the level of the external nares and then return to their original width posterior to the external nares (S4 Fig).

Ch.29; ? to 0

The premaxilla of KUVP 1301 extends posteriorly to contact the medial margin of the external naries (S4 Fig).

Ch. 35; ? to 1

The prefrontal contacts the external naris of KUVP 1301. A distinct suture confidently identifies the prefrontal from surrounding cranial elements (S10 Fig).

Ch. 48; ? to 0

The squamosal suture is low in KUVP 1301, and we agree with the score for this character in the Fischer et al. (2020) matrix.

Ch. 51; ? to 2

The parietal ornamentation adjacent to the area for the pineal foramen (pineal foramen not present in KUVP 1301) is a raised ridge (S4 Fig).

Ch. 60; 0 to ?

The medial contact between the squamosal and quadrate in KUVP 1301 is obscured.

Ch. 110; 0 to ?

The ectopterygoid boss of KUVP 1301 is distorted, with compression along the mediolateral axis (S11 Fig).

Ch. 111; 0 to ?

The mandible of KUVP 1301 is compressed mediolaterally, and thus bowing could not be accurately quantified.

Ch. 114; 0 to 1

The ventral margin of the mandibular symphysis of KUVP 1301 exhibits a sharp keel (S12 Fig).

Ch. 116; 0 to 1

The left retroarticular process (measured from the posterior limit of the process to the posterior limit of the mandibular glenoid: 42.7 mm) is longer than the left mandibular glenoid of KUVP 1301 (20.9 mm). We note that the left mandibular glenoid in KUVP 1301 is partly obscured, thus we also took measurement of the better right mandibular glenoid for comparison (32.5 mm).

Ch. 126; 1 to 2

The angular of KUVP 1301 is elongated and contributes to the mandibular symphysis (S3 Fig).

Ch. 139; 3 to 2

The teeth are ellipsoidal; state 3 is a modification from Madzia and Cau (2020).

Ch. 140; 0 to ?

Compression of the cranium of KUVP 1301 prevents collection of accurate dimensional data of the diameter of the first alveolus.

Ch. 143; ? to 1

The axial rib facet is single headed in KUVP 1301 (S13 Fig).

Ch. 147; ? to 0

The hypophyseal ridge of the atlas-axis complex extends across the atlas and axis centrum (S14 Fig).

Ch. 153; 3 to [23]

A personal visit confirms the L/H ratio of the anterior cervical vertebrae of KUVP 1301 do not reach a ratio greater than 1.5. It is not until cervical vertebra 20 that the L/H ratio is greater than 1.5. The vertebrae anterior to cervical vertebra 20 exhibit a ratio below 1.5.

Ch. 172; ? to 0

The suture between the neural arches and the centra in KUVP 1301 is rounded (S15 Fig).

Ch. 281; 1 to ?

KUVP 1301 does not preserve an ilium.

***Thalassomedon haningtoni* (DMNS 1588 and UNSM 50132)**

Ch. 18; 0 to 2

A posterior mound or boss is formed by the premaxilla in UNSM 50132 (S16 Fig).

Ch. 22; ? to 1

The premaxilla is constricted by the external naris in UNSM 50132 and does not expand to original width posterior to external naris (S2 Fig).

Ch. 31; ? to 1

The frontal of UNSM 50132 participates in the rim of the external naris (S2 Fig).

Ch. 32; 1 to ?

The premaxilla extends posteriorly to contact the parietal; thus, this character cannot be scored.

Ch. 35; 1 to ?

The prefrontal of UNSM 50132 could not be confidently identified (S2 Fig).

Ch. 36; 0 to ?

The postfrontal of UNSM 50132 could not be confidently identified (S2 Fig).

Ch. 45; 1 to ?

A pineal foramen is not present in UNSM 50132 and is not apparent in DMNS 1588 (Sachs et al., 2021).

Ch. 48; ? to 0

The inter-squamosal suture of UNSM 50132 is low and rounded (S17 Fig).

Ch. 50; 2 to 3

The sagittal crest of UNSM 50132 rises high above dorsomedial margin of the orbits, similar to KUVP 1301 and SMNK-PAL 3978 (S2 Fig.).

Ch. 51; ? to 2

Adjacent to the area of the pineal foramen is a tall ridge formed by the parietal (S2 Fig).

Ch. 52; ? to 1

The parietal extends anteriorly to the orbital mid-length of UNSM 50132 (S2 Fig).

Ch. 53; ? to 1

The dorsal rami of the squamosals extend laterally from their dorsal contact with the parietal in UNSM 50132.

Ch. 54; ? to 1

The squamosal arches of UNSM 50132 are anteroposteriorly compressed (S19 Fig).

Ch. 55; 0 to 1

The temporal bar is not significantly arched (S2 Fig), and we agree with the score present in the Fischer et al. (2020) matrix.

Ch. 58; ? to 0

The inter-squamosal suture of UNSM 50132 is flat and does not rise significantly (S17 Fig).

Ch. 59; 0 to ?

Contact between the quadrate and squamosal in UNSM 50132 is not entirely exposed, making this character difficult to score.

Ch. 139; 3 to 2

Character changed to fit the matrix of Fischer et al. (2020).

Ch. 148; 2 to 1

This character state was changed to state ‘1’, as an atlas rib is present in the atlas-axis complex of UNSM 50132 and appears to be co-ossified with the atlas. A photograph showing this state was difficult to provide.

Ch. 153; 2 to [12]

The L/H ratio of several anterior cervical vertebrae of DMNS 1588 are approximately as long as high, in addition to the length to height ratio being 1.1-1.5 (Welles, 1943).

Ch. 158; ? to 3

The posterior cervical neural spines of UNSM 50132 are oriented straight dorsally from the centrum (S18 Fig). This was a new character state introduced by O’Gorman (2020).

Ch. 165; ? to [01]

The anterior cervical vertebrae of UNSM 50132 exhibits a rounded median ventral keel, while the posterior cervical vertebrae exhibit no ventral keel (S19 and S20 Fig).

Ch. 169; 0 to 1

The prezygapophyseal facets of UNSM 50132 are smoothly concave to receive the convex facet of the postzygapophyses based on the first-hand observation of UNSM 50132.

Ch. 172; ? to 0

The neurocentral suture of the cervical vertebrae of UNSM 50132 is rounded (S21 Fig).

Ch. 173; 0 to 1

Several vertebrae in DMNS 1588 exhibit several vertebrae with state ‘1’ (Welles, 1943).

Ch. 253; 1 to 0

This character was modified by O’Gorman (2020) and so we kept the original character score from O’Gorman (2020).

***Callawayasaurus colombiensis* (UCMP 38349)**

Ch. 5; 1 to ?

The margins of both the left and right orbit are damaged, making an honest scoring of this character difficult.

Ch. 15; [12] to 0

The premaxilla of UCMP 38349 does not overlap the frontals (S22 Fig).

Ch. 31; ? to 0

The frontal of UCMP 38349 does not participate with the external naris (S22 Fig).

Ch. 34; ? to 0

There is no lacrimal present in UCMP 38349, rather the maxilla contributes to the orbital margin (S22 and S23 Fig).

Ch. 37; ? to 0

Jugal participates in orbital margin (S23 Fig).

Ch. 38; ? to 0

The jugal of UCMP 38349 is elongated and extends anterior to the postorbital (S23) (Welles, 1962).

Ch. 39; ? to 0

The jugal of UCMP 38349 is embayed by the orbit (S23 Fig).

Ch. 40; ? to 1

The jugal makes contact with the squamosal in UCMP 38349 (S23 Fig).

Ch. 41; ? to 0

The jugal-squamosal contact is sub-vertical in UCMP 38349 (S23 Fig).

Ch. 46; 0 to 1

The pineal foramen is located just posterior to the postorbital bar in UCMP 38349 (S22 Fig).

Ch. 50; 2 to 3

The sagittal crest is tall in UCMP 38349 and makes a dome shape in dorsal view (crest is broken off and is laying on its right side) (Welles, 1962).

Ch. 54; 0 to 1

The squamosal rami of UCMP 38349 are anteroposteriorly compressed (S23 Fig).

Ch. 56; 0 to 1

The temporal bar of UCMP 38349 is high; 2/3 or greater the height of the orbit (S23 Fig).

Ch. 62; 0 to 1

The position of the tooth row in UCMP 38349 is much higher than that of the glenoid (S23 Fig).

Ch. 73; 1 to 0

The shaft of the paraoccipital process of UCMP 38349 is sub-circular in cross-section (S24 Fig).

Ch. 77; ? to 1

There is no posteromedian ridge of the supraoccipital present in UCMP 38349 (S24 Fig).

Ch. 78; ? to 1

There is no posteromedian process of the supraoccipital present in UCMP 38349 (S24 Fig).

Ch. 80; ? to 1

The parabasisphenoid does not contribute to the basioccipital tuberosities in UCMP 38349 (S25 Fig).

Ch. 85; 1 to ?

The cultriform process of the parasphenoid is not clearly visible in view of the palate of UCMP 38349 (S25 Fig).

Ch. 86; 1 to ?

The anterior half of the palate of UCMP 38349 is not clearly visible due to crushing and a gypsiferous matrix (Welles, 1962).

Ch. 87; 0 to ?

The anterior half of the palate of UCMP 38349 is not clearly visible due to crushing and a gypsiferous matrix (Welles, 1962).

Ch. 88; 0 to ?

The anterior half of the palate of UCMP 38349 is not clearly visible due to crushing and a gypsiferous matrix (Welles, 1962).

Ch. 89; 1 to ?

The anterior half of the palate of UCMP 38349 is not clearly visible due to crushing and a gypsiferous matrix (Welles, 1962).

Ch. 94; 0 to ?

The anterior half of the palate of UCMP 38349 is not clearly visible due to crushing and a gypsiferous matrix (Welles, 1962). The palate of UCMP 125328 is incomplete (Páramo-Fonseca et al., 2019).

Ch. 98; ? to 1

The pterygoids of UCMP 38349 form a sutural contact posterior to the posterior interpterygoid fenestra (S25 Fig).

Ch. 103; 1 to 2

The posterior interpterygoid fenestra of UCMP 38349 provides a ratio slightly greater than 1.8 (S25 Fig).

Ch. 120; 1 to 2

The anterior part of the glenoid is squared-off in ventral view (S25 Fig).

Ch. 132; 0 to 1

O’Gorman (2020) has character 132 scored as homodont, however, *Callawayasaurus colombiensis* is scored for a diameter of the first alveolus being less than half the diameter of the third alveolus for character 140 by O’Gorman (2020). Additionally, Welles (1962) describes the first premaxillary pair as being very small, with posterior premaxillary teeth being larger.

Ch. 133; 0 to 1

UCMP 38349 exhibits anisodont maxillary dentition, as indicated by the presence of a maxillary fang and proceeding smaller maxillary teeth (S23 Fig).

Ch. 153; 2 to [12]

Cervical vertebrae 14 and 15 exhibit L/H ratios of approximately 1 (Welles, 1962).

**References**

Allemand, R., Bardet, N., Houssaye, A., and Vincent, P. 2017. Virtual reexamination of a plesiosaurian specimen (Reptilia, Plesiosauria) from the Late Cretaceous (Turonian) of Goulmima, Morocco, using computed tomography. *Journal of Vertebrate Paleontology*, 37, e1325894.

Benson, R.B.J., Druckenmiller, P.S. 2014. Faunal turnover of marine tetrapods during the Jurassic-Cretaceous transition. *Biological Reviews* 89, 1-23.

Brown, D.S., 1981. The English Upper Jurassic Plesiosauroidea (Reptilia) and a review of the phylogeny and classification of the Plesiosauria. *Bulletin of the British Museum* (Natural History), Geology Series 35, 253-347.

Carpenter, K., 1997. Comparative cranial anatomy of two North American plesiosaurs. In: Callaway, J.M., Nicholls, E.L. (editors), *Ancient Marine Reptiles*. Academic Press, San Diego, pp.148-173.

Cruickshank, A.R.I. and Fordyce, R.E. 2002. A new marine reptile (Sauropterygia) from New Zealand: further evidence for a late Cretaceous austral radiation of cryptoclidid plesiosaurs. *Palaeontology* 45, 557-575.

Druckenmiller, P.S., Russell, A.P., 2006. A new elasmosaurid plesiosaur (Reptilia: Sauropterygia) from the Lower Cretaceous Clearwater Formation, northeastern Alberta, Canada. *Paludicola* 5, 184-199.

Fischer, V., Zverkov, N.G., Arkhangelsky, M.S., Stenshin, I.M., Blagovetshensky, I.V., Uspensky, G.N., 2020. A new elasmosaurid plesiosaurian from the Early Cretaceous of Russia marks an early attempt at neck elongation. *Zoological Journal of the Linnean Society*. <https://doi.org/10.1093/zoolinnean/zlaa103>.

Madzia, D., Cau, A., 2020. Estimating the evolutionary rates in mosasauroids and plesiosaurs: discussion of niche occupation in Late Cretaceous seas. *Peer J* 8, e8941.

O’Gorman, J.P., 2020. Elasmosaurid phylogeny and paleobiogeography, with a reappraisal of *Aphrosaurus furlongi* from the Maastrichtian of the Moreno Formation. *Journal of Vertebrate Paleontology* 39, e1692025.

O’Keefe, F.R., 2001. A cladistic analysis and taxonomic revision of the Plesiosauria (Reptilia: Sauropterygia). *Acta Zoologica Fennica* 213, 1-63.

Otero, R.A., Soto-Acuña, S., O’Keefe, F.R., O’Gorman, J.P., Stinnesbeck, W.S., Suárez, M.E. Rubilar-Rogers, D., Salazar, C., Quinzio-Sinn, L.A., 2014. *Aristonectes quiriquiensis*, sp. nov., a new highly derived elasmosaurid from the upper Maastrichtian of central Chile. *Journal of Vertebrate Paleontology* 34, 100-125.

Páramo-Fonseca, M.E., O’Gorman, J.P., Gasparini, Z., Padilla, S., Parra-Ruge, M.L. 2019. A new late Aptian elasmosaurid from the Paja Formation, Villa de Leiva, Colombia. *Cretaceous Research* 99, 30-40.

Sachs, S., and Kear, B.P., 2015. Postcranium of the paradigm elasmosaurid plesiosaurian *Libonectes morgani* (Welles, 1949). *Geological Magazine* 152, 694-710.

Sachs, S., Kear, B.P. 2017. Redescription of the elasmosaurid *Libonectes atlasense*, from the Upper Cretaceous of Morocco. *Cretaceous Research* 74, 205-222.

Sachs, S., Lindgren, J., Madzia, D., Kear, B.P. 2021. Cranial osteology of the mid-Cretaceous elasmosaurid *Thalassomedon haningtoni* from the Western Interior Seaway of North America. *Cretaceous Research* 123, 104769.

Serratos, D.J., Druckenmiller, P., Benson, R.B., 2017. A new elasmosaurid (Sauropterygia, Plesiosauria) from the Bearpaw Shale (Late Cretaceous, Maastrichtian) of Montana demonstrates multiple evolutionary reductions of neck length within Elasmosauridae. *Journal of Vertebrate Paleontology* 37, e1278608.

Welles, S.P., 1943. Elasmosaurid plesiosaurs with a description of new material from California and Colorado. *University of California Publications in Geological Sciences* 13, 125-254.

Welles, S.P. 1949. A new elasmosaur from the Eagle Ford Shale of Texas. Part I. Systematic description. *Fondren Science Series* 1,1-28.

Welles S.P., 1962. A new species of elasmosaur from the Aptian of Colombia and a review of the Cretaceous plesiosaurs. *University of California Publications in Geological Sciences* 44, 1-96.
